# Utilizing aggregation operators based on q-rung orthopair neutrosophic soft sets and their applications in multi-attributes decision making problems

**DOI:** 10.1016/j.heliyon.2024.e35059

**Published:** 2024-07-26

**Authors:** Sumbal Ali, Asad Ali, Ahmad Bin Azim, Ahmad Aloqaily, Nabil Mlaiki

**Affiliations:** a Department of Mathematics and Statistics, Hazara University, Mansehra, 21300, Khyber Pakhtunkhwa, Pakistan; b Department of Mathematics and Sciences, Prince Sultan University, Riyadh, 11587, Saudi Arabia; c School of Computer, Data and Mathematical Sciences, Western Sydney University, Sydney, 2150, Australia

**Keywords:** q-rung orthopair neutrosophic soft set, Operational properties of q-RONS_f_S_s_, q-rung orthopair neutrosophic soft weighted averaging operator, q-rung orthopair neutrosophic soft ordered weighted averaging operator, q-rung orthopair neutrosophic soft weighted geometric, Q-rung orthopair neutrosophic soft ordered weighted geometric, Decision-making and optimization

## Abstract

Neutrosophic sets provide greater versatility in dealing with a variety of uncertainties, including independent, partially independent, and entirely dependent scenarios, which q-ROF soft sets cannot handle. Indeterminacy, on the other hand, is ignored completely or partially by q-ROF soft sets. To address this issue, this study offers a unique novel concept as known as q-RONSS, which combines neutrosophic set with q-ROF soft set. This technique addresses vagueness using a set of truth, indeterminacy, and false membership degrees associated with the parametrization tool, with the condition that the sum of the qth power of the truth, indeterminacy, and false membership degrees be less than or equal to one. In addition, this study outlines operational laws for the suggested structure. The main purpose this article is to define some averaging and geometric operators based on the q-rung orthopair neutrosophic soft set. Furthermore, this article provides a step-by-step method and a mathematical model for the suggested techniques. To solve a MADM issue, this research article proposes a numerical example of people selection for a specific position in a real estate business based on a variety of criteria. Finally, to demonstrate the proposed model's superiority and authenticity, this article performs several analyses, including sensitivity analysis, to address the reliability and influence of various parameter "q" values on the alternatives and the ultimate ranking outcomes using the averaging and geometric operators. A comparison of the proposed operators to current operators demonstrates the validity of the proposed structure. Furthermore, a comparison of the proposed structure to current theories demonstrates its superiority by overcoming their limits and offering a more flexible and adaptable framework. Finally, this study reviews the findings and consequences of our research.

## 1 Introduction

In daily situations, decision-making (DM) is a complicated process that entails analysing several possibilities and selecting the optimal alternative based on decision makers' judgment. In the past, for the assessment of data decision makers only used real numbers, but with time experts assigned real numbers to evaluate the alternatives for decreasing the complexity of MADM problem.

Zadeh [1] established a strong mathematical framework called the fuzzy set in 1965. Fuzzy set have a wild application in various field see Refs. [[2], [3], [4]]. In 1975, Zadeh [5] provided intervals to each element with a broader range and developed interval-valued fuzzy set I-VFS with the restriction that LFS ≤ UFS. To solve MCGDM problem a novel technique of bipolar fuzzy set have a large rang of [−1,1], to navigate uncertainty in particular solutions was proposed by Zang [6,7]. A number of aggregation techniques have been developed with bipolar fuzzy sets in mind. Some notable examples are the efficient city supply chain management through spherical fuzzy dynamic multistage decision analysis proposed by Riaz et al. [8]; the hybrid multi-criteria decision-making method with a bipolar fuzzy approach and its applications to economic condition analysis introduced by Jana et al. [9]; and the MABAC framework for logarithmic bipolar fuzzy MAGDM for supplier selection proposed by Jana et al. [10]. Atanassov [11] developed the intuitionistic fuzzy set (IFS) in 1986 to address the gap in the non-MF in FS. Aggregation operators (AOs) were developed inside the IFS framework by Zhao et al. [12] and Tan et al. [13,14]. When the value of non-membership is 0.7 and the value of membership is 0.8, intuitionistic fuzzy sets fail to meet their condition. In order to overcome this restriction, Yager [15] created the Pythagorean fuzzy set in 2013 and substituted the IFS constraint that the square of membership degree plus the square of non-membership degree must be between the [[16], [17]]. Numerous aggregation operators have been proposed in the context of Pythagorean fuzzy sets. The following are some notable examples: portfolio selection in a Pythagorean fuzzy environment with a GRA and FAHP framework by Paul et al. [18]; managing climate change and controlling global warming with carbon-dioxide storage assessment in geological media under a Pythagorean fuzzy VIKOR and DEMATEL framework by Paul et al. [19]; and basic uncertain information order geometric aggregation operators proposed by Jin et al. [20]. In 2016, Yager [21] expanded upon the idea of IFS and PFS by substituting the condition of IFS and PFS with the condition that the q power of membership degree plus the q power of non-membership degree must belong to [0,1]. Within the framework of q-ROFS put forward by Liu and Wang [22], Liu and Liu [23], Liu et al. [24], Jana et al. [25], Garg and Chen [26], and Jana et al. [27], several aggregation operators have been presented. Furthermore, other aggregation operations were proposed by Seikh et al. [28,29], and [30] in the context of q-ROFSs for MADM issues. A new cache reallocation-based page-level flash translation layer (CRFTL) was presented by Zhang et al. [31] along with its uses in smartphones; Chen et al. [32] suggested a non-negative temporal dimension preserved tensor completion model for the imputation of missing traffic data; and Lin et al. [33] suggested Linguistic q-ROFSs and their use in MADM issues. Lin et al. [34] use Pythagorean fuzzy sets to solve problems related to disease detection and cluster analysis, and they offer picture fuzzy interactional partitioned Heronian mean aggregation operators [35]. Ambiguity uncertainty in MADM problems are handled by MF and non-MF in the literature on classic fuzzy sets and its expansions. On the other hand, little information has been available regarding the level of uncertainty. It is significant to remember that current theories have particular restrictions within their fields. Smarandache [36] created a unique mathematical method in 1998 with tripled MF within the range of [0,3]. More efficiently, this tool deals with uncertainties by treating different kinds of scenarios: independent, somewhat independent, and totally dependent; on the other hand, fuzzy sets and all their generalized structures either wholly or partially neglect indeterminacy. Membership degrees (MD) and non-membership degrees (NMD) are normally evaluated using real values in all of the fuzzy structures that have been mentioned. But because MADM issues are so complex, Molodtsov [37] proposed soft sets in 1999 to overcome this constraint. These sets have now been merged with a variety of architectures (see Refs. [38,39,40,41], and [42]). Soft sets use real numbers. Q-rung orthopair fuzzy soft sets are a novel notion that was first described by Hussain et al. [43]. The degree of indeterminacy in object appraisal is frequently overlooked, despite the focus on MD and NMD in current discourse. In order to get over this restriction, this study suggest a brand-new hybrid strategy known as q-RONSSs, which combines neutrosophic sets and q-ROF $S_{f}S$. This method is unique in that it includes parameters that quantify the degrees of falsehood, indeterminacy, and truth.

### 1.1 Literature review

Neutrosophic sets (NS), in contrast to conventional fuzzy sets and its extensions, provide a more thorough knowledge of uncertainty. Including IDM improves the framework ability to handle uncertainty using indeterminate membership, which provide flexible and more effective approach to expressing complex information compared to traditional fuzzy sets. Neutrosophic set are well suited for different fields like AI, and data miming, effectively address the uncertainties in practical situations. Numerous experts have suggested various approaches for using Neutrosophic Sets (NSs). For instant, a serious of hybrid aggregation operators proposed by Garg and Nancy see Ref. [44]. A multi-distance interval-valued neutrosophic approach was proposed by Torkayesh et al. [45] to identify societal shortcomings in sustainable municipal garbage management. A multicriteria decision-making approach for 3D printing economic manufacturing utilizing a neutrosophic environment was presented by Liu and Tang [46]. In a neutrosophic set setting, Garg and Nancy [47] proposed a multi-criteria decision-making approach based on the prioritized Muirhead mean aggregation operator. Single-valued neutrosophic aggregation operations were proposed by Riaz et al. [48] for MCDM. Neutrosophic aggregation operators based on the soft max function were suggested by Garai et al. [49] and used in multi-attribute decision-making scenarios. Fuzzy set theory and its applications have advanced significantly in the last few years, examining a number of extensions and generalizations like q-SFRSs and q-ROPFSSs. In the context of q-ROPFSS, averaging aggregation operators was covered by Ali et al. [50]. Building on this, Ali et al. [51] addressed problems in green supply chain management by using the TOPSIS approach in a q-ROPF soft environment. q-spherical fuzzy rough sets were introduced by Azim et al. [52], who also showed how to use them in MADM scenarios. Furthermore, Azim et al. [53] investigated sine trigonometric q-SFR aggregation operators for group decision-making and digital transformation [54] and evaluated indoor positioning systems utilizing q-SFR TOPSIS analysis. Azim et al.'s [55] important addition was the application of the q-spherical fuzzy rough analytic hierarchy process for Industry 4.0 project prioritization. These sophisticated fuzzy set ideas offer strong frameworks to handle challenging situations involving decision-making in a variety of fields.

Upon analyzing the preceding discourse, it is evident that current investigations within the neutrosophic framework encounter challenges in effectively regulating the impact of an object's membership, indeterminacy membership, and non-membership within a set. In response to these limitations, we have introduced an innovative iteration of the q-ROFS, termed q−RONSSs. The primary objective of this novel construct is to precisely manage and govern the influence of MD, IMD, and NMD with attributes.

### 1.2 Research gap

In the literature, traditional fuzzy sets and their extension sets handle the uncertainties with MD and NMD, but there was no information about the indeterminacy degree also it is important to admit that the existing theories have specific limitations, which restricts decision-makers to evaluating alternatives within the domain. So, to tackle these limitations we proposed a more flexible and free structure by merging neutrosophic sets to q-rung orthopair fuzzy soft sets and proposed q-rung orthopair neutrosophic soft sets, support by tripled MF associated with attributes which provide the more flexible environment to the decision maker by relaxing the domain.

### 1.3 Motivations

Across various fields many researchers are interested in using neutrosophic sets and their way of combining information. In the previous structure of q-ROFSS, there is a lack of indeterminacy parameters and also some restrictions on their domain to regulate the influence of membership degree in the DM process. So for this indeed more flexible structure of neutrosophic set to handle the various types of uncertainties including independent, partially independent, and completely dependent, while q-fuzzy set and all of its generalized structure are entirely or partially ignored indeterminacy, and proposed q-RON soft sets which provide more reliable and flexible than PFS, SFS, and NS, because of condition 0≤(α)+(β)+(γ)≤1, $0\leq {\left(\alpha \right)}^{2}+{\left(\beta \right)}^{2}+{\left(\gamma \right)}^{2}\leq 1$ and 0≤(α)+(β)+(γ)≤3 but also $0\leq {\left(\alpha \right)}^{q}+{\left(\beta \right)}^{q}+{\left(\gamma \right)}^{q}\leq 3$.

### 1.4 Contributions

This research article presents several significant contributions:

i. To establish q-RONS sets.
ii. To define basic operational laws of q-rung orthopair neutrosophic soft sets.
iii. To develop aggregation operators based on the proposed structure such as $q-RONS_{f}WA$, $q-RONS_{f}OWA$, $q-RONS_{f}WG$ and $q-RONS_{f}OWG$ operators and to demon straight their fundamental properties.
iv. To address DM challenges, we adopted a novel technique of MCGDM within the framework of developed aggregation operators.
v. To address the reliability, of the proposed structure we performed a sensitivity analysis test.
vi. To demonstrate the comparative analysis, to show the authenticity of the proposed structure.
vii. To demonstrate characteristic analysis with existing theories, to express the superiority of the proposed structure.

**Fig. 1:**
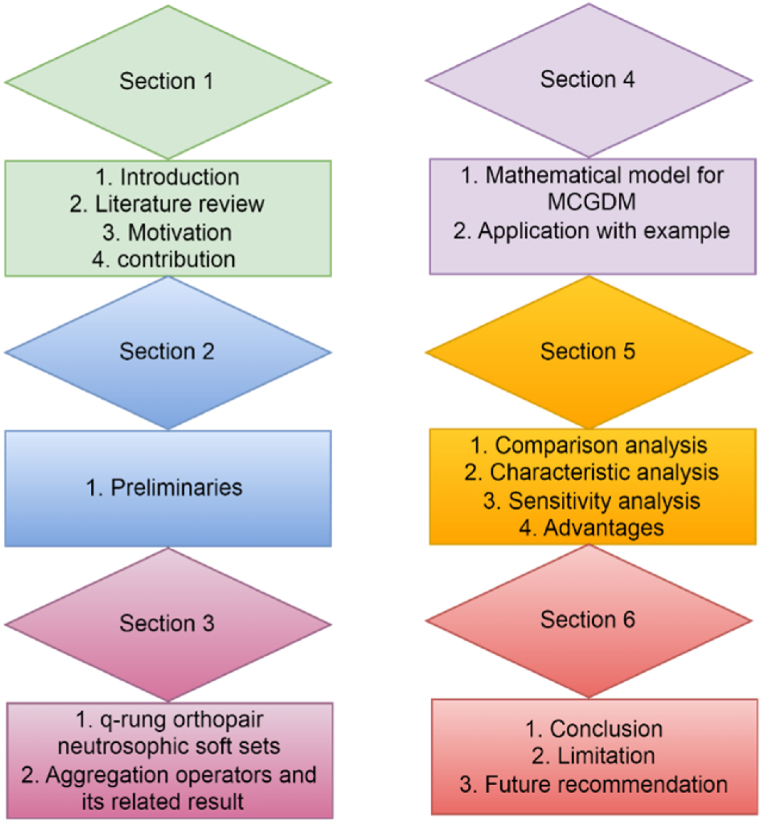
Expresses the detailed layout of the article.

This manuscript is organized as a Section 1 and serves as an introduction. Section 2, consists of basic preliminaries. Section 3, describes q-rung orthopair neutrosophic soft set and it's also developed averaging and geometric AOs based on the proposed structure and its related properties. Section 4, develops a mathematical model based on the proposed structure for solving the MCDM problem and considers a numerical example related to the personal selection problem of employment for a particular position in a real estate company for application. Section 5, the show shows the superiority and authenticity of the proposed model using a various analysis test with the existing model. In section 6, provide a conclusion. Fig. 1 expresses the detailed layout of the article.

## 2 Preliminaries

This section presents a number of mathematical ideas. It begins with a thorough analysis of soft sets, IFS, PFS, q-ROFS, $q-\text{ROF}S_{f}S$ and NS.

Definition 1 [37] A pair (F,₳) is defined as a soft set over the parameters € and the fixed set ₷, where ₳ ⊆ € and *F*: ₳ → P (₷).

Definition 2 [11] Given a given set S, the mathematical structure of an IFS ₳ can be written as follows:

(1) $$₳=\left\{\left\langle \mu ,L_{₳}\left(₰\right),G_{₳}\left(₰\right)\right\rangle :₰\in ₷,L_{₳}\left(₰\right),G_{₳}\left(₰\right)\in \left[0,1\right]\right\}$$

Where $L_{₳}\left(₰\right)$ and $G_{₳}\left(₰\right)$ express mem and n-mem, such that 0 ≤ $\left(L_{₳}\left(₰\right)\right)+\left(G_{₳}\left(₰\right)\right)\leq$ 1 and the score value S (₳)∈[−1,1] and accuracy function A (₳)∈[0,1] is express as

$$S\;\left(₳\right)=L_{₳}\left(₰\right)-G_{₳}\left(₰\right)$$

$$A\;\left(₳\right)=L_{₳}\left(₰\right)+G_{₳}\left(₰\right)\text{.}$$

Equation (1) depicts the essential composition of IFS.

Definition 3 [6]$P_{y}$ FS “₳” for a fixed set ₷ is defined as

(2) $$₳=\left\{\left\langle \mu ,L_{₳}\left(₰\right),G_{₳}\left(₰\right)\right\rangle :₰\in ₷,L_{₳}\left(₰\right),G_{₳}\left(₰\right)\in \left[0,1\right]\right\}$$

Where $L_{₳}\left(₰\right)$ and $G_{₳}\left(₰\right)$ express mem and n-mem, such that 0 ≤ ${\left(L_{₳}\left(₰\right)\right)}^{2}+{\left(G_{₳}\left(₰\right)\right)}^{2}\leq$ 1 and the score and accuracy value are express

$$S\;\left(₳\right)={\left(L_{₳}\left(₰\right)\right)}^{2}-{\left(G_{₳}\left(₰\right)\right)}^{2},S\;\left(₳\right)\in \left[-1,1\right]$$

$$A\;\left(₳\right)={\left(L_{₳}\left(₰\right)\right)}^{2}+{\left(G_{₳}\left(₰\right)\right)}^{2},A\;\left(₳\right)\in \left[0,1\right]\text{.}$$

Equation (2) depicts the essential composition of PyFS.

Definition 4 Given a given set ₷, the mathematical structure of a q-ROFS is represented by ₳ and is defined as follows:

(3) $$₳=\left\{\left\langle \mu ,L_{₳}\left(₰\right),G_{₳}\left(₰\right)\right\rangle :₰\in ₷,L_{₳}\left(₰\right),G_{₳}\left(₰\right)\in \left[0,1\right]\right\}$$

Where the membership and non-membership degrees are represented by $L_{₳}\left(₰\right)$ and $G_{₳}\left(₰\right)$, such that 0 ≤ ${\left(L_{₳}\left(₰\right)\right)}^{q}+{\left(G_{₳}\left(₰\right)\right)}^{q}\leq$ 1.

The accuracy as well as and score values are written as:

$$S\;\left(₳\right)\in \left[-1,1\right]={\left(L_{₳}\left(₰\right)\right)}^{q}-{\left(G_{₳}\left(₰\right)\right)}^{q}$$

$$A\;\left(₳\right)\in \left[0,1\right]={\left(L_{₳}\left(₰\right)\right)}^{q}+{\left(G_{₳}\left(₰\right)\right)}^{q}\text{.}$$

**Fig. 2:**
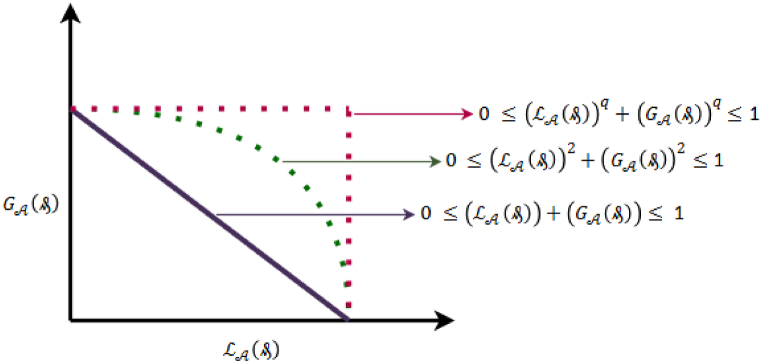
Express the analysis of distinctions among IF, PyF and q-ROF spaces.

Equation (3) represent the mathematical structure of an q-rung orthopair fuzzy set. Fig. 2 represent the difference between intuitionistic fuzzy set, Pythagorean fuzzy set and q-rung orthopair fuzzy set.

Definition 5 [18] A pair (₳,₵) is known as q-ROF $S_{f}$ S over the soft universe (₷,₵) and ₵⊆ €. Where ₳ is given by $₳:₵\to {q-\text{ROFS}}^{\left(\;₷\;\right)}$ and defined as Ϧ і ј

(4) $${₳}_{{Ϧ}_{ј}}\left(\mu_{і}\right)=\left\{\left\langle {₰}_{і},L_{ј}\left({₰}_{і}\right),G_{ј}\left({₰}_{і}\right)\right\rangle :{₰}_{і}\in ₷\;\right\}$$

Where $L_{ј}\left({₰}_{і}\right)$ and $G_{ј}\left({₰}_{і}\right)$ express mem and n-mem ${₰}_{і}\epsilon \;₷$ to a set ${₳}_{{Ϧ}_{ј}}\left({₰}_{і}\right)$, such that 0 ≤ ${\left(L_{ј}\left({₰}_{i}\right)\right)}^{q}+{\left(G_{ј}\left({₰}_{i}\right)\right)}^{q}\leq$ 1 (q≥1)

and the indeterminacy degree is expressed as $\pi_{{₳}_{{Ϧ}_{іј}}}$ = $\sqrt[{q}]{1-\left({\left(L_{ј}\left({₰}_{i}\right)\right)}^{q}+{\left(G_{ј}\left({₰}_{i}\right)\right)}^{q}\right)}$.

Equation (4) represent the mathematical structure of an q-rung orthopair fuzzy soft set.

Definition 6 [36] Let ₷ be a fixed set. A neutrosophic set ₳ is defined as:

(5) $$₳=\left\{\left\langle \mu ,L_{₳}\left(₰\right),G_{₳}\left(₰\right),{Ł}_{₳}\left(₰\right)\right\rangle :\mu \in ₷,L_{₳}\left(₰\right),G_{₳}\left(₰\right),{Ł}_{₳}\left(₰\right)\in \left[0,1\right]\;\right\}$$

Where $L_{₳}\left(₰\right)$, $G_{₳}\left(₰\right)$ and ${Ł}_{\tilde{N}}\left(₰\right)$ express truth, indeterminacy, and falsity degree, such that 0 ≤ $\left(L_{₳}\left(₰\right)\right)+\left(G_{₳}\left(₰\right)+\left({Ł}_{₳}\left(₰\right)\right)\right)\leq$ 3.

The score and accuracy values are written as:

$$S\;\left(₳\right)=\left(L_{₳}\left(₰\right)\right)-\left(G_{₳}\left(₰\right)\right)-\left({Ł}_{₳}\left(₰\right)\right)$$

$$A\;\left(₳\right)=\left(L_{₳}\left(₰\right)\right)+\left(G_{₳}\left(₰\right)\right)+\left({Ł}_{₳}\left(₰\right)\right)\text{.}$$

Equation (5) depicts the essential composition of NS.

Definition 7 Using the t-norm Ҭ, t-conorm S, Einstein's operations are as follows:

(6) $${Ҭ}_{Ḛ}\left(X,Y\right)=\frac{X+Y}{1+\left(1-X\right)\left(1-Y\right)}$$

(7) $$S_{Ḛ}\left(X,Y\right)=\frac{X+Y}{1+XY}$$

Equation (6) and Equation (7) represent the t-norm and t-conorm of Einstein operations.

## 3 q-rung orthopair neutrosophic soft set

This section is devoted to a thorough investigation of the operational laws guiding the proposed structure of q-RONSS. It also includes an explanation of the basic features of aggregation operators, including $q-RONS_{f}WA$, $q-RONS_{f}OWA$, $q-RONS_{f}WG$ and $q-RONS_{f}OWG$ operators, as well as their construction and analysis.

Definition 8 Let (₷,₵) be a soft universe and ₵⊆ €. And the pair (₳,₵) over ₷, where ₳ is a function given by $₳:₵\to {q-RONFS}^{\left(₷\right)}$ is express q-RON $S_{f}$ S which is defined as

(8) $${₳}_{{Ϧ}_{j}}\left({₰}_{i}\right)=\left\{\left\langle {₰}_{i},{Ʈ}_{j}\left({₰}_{i}\right),{ƚ}_{j}\left({₰}_{i}\right),{Ƒ}_{j}\left({₰}_{i}\right)\;\right\rangle :{₰}_{i}\in ₷\;\text{and}\;q\geq 1\right\}$$

Where ${Ʈ}_{j}\left({₰}_{i}\right),{ƚ}_{j}\left({₰}_{i}\right),{Ƒ}_{j}\left({₰}_{i}\right)\in \left[0,1\right]$ signify the degree of truth, indeterminacy, and falsity of memberships of ${₰}_{i}\in ₷$ to a set ${₳}_{{Ϧ}_{j}}\left({₰}_{i}\right)$, and respectively

$$0\leq {\left({Ʈ}_{j}\left({₰}_{i}\right)\right)}^{q}+{\left({ƚ}_{j}\left({₰}_{i}\right)\right)}^{q}+{\left({Ƒ}_{j}\left({₰}_{i}\right)\right)}^{q}\leq 3\;\left(q\geq 1\right)$$

For convenience ${₳}_{{Ϧ}_{j}}\left({₰}_{i}\right)$ = ${\left\langle {₰}_{i},{Ʈ}_{j}\left({₰}_{i}\right),{ƚ}_{j}\left({₰}_{i}\right),{Ƒ}_{j}\left({₰}_{i}\right)\right\rangle }_{q}$ called q-RON $S_{f}$ S. And the degree of hesitancy is defined as

$$\pi_{{₳}_{{Ϧ}_{ij}}}=\sqrt[{q}]{1-\left({\left({Ʈ}_{j}\left({₰}_{i}\right)\right)}^{q}+{\left({ƚ}_{j}\left({₰}_{i}\right)\right)}^{q}+{\left({Ƒ}_{j}\left({₰}_{i}\right)\right)}^{q}\right)}\text{.}$$

Equation (8) represent the mathematical structure of an q-rung orthopair neutrosophic soft set.

Definition 9 Consider be any three q-RONF soft numbers ₳=(Ʈ,ƚ,Ƒ) and ${₳}_{{Ϧ}_{1j}}$ = $\left({Ʈ}_{1j},{ƚ}_{1j},{Ƒ}_{1j}\right)$ for j = 1,2 and λ ≻ 0., the operations are defined as follows

$\left(i\right).\;{₳}^{C}=\left(Ƒ,ƚ,Ʈ\right)$.
$\left(\text{ii}\right).\;{₳}_{{Ϧ}_{11}}\leq {₳}_{{Ϧ}_{12}}\;\text{if}\;\text{and}\;\text{only}\;\text{if}\;\left({Ʈ}_{11}\leq {Ʈ}_{12},{ƚ}_{11}\geq {ƚ}_{12},{Ƒ}_{11}\geq {Ƒ}_{12}\right)$.
$\left(\text{iii}\right).\;{₳}_{{Ϧ}_{11}}\cup {₳}_{{Ϧ}_{12}}=\left(\max\left({Ʈ}_{11},{Ʈ}_{12}\right),\min\left({ƚ}_{11},{ƚ}_{12}\right),\min\left(\;{Ƒ}_{11},{Ƒ}_{12}\right)\right)$.
$\left(\text{iv}\right).\;{₳}_{{Ϧ}_{11}}\cap {₳}_{{Ϧ}_{12}}=\left(\min\left({Ʈ}_{11},{Ʈ}_{12}\right),\max\left({ƚ}_{11},{ƚ}_{12}\right),\max\left(\;{Ƒ}_{11},{Ƒ}_{12}\right)\right)$.
$\left(v\right).\;{₳}_{{Ϧ}_{11}}⊕{₳}_{{Ϧ}_{12}}=\left(\sqrt[{q}]{{\left({Ʈ}_{11}\right)}^{q}+{\left({Ʈ}_{12}\right)}^{q}-{\left({Ʈ}_{11}\right)}^{q}{\left({Ʈ}_{12}\right)}^{q}},{ƚ}_{11}{ƚ}_{12},{Ƒ}_{11}{Ƒ}_{12}\right)$.
$\left(\text{vi}\right).\;{₳}_{{Ϧ}_{11}}⊗{₳}_{{Ϧ}_{12}}=\left({Ʈ}_{11}{Ʈ}_{12},\sqrt[{q}]{{\left({ƚ}_{11}\right)}^{q}+{\left({ƚ}_{12}\right)}^{q}-{\left({ƚ}_{11}\right)}^{q}{\left({ƚ}_{12}\right)}^{q}},\sqrt[{q}]{{\left({Ƒ}_{11}\right)}^{q}+{\left({Ƒ}_{12}\right)}^{q}-{\left({Ƒ}_{11}\right)}^{q}{\left({Ƒ}_{12}\right)}^{q}}\right)$.
$\left(\text{vii}\right).\;\lambda ₳=\left(\sqrt[{q}]{1-{\left(1-{\left(Ʈ\right)}^{q}\right)}^{\lambda }},{\left(ƚ\right)}^{\lambda },{\left(Ƒ\right)}^{\lambda }\right)$.
$\left(\text{viii}\right).\;{₳}^{\lambda }=\left(\begin{array}{c} {\left(Ʈ\right)}^{\lambda },\\ \sqrt[{q}]{1-{\left(1-{\left(ƚ\right)}^{q}\right)}^{\lambda }},\\ \sqrt[{q}]{1-{\left(1-{\left(Ƒ\right)}^{q}\right)}^{\lambda }} \end{array}\right)\text{.}$.

Definition 10 A score function of q-RONF $S_{f}N_{s}\;{₳}_{{Ϧ}_{ij}}$ = $\left({Ʈ}_{ij},{ƚ}_{ij},{Ƒ}_{ij}\right)$ can be defined as

(9) $$S\;\left({₳}_{{Ϧ}_{ij}}\right)=S\left({₳}_{{Ϧ}_{ij}}\right)\in \left[-3,3\right]={\left({Ʈ}_{ij}\right)}^{q}\;-\;{\left({ƚ}_{ij}\right)}^{q}-{\left({Ƒ}_{ij}\right)}^{q}+\left(\frac{e^{{\left({Ʈ}_{ij}\right)}^{q}-{\left({ƚ}_{ij}\right)}^{q}-{\left({Ƒ}_{ij}\right)}^{q}}}{e^{{\left({Ʈ}_{ij}\right)}^{q}-{\left({ƚ}_{ij}\right)}^{q}-{\left({Ƒ}_{ij}\right)}^{q}}+1}-\frac{1}{2}\right)\pi_{{₳}_{{Ϧ}_{ij}}}^{q},\left(q\geq 1\;\right)$$

Equation (9) represent the score function of q-rung orthopair neutrosophic soft set.

Definition 11 The accuracy function for q-RONF $S_{t}N_{s}$ ${₳}_{{Ϧ}_{ij}}=\left({Ʈ}_{ij},{ƚ}_{ij},{Ƒ}_{ij}\right)$ is defined as

$$Acc\left({₳}_{{Ϧ}_{ij}}\right)={\left({Ʈ}_{ij}\right)}^{q}+{\left({ƚ}_{ij}\right)}^{q}+{\left({Ƒ}_{ij}\right)}^{q}$$

Definition 12 Let ${₳}_{{Ϧ}_{11}}$ = $\left({Ʈ}_{11},{ƚ}_{11},{Ƒ}_{11}\right)$ and ${₳}_{{Ϧ}_{12}}$ = $\left({Ʈ}_{12},{ƚ}_{12},{Ƒ}_{12}\right)$ be two $q-\text{RONF}S_{f}N_{s}$. Then

I. S $\left({₳}_{{Ϧ}_{11}}\right)\;<$ S $\left({₳}_{{Ϧ}_{12}}\right)$, ${₳}_{{Ϧ}_{11}}<{₳}_{{Ϧ}_{12}}$
II. S $\left({₳}_{{Ϧ}_{11}}\right)\;>$ S $\left({₳}_{{Ϧ}_{12}}\right)$, ${₳}_{{Ϧ}_{11}}>{₳}_{{Ϧ}_{12}}$
III. S $\left({₳}_{{Ϧ}_{11}}\right)=$ S $\left({₳}_{{Ϧ}_{12}}\right)$, then
  a. $Acc\left({₳}_{{Ϧ}_{11}}\right)\;<$$Acc\left({₳}_{{Ϧ}_{12}}\right)\text{then}$${₳}_{{Ϧ}_{11}}<\;{₳}_{{Ϧ}_{12}}$
  b. $Acc\left({₳}_{{Ϧ}_{11}}\right)\;>$$Acc\left({₳}_{{Ϧ}_{12}}\right)\text{then}$${₳}_{{Ϧ}_{11}}>\;{₳}_{{Ϧ}_{12}}$
  c. $Acc\left({₳}_{{Ϧ}_{11}}\right)=$$Acc\left({₳}_{{Ϧ}_{12}}\right)\text{then}$${₳}_{{Ϧ}_{11}}={₳}_{{Ϧ}_{12}}$

Example 1 Assume a decision-maker who is in the process of selecting a smartwatch from a market offering four alternatives Ύ = {$O_{1}$, $O_{2}$, $O_{3}$, $O_{4}$} associated with specific parameters €= {${ӿ}_{1}$, ${ӿ}_{2}$, ${ӿ}_{3},{ӿ}_{4}$}

${ӿ}_{1}=$ Fitness Features

$x_{2}=\text{Assess}\;\text{Notifications}$

${ӿ}_{3}=$ Smart Compass

${ӿ}_{4}=$ Play Music

When we assess the various options using the assigned rating values, we obtain the outcome present in "Table 1", which is expressed in the form of $q-\text{RONF}S_{f}N_{s}$.

Theorem 1 Let ${₳}_{{Ϧ}_{\text{ij}}}=\left({Ʈ}_{\text{ij}},{ƚ}_{\text{ij}},{Ƒ}_{\text{ij}}\right)$ and ₳=(Ʈ,ƚ,Ƒ) be any two q-RONF $S_{f}N_{s}$ and λ, $\lambda_{1}$, $\lambda_{2}$ ≻0, having the properties.

i ${₳}_{{Ϧ}_{11}}⊕{₳}_{{Ϧ}_{12}}={₳}_{{Ϧ}_{12}}⊕{₳}_{{Ϧ}_{11}}$
ii ${₳}_{{Ϧ}_{11}}⊗{₳}_{{Ϧ}_{12}}=$${₳}_{{Ϧ}_{12}}⊗{₳}_{{Ϧ}_{11}}$
iii $\lambda \left({₳}_{{\ddot{Ϧ}}_{11}}⊕{₳}_{{Ϧ}_{12}}\right)=\lambda {₳}_{{Ϧ}_{11}}⊕\lambda {₳}_{{Ϧ}_{12}}$
iv $\left(\lambda_{1}⊕\lambda_{2}\right)₳=\lambda_{1}₳⊕\lambda_{2}₳$
v ${₳}^{\left(\lambda_{1}⊕\lambda_{2}\right)}={₳}^{\lambda_{1}}⊗{₳}^{\lambda_{2}}$
vi ${₳}_{{Ϧ}_{11}}^{\lambda }⊗{₳}_{{Ϧ}_{12}}^{\lambda }={\left({₳}_{{Ϧ}_{11}}⊕{₳}_{{Ϧ}_{12}}\right)}^{\lambda }$.

**Proof**: Straightforward.

**Table 1:** Represent $q-\text{RON}S_{f}N_{s}$${₳}_{{Ϧ}_{\text{ij}}}$ = $\left({Ʈ}_{\text{ij}},{ƚ}_{\text{ij}},{Ƒ}_{\text{ij}}\right)$ for q ≥ 3.

Ύ	Fitness Features	Assess Notifications	Smart Compass	Play Music
$O_{1}$	(.8,0.5,0.6)	(0.6,0.2,0.4)	(0.4,0.1,0.4)	(0.6,0.4,0.2)
$O_{2}$	(.6,0.3,0.2)	(0.4,0.1,0.3)	(0.7,0.3,0.4)	(0.5,0.3,0.4)
$O_{3}$	(.5,0.2,0.1)	(0.7,0.2,0.5)	(0.3,0.1,0.6)	(0.5,0.5,0.1)
$O_{4}$	(.4,0.3,0.2)	(0.8,0.1,0.1)	(0.3,0.3,0.4)	(0.7,0.1,0.2)

### 3.1 Weighted averaging operator using q-RONS set

Let ${₳}_{{Ϧ}_{ij}}$ = $\left({Ʈ}_{\text{ij}},{ƚ}_{\text{ij}},{Ƒ}_{\text{ij}}\right)$ (i=1,2,3,…,n)(j=1,2,3,…,m) be a family of q-RONF $S_{f}N_{s}$ having the weight vector of the experts $x_{i}$ is $\omega_{i}$ and for the parameters $e_{j}$ is $\upsilon_{j}$ such that $\omega_{i}$, $\upsilon_{j}\in \left[0,1\right]$ with the constraints that $\sum_{i=1}^{n}\omega_{i}=1\;\text{and}\;\sum_{j=1}^{m}\upsilon_{j}=1$ respectively.

Then q-RON $S_{f}$ WA operator is defined by the mapping q-RON $S_{f}$ WA: ${Đ}^{n}⟶$ Đ, where Đ represents the collection of q-RONF $S_{f}N_{s}$.

$$q-\text{RON}\;S_{f}\;\text{WA}\;\left({₳}_{{Ϧ}_{11}},{₳}_{{Ϧ}_{12}},\ldots .,{₳}_{{Ϧ}_{nm}}\right)={⊕}_{j=1}^{m}\upsilon_{i}\left({⊕}_{i=1}^{n}\omega_{i}{₳}_{{Ϧ}_{ij}}\right)$$

Theorem 2 *Let*${₳}_{{Ϧ}_{ij}}$*=*$\left({Ʈ}_{\text{ij}},{ƚ}_{\text{ij}},{Ƒ}_{\text{ij}}\right)$*be a collection of q-RONF*$S_{f}N_{s}$. *Then*, *the aggregation result for the q- q-RON*$S_{f}$*WA operator is defined as*:

$$q-\text{RON}\;S_{f}\;\text{WA}\;\left({₳}_{{Ϧ}_{11}},{₳}_{{Ϧ}_{12}},\ldots .,{₳}_{{Ϧ}_{nm}}\right)={⊕}_{j=1}^{m}\upsilon_{i}\left({⊕}_{i=1}^{n}\omega_{i}{₳}_{{Ϧ}_{ij}}\right)$$

$$=\left(\sqrt[{q}]{1-\prod_{j=1}^{m}{\left(\prod_{i=1}^{n}{\left(1-{Ʈ}_{ij}^{q}\right)}^{\omega_{i}}\right)}^{\upsilon_{j}}},\Pi_{j=1}^{m}{\left(\Pi_{i=1}^{n}{ƚ}_{ij}^{\omega_{i}}\right)}^{\upsilon_{j}},\Pi_{j=1}^{m}{\left(\Pi_{i=1}^{n}{Ƒ}_{ij}^{\omega_{i}}\right)}^{\upsilon_{j}}\right)$$

(10) $$q-\text{RON}\;S_{f}\;\text{WA}\;\left({₳}_{{Ϧ}_{11}},{₳}_{{Ϧ}_{12}},\ldots .,{₳}_{{Ϧ}_{nm}}\right)=\left(\begin{array}{c} \sqrt[{q}]{1-\prod_{j=1}^{m}{\left(\prod_{i=1}^{n}{\left(1-{Ʈ}_{ij}^{q}\right)}^{\omega_{i}}\right)}^{\upsilon_{j}}},\\ \Pi_{j=1}^{m}{\left(\Pi_{i=1}^{n}{ƚ}_{ij}^{\omega_{i}}\right)}^{\upsilon_{j}},\\ \Pi_{j=1}^{m}{\left(\Pi_{i=1}^{n}{Ƒ}_{ij}^{\omega_{i}}\right)}^{\upsilon_{j}} \end{array}\right)$$

The q-rung orthopair neutrosophic soft weighted averaging operator is represented by equation (10).

Where $\omega_{i}$ and $\upsilon_{j}$ are the WV for expert x_i_ and parameters e_j_, respectively, with the condition $\sum_{i=1}^{n}\omega_{i}=1$ and $\sum_{j=1}^{m}\upsilon_{j}=1\text{.}$.

**Proof**: By mathematical induction, we solve this result. We have,

$${₳}_{{Ϧ}_{11}}⊕{₳}_{{Ϧ}_{12}}=\left(\sqrt[{q}]{{\left({Ʈ}_{11}\right)}^{q}+{\left({Ʈ}_{12}\right)}^{q}-{\left({Ʈ}_{11}\right)}^{q}{\left({Ʈ}_{12}\right)}^{q}},{\;ƚ}_{11}{ƚ}_{12},{Ƒ}_{11}{Ƒ}_{12}\right)\;\text{and}\;\lambda ₳=\left(\left(\sqrt[{q}]{1-{\left[1-{Ʈ}^{q}\right]}^{\lambda }},{ƚ}^{\lambda },{Ƒ}^{\lambda }\right)\right)\text{.}$$

Step 1 Initially, examine the case when n is equal to 2 and m is equal to 2.

$$q‐\text{RON}\;S_{f}\;\text{WA}\;\left({₳}_{{Ϧ}_{11}},{₳}_{{Ϧ}_{12}},\ldots .,{₳}_{{Ϧ}_{nm}}\right)={⊕}_{j=1}^{2}\upsilon_{i}\left({⊕}_{i=1}^{2}\omega_{i}{₳}_{{Ϧ}_{ij}}\right)$$

$$={\;\upsilon }_{1}\left({⊕}_{i=1}^{2}\omega_{i}{₳}_{{Ϧ}_{11}}\right)⊕\upsilon_{2}\left({⊕}_{i=1}^{2}\omega_{i}{₳}_{{Ϧ}_{12}}\right)$$

$$=\upsilon_{1}\left(\omega_{1}{₳}_{{Ϧ}_{11}}⊕\omega_{2}{₳}_{{Ϧ}_{21}}\right)⊕\upsilon_{2}\left(\omega_{1}{₳}_{{Ϧ}_{12}}⊕\omega_{2}{₳}_{{Ϧ}_{22}}\right)$$

$$∴\omega_{1}{₳}_{{Ϧ}_{11}}=\left(\sqrt[{q}]{1-{\left(1-{Ʈ}_{11}^{q}\right)}^{\omega_{1}}},{ƚ}_{11}^{\omega_{1}},{Ƒ}_{11}^{\omega_{1}}\right)\;\text{and}\;\omega_{2}{₳}_{{Ϧ}_{21}}=\left(\sqrt[{q}]{1-{\left(1-{Ʈ}_{21}^{q}\right)}^{\omega_{2}}},{ƚ}_{21}^{\omega_{2}},{Ƒ}_{21}^{\omega_{2}}\right)$$

$$={\;\upsilon }_{1}\left\{\left(\sqrt[{q}]{1-{\left(1-{Ʈ}_{11}^{q}\right)}^{\omega_{1}}},{ƚ}_{11}^{\omega_{1}},{Ƒ}_{11}^{\omega_{1}}\right)⊕\left(\sqrt[{q}]{1-{\left(1-{Ʈ}_{21}^{q}\right)}^{\omega_{2}}},{ƚ}_{21}^{\omega_{2}},{Ƒ}_{21}^{\omega_{2}}\right)\right\}⊕\upsilon_{2}\left\{\left(\sqrt[{q}]{1-{\left(1-{Ʈ}_{12}^{q}\right)}^{\omega_{1}}},{ƚ}_{12}^{\omega_{1}},{Ƒ}_{12}^{\omega_{1}}\right)⊕\left(\sqrt[{q}]{1-{\left(1-{Ʈ}_{22}^{q}\right)}^{\omega_{2}}},{ƚ}_{22}^{\omega_{2}},{Ƒ}_{22}^{\omega_{2}}\right)\right\}$$

$$={\;\upsilon }_{1}\left(\sqrt[{q}]{1-{\Pi_{i=1}^{2}\left(1-{Ʈ}_{i1}^{q}\right)}^{\omega_{i}}},{\Pi_{i=1}^{2}ƚ}_{i1}^{\omega_{i}},\Pi_{i=1}^{2}{Ƒ}_{i1}^{\omega_{i}}\right)⊕\upsilon_{2}\left(\sqrt[{q}]{1-{\Pi_{i=1}^{2}\left(1-{Ʈ}_{i2}^{q}\right)}^{\omega_{i}}},{\Pi_{i=1}^{2}ƚ}_{i2}^{\omega_{i}},\Pi_{i=1}^{2}{Ƒ}_{i2}^{\omega_{i}}\right)$$

$$=\upsilon_{1}\left(\sqrt[{q}]{1-{\left({\Pi_{i=1}^{2}\left(1-{Ʈ}_{i1}^{q}\right)}^{\omega_{i}}\right)}^{\upsilon_{1}}},{\left({\Pi_{i=1}^{2}ƚ}_{i1}^{\omega_{i}}\right)}^{\upsilon_{1}},{\left(\Pi_{i=1}^{2}{Ƒ}_{i1}^{\omega_{i}}\right)}^{\upsilon_{1}}\right)⊕\upsilon_{2}\left(\sqrt[{q}]{1-{\left({\Pi_{i=1}^{2}\left(1-{Ʈ}_{i2}^{q}\right)}^{\omega_{i}}\right)}^{\upsilon_{2}}},{\left({\Pi_{i=1}^{2}ƚ}_{i2}^{\omega_{i}}\right)}^{\upsilon_{2}},{\left(\Pi_{i=1}^{2}{Ƒ}_{i2}^{\omega_{i}}\right)}^{\upsilon_{2}}\right)$$

$$=\left(\sqrt[{q}]{1-\prod_{j=1}^{2}{\left(\prod_{i=1}^{2}{\left(1-{Ʈ}_{ij}^{q}\right)}^{\omega_{i}}\right)}^{\upsilon_{j}}},\Pi_{j=1}^{2}{\left(\Pi_{i=1}^{2}{ƚ}_{ij}^{\omega_{i}}\right)}^{\upsilon_{j}},\Pi_{j=1}^{2}{\left(\Pi_{i=1}^{2}{Ƒ}_{ij}^{\omega_{i}}\right)}^{\upsilon_{j}}\right)$$

Step 2 In second step, we consider for n = $\kappa_{1}$ and m = $\kappa_{2}$.

$$q-\text{RON}\;S_{f}\;\text{WA}\;\left({₳}_{{Ϧ}_{11}},{₳}_{{Ϧ}_{12}},\ldots .,{₳}_{{Ϧ}_{\kappa_{1}\kappa_{2}}}\right)={⊕}_{j=1}^{\kappa_{2}.}\upsilon_{i}\left({⊕}_{i=1}^{\kappa_{1}}\omega_{i}{₳}_{{Ϧ}_{\text{ij}}}\right)=\left(\sqrt[{q}]{1-\prod_{j=1}^{\kappa_{2}}{\left(\prod_{i=1}^{\kappa_{1}}{\left(1-{Ʈ}_{ij}^{q}\right)}^{\omega_{i}}\right)}^{\upsilon_{j}}},\Pi_{j=1}^{\kappa_{2}}{\left(\Pi_{i=1}^{\kappa_{1}}{ƚ}_{ij}^{\omega_{i}}\right)}^{\upsilon_{j}},\Pi_{j=1}^{\kappa_{2}}{\left(\Pi_{i=1}^{\kappa_{1}}{Ƒ}_{ij}^{\omega_{i}}\right)}^{\upsilon_{j}}\right)$$

Step 3 Finally, for n = $\kappa_{1}+1$ and m = $\kappa_{2}+1$.

$$q‐\text{RON}\;S_{f}\;\text{WA}\;\left({₳}_{{Ϧ}_{11}},{₳}_{{Ϧ}_{12}},\ldots .,{₳}_{{Ϧ}_{\kappa_{1}+1\;\kappa_{2}+1}}\right)=\left\{{⊕}_{j=1}^{\kappa_{2}}\upsilon_{j}\left({⊕}_{i=1}^{\kappa_{1}}\omega_{i}{₳}_{{Ϧ}_{\text{ij}}}\right)\right\}⊕\upsilon_{\left(\kappa_{1}+1\right)\;}\left(\omega_{\kappa_{2}+1}{₳}_{{\ddot{e}}_{\left(\kappa_{1}+1\right)\left(\kappa_{2}+1\right)}}\right)$$

$$=\left(\begin{array}{c} \sqrt[{q}]{1-\prod_{j=1}^{\kappa_{2}}{\left(\prod_{i=1}^{\kappa_{1}}{\left(1-{Ʈ}_{ij}^{q}\right)}^{\omega_{i}}\right)}^{\upsilon_{j}}},\\ \Pi_{j=1}^{\kappa_{2}}{\left(\Pi_{i=1}^{\kappa_{1}}{ƚ}_{ij}^{\omega_{i}}\right)}^{\upsilon_{i}},\Pi_{j=1}^{\kappa_{2}}{\left(\Pi_{i=1}^{\kappa_{1}}{Ƒ}_{ij}^{\omega_{i}}\right)}^{\upsilon_{j}} \end{array}\right)⊕\upsilon_{\left(\kappa_{1}+1\right)\;}\left(\omega_{\kappa_{2}+1}{₳}_{{Ϧ}_{\left(\kappa_{1}+1\right)\left(\kappa_{2}+1\right)}}\right)$$

$$=\left(\sqrt[{q}]{1-\prod_{j=1}^{\left(\kappa_{2}+1\right)}{\left(\prod_{i=1}^{\left(\kappa_{1}+1\right)}{\left(1-{Ʈ}_{ij}^{q}\right)}^{\omega_{i}}\right)}^{\upsilon_{j}}},\Pi_{j=1}^{\left(\kappa_{2}+1\right)}{\left(\Pi_{i=1}^{\left(\kappa_{1}+1\right)}{ƚ}_{ij}^{\omega_{i}}\right)}^{\upsilon_{j}},\Pi_{j=1}^{\left(\kappa_{2}+1\right)}{\left(\Pi_{i=1}^{\left(\kappa_{1}+1\right)}{Ƒ}_{ij}^{\omega_{i}}\right)}^{\upsilon_{j}}\right)$$

Now, in the cases where n = $\kappa_{1}+1$ and m = $\kappa_{2}+1$, where ∀m,n≥1, Equation (10) holds true. Given that ${₳}_{{Ϧ}_{\text{ij}}}$ = $\left({Ʈ}_{\text{ij}},{ƚ}_{\text{ij}},{Ƒ}_{\text{ij}}\right)$ denote a collection of q-RON $S_{f}N_{s}$, where 0 ≤ ${Ʈ}_{\text{ij}},{ƚ}_{\text{ij}},{Ƒ}_{\text{ij}}\leq 3$ satisfies the condition $0\leq {Ʈ}_{\text{ij}}+{ƚ}_{\text{ij}}+{Ƒ}_{\text{ij}}\leq 3$ for expert x_i_ and parameters e_j_, with weights $\omega_{i}$ such that $\sum_{i=1}^{n}\omega_{i}=1$ and $\upsilon_{j}$ such that $\sum_{j=1}^{m}\upsilon_{j}=1$.

$$0\leq {Ʈ}_{\text{ij}}\leq 3$$

$$\Rightarrow 0\leq {1-Ʈ}_{\text{ij}}\leq 3$$

$$\Rightarrow \;0\leq {\left(1-{Ʈ}_{ij}^{q}\right)}^{\omega_{i}}\leq 3$$

$$\Rightarrow \;0\leq {\Pi_{i=1}^{n}\left(1-{Ʈ}_{ij}^{q}\right)}^{\omega_{i}}\leq 3$$

$$\Rightarrow \;0\leq \Pi_{j=1}^{m}{\left({\Pi_{i=1}^{n}\left(1-{Ʈ}_{ij}^{q}\right)}^{\omega_{i}}\right)}^{\upsilon_{j}}\leq 3$$

$$\Rightarrow \;0\leq \sqrt[{q}]{\Pi_{j=1}^{m}{\left({\Pi_{i=1}^{n}\left(1-{Ʈ}_{ij}^{q}\right)}^{\omega_{i}}\right)}^{j}}\leq 3\text{.}$$

$$\text{Now},\text{for}\;0\leq {ƚ}_{\text{ij}}\leq 3$$

$$\Rightarrow 0\leq {\;\Pi }_{i=1}^{n}\;{ƚ}_{ij}^{\omega_{i}}\leq 3$$

$$\Rightarrow \;0\leq \Pi_{j=1}^{m}{\left(\Pi_{i=1}^{n}\;{ƚ}_{ij}^{\omega_{i}}\right)}^{\upsilon_{j}}\leq 3$$

and finally

$$0\leq {Ƒ}_{\text{ij}}\leq 3$$

$$\Rightarrow 0\leq {\;\Pi }_{i=1}^{n}\;{Ƒ}_{ij}^{\omega_{i}}\leq 3$$

$$\Rightarrow \;0\leq \Pi_{j=1}^{m}{\left(\Pi_{i=1}^{n}\;{Ƒ}_{ij}^{\omega_{i}}\right)}^{\upsilon_{j}}\leq 3\text{.}$$

$$\text{As},{0\leq Ʈ}_{ij}^{q}+{\;ƚ}_{ij}^{q}+{\;Ƒ}_{ij}^{q}\leq 3\;\Rightarrow \;{\;Ʈ}_{ij}^{q}+{\;Ƒ}_{ij}^{q}\leq 1-{\;ƚ}_{ij}^{q}$$

$$\Rightarrow \;\Pi_{i=1}^{n}{\left({\;Ʈ}_{ij}^{q}\right)}^{\omega_{i}}+{\Pi_{i=1}^{n}\left({\;Ƒ}_{ij}^{q}\right)}^{\omega_{i}}\leq {\Pi_{i=1}^{n}\left(1-{\;ƚ}_{ij}^{q}\right)}^{\omega_{i}}$$

$$\Rightarrow \Pi_{j=1}^{m}{\left(\Pi_{i=1}^{n}{\left({\;Ʈ}_{ij}^{q}\right)}^{\omega_{i}}\right)}^{\upsilon_{j}}+\Pi_{j=1}^{m}{\left({\Pi_{i=1}^{n}\left({\;Ƒ}_{ij}^{q}\right)}^{\omega_{i}}\right)}^{\upsilon_{j}}\leq \Pi_{j=1}^{m}{\left({\Pi_{i=1}^{n}\left(1-{\;ƚ}_{ij}^{q}\right)}^{\omega_{i}}\right)}^{\upsilon_{j}}$$

Now, we have

$$0\leq {\left\{\sqrt[{q}]{1-\prod_{j=1}^{m}{\left(\prod_{i=1}^{n}{\left(1-{ƚ}_{ij}^{q}\right)}^{\omega_{i}}\right)}^{\upsilon_{j}}}\right\}}^{q}+{\left\{\Pi_{j=1}^{m}{\left(\Pi_{i=1}^{n}{Ʈ}_{ij}^{\omega_{i}}\right)}^{\upsilon_{j}}\right\}}^{q}+{\left\{\Pi_{j=1}^{m}{\left(\Pi_{i=1}^{n}{Ƒ}_{ij}^{\omega_{i}}\right)}^{\upsilon_{j}}\right\}}^{q}\text{,}$$

$$0\leq 1-\prod_{j=1}^{m}{\left(\prod_{i=1}^{n}{\left(1-{ƚ}_{ij}^{q}\right)}^{\omega_{i}}\right)}^{\upsilon_{j}}+\Pi_{j=1}^{m}{\left(\Pi_{i=1}^{n}{Ʈ}_{ij}^{\omega_{i}}\right)}^{\upsilon_{j}}+\Pi_{j=1}^{m}{\left(\Pi_{i=1}^{n}{Ł}_{ij}^{\omega_{i}}\right)}^{\upsilon_{j}}=3$$

Therefore,

$$0\leq {\left\{\sqrt[{q}]{1-\prod_{j=1}^{m}{\left(\prod_{i=1}^{n}{\left(1-{ƚ}_{ij}^{q}\right)}^{\omega_{i}}\right)}^{\upsilon_{j}}}\right\}}^{q}+{\left\{\Pi_{j=1}^{m}{\left(\Pi_{i=1}^{n}{Ʈ}_{ij}^{\omega_{i}}\right)}^{\upsilon_{j}}\right\}}^{q}+{\left\{\Pi_{j=1}^{m}{\left(\Pi_{i=1}^{n}{Ƒ}_{ij}^{\omega_{i}}\right)}^{\upsilon_{j}}\right\}}^{q}\leq 3\text{.}$$

Hence, we proved the required result.

Example 2 Miss Marjan is charged with choosing the best life mate from among four people in the domain ₷ = {$h_{1}$, $h_{2}$, $h_{3}$, $h_{4}$}, where $h_{1}$ = Ali, $h_{2}$ = Shoaib, $h_{3}$ = Majeed and $h_{4}$ = Ahmad. This selection is based on the parameters € = {$g_{1}$, $g_{2}$, $g_{3},g_{4}$}, where $g_{1}$ = Trustworthy, $g_{2}$ = Loyal, $g_{3}$ = Dependable and $g_{4}$ = Compromising. Weights ω = {0.20,0.25,0.27,0.28} and υ = {0.5,0.16,0.14,0.20} respectively were assigned by the expert. The decision makers use q-RON $S_{f}N_{s}$, as indicated in Table 2, to deliver their assessments after weighing each alternative against these parameters.

**Table 2:** Represent q-RON $S_{f}N_{s}$${₳}_{{Ϧ}_{\text{ij}}}$ = $\left({Ʈ}_{\text{ij}},{ƚ}_{\text{ij}},{Ƒ}_{\text{ij}}\right)$ for q ≥ 3.

₷	$g_{1}$	$g_{2}$	$g_{3}$	$g_{4}$
$h_{1}$	(.77,.50,.30)	(.70,.40,.10)	(.55,.35,.20)	(.87,.42,.40)
$h_{2}$	(.67,.30,.30)	(.66,.25,.30)	(.77,.40,.30)	(.80,.10,.30)
$h_{3}$	(.61,.40,.30)	(.50,.10,.20)	(.87,.44,.10)	(.57,.22,.50)
$h_{4}$	(.88,.30,.20)	(.99,.10,.25)	(.44,.10,.30)	(.50,.33,.70)

By equation (10) we have

$$q‐\text{RON}\;S_{f}\;\text{WA}\;\left({₳}_{{Ϧ}_{11}},{₳}_{{Ϧ}_{12}},\ldots .,{₳}_{{Ϧ}_{44}}\right)=\left(\begin{array}{c} \sqrt[{q}]{1-\prod_{j=1}^{m}{\left(\prod_{i=1}^{n}{\left(1-{Ʈ}_{ij}^{q}\right)}^{\omega_{i}}\right)}^{\upsilon_{j}}},\\ \Pi_{j=1}^{m}{\left(\Pi_{i=1}^{n}{ƚ}_{ij}^{\omega_{i}}\right)}^{\upsilon_{j}},\\ \Pi_{j=1}^{m}{\left(\Pi_{i=1}^{n}{Ƒ}_{ij}^{\omega_{i}}\right)}^{\upsilon_{j}} \end{array}\right)$$

=(0.7844,0.2792,0.2758)

### 3.2 Ordered weighted averaging operator based on q-RONS set

Consider the collection of q-RONF $S_{f}N_{s}\;{₳}_{{Ϧ}_{ij}}$ = $\left({Ʈ}_{\text{ij}},{ƚ}_{\text{ij}},{Ƒ}_{\text{ij}}\right)$ for variable i from 1 to n and j from 1 to m, having weight vector for experts $x_{i}$ is $\omega_{i}$ and for parameters $e_{j}$ is $\upsilon_{j}\text{,}$ with $\omega_{i}$, $\upsilon_{j}\in \left[0,1\right]\;\text{such}\;\text{that}\;\sum_{i=1}^{n}\omega_{i}=1\;\text{and}\;\sum_{j=1}^{m}\upsilon_{j}=1$ respectively. Then q-RON $S_{f}$ OWA operator is defined by the mapping q-RON $S_{f}$ OWA: ${Đ}^{n}⟶$ Đ, where Đ represents the collection of q-RONF $S_{f}N_{s}$.

$$q‐\text{RONF}\;S_{f}O\;\text{WA}\;\left({₳}_{{Ϧ}_{11}},{₳}_{{Ϧ}_{12}},\ldots .,{₳}_{{Ϧ}_{nm}}\right)={⊕}_{j=1}^{m}\upsilon_{j}\left({⊕}_{i=1}^{n}\omega_{i}{₳}_{\sigma {Ϧ}_{ij}}\right)$$

Theorem 3 *Let*${₳}_{{Ϧ}_{ij}}$*=*$\left({Ʈ}_{ij},{ƚ}_{ij},{Ƒ}_{ij}\right)$*∀ i*,*j rang from i to* 1 *and j to m*, *be the collection of q-RON* $S_{f}N_{s}$. *Then the aggregation result for q-RON* $S_{f}O$ *WA operator is defined as*:

$$q‐\text{RON}\;S_{f}O\;\text{WA}\;\left({₳}_{{Ϧ}_{11}},{₳}_{{Ϧ}_{12}},\ldots .,{₳}_{{Ϧ}_{nm}}\right)={⊕}_{j=1}^{m}\upsilon_{j}\left({⊕}_{i=1}^{n}\omega_{i}{₳}_{{\sigma Ϧ}_{ij}}\right)$$

$$=\left(\sqrt[{q}]{1-\prod_{j=1}^{m}{\left(\prod_{i=1}^{n}{\left(1-{Ʈ}_{\sigma ij}^{q}\right)}^{\omega_{i}}\right)}^{\upsilon_{j}}},\Pi_{j=1}^{m}{\left(\Pi_{i=1}^{n}{ƚ}_{\sigma ij}^{\omega_{i}}\right)}^{\upsilon_{j}},\Pi_{j=1}^{m}{\left(\Pi_{i=1}^{n}{Ƒ}_{\sigma ij}^{\omega_{i}}\right)}^{\upsilon_{j}}\right)$$

(11) $$q‐\text{RON}\;S_{f}O\;\text{WA}\;\left({₳}_{{Ϧ}_{11}},{₳}_{{Ϧ}_{12}},\ldots .,{₳}_{{Ϧ}_{nm}}\right)=\left(\begin{array}{c} \sqrt[{q}]{1-\prod_{j=1}^{m}{\left(\prod_{i=1}^{n}{\left(1-{Ʈ}_{\sigma ij}^{q}\right)}^{\omega_{i}}\right)}^{\upsilon_{j}}},\\ \Pi_{j=1}^{m}{\left(\Pi_{i=1}^{n}{ƚ}_{\sigma ij}^{\omega_{i}}\right)}^{\upsilon_{j}},\\ \Pi_{j=1}^{m}{\left(\Pi_{i=1}^{n}{Ƒ}_{\sigma ij}^{\omega_{i}}\right)}^{\upsilon_{j}} \end{array}\right)$$

Where WV of for expert x_i_ is $\omega_{i}$ and for parameters e_j_ is $\upsilon_{j}$ respectively, such that $\sum_{i=1}^{n}\omega_{i}=1$ and $\sum_{j=1}^{m}\upsilon_{j}=1\text{.}$

Equation (11) represent the q-RONWA operator.

Proof**:** Straightforward as the above result of " q-RON $S_{f}$ WA ″ operator.

Example 3 From the above Example No.2 of "Table 2", we consider the collections of q-RON $S_{f}N_{s}\;{₳}_{{Ϧ}_{ij}}$ = $\left({Ʈ}_{ij},{ƚ}_{ij},{Ƒ}_{ij}\right)$, to get the tabular representation of ${₳}_{{Ϧ}_{ij}}$ = $\left({Ʈ}_{\sigma ij},{ƚ}_{\sigma ij},{Ƒ}_{\sigma ij}\right)$ is expressed in "Table 3" by using the score function.

**Table 3:** Represent q-RON $S_{f}N_{s}$${₳}_{{\sigma Ϧ}_{\text{ij}}}$ = $\left({Ʈ}_{\sigma \text{ij}},{ƚ}_{\sigma \text{ij}},{Ƒ}_{\sigma \text{ij}}\right)$ for q ≥ 3.

₷	$g_{1}$	$g_{2}$	$g_{3}$	$g_{4}$
$h_{1}$	(0.88,0.30,0.20)	(0.99,0.10,0.25)	(0.87,0.44,0.10)	(0.87,0.42,0.40)
$h_{2}$	(0.77,0.50,0.30)	(0.70,0.40,0.10)	(0.77,0.40,0.30)	(0.80,0.10,0.30)
$h_{3}$	(0.67,0.30,0.30)	(0.66,0.25,0.30)	(0.55,0.35,0.20)	(0.66,0.40,0.30)
$h_{4}$	(0.61,0.40,0.30)	(0.51,0.40,0.30)	(0.44,0.10,0.30)	(0.57,0.22,0.5)

By equation (11) we have

$$q‐\text{RON}\;S_{f}O\;\text{WA}\;\left({₳}_{{Ϧ}_{11}},{₳}_{{Ϧ}_{12}},\ldots .,{₳}_{{Ϧ}_{nm}}\right)=\left(\begin{array}{c} \sqrt[{q}]{1-\prod_{j=1}^{m}{\left(\prod_{i=1}^{n}{\left(1-{Ʈ}_{\sigma ij}^{q}\right)}^{\omega_{i}}\right)}^{\upsilon_{j}}},\\ \Pi_{j=1}^{m}{\left(\Pi_{i=1}^{n}{ƚ}_{\sigma ij}^{\omega_{i}}\right)}^{\upsilon_{j}},\\ \Pi_{j=1}^{m}{\left(\Pi_{i=1}^{n}{Ƒ}_{\sigma ij}^{\omega_{i}}\right)}^{\upsilon_{j}} \end{array}\right)$$

=(0.7657,0.3084,0.2725).

### 3.3 Weighted geometric operator based on q-RONS set

Consider the collection of q-RONF $S_{f}N_{s}\;{₳}_{{Ϧ}_{ij}}$ = $\left({Ʈ}_{\text{ij}},{ƚ}_{\text{ij}},{Ƒ}_{\text{ij}}\right)$ for i,j rang from i to 1 and j to m, having weight vector for experts $x_{i}$ is $\omega_{i}$ and for parameters $e_{j}$ is $\upsilon_{j}\text{,}$ with $\omega_{i}$, $\upsilon_{j}\in \left[0,1\right]\;\text{such}\;\text{that}\;\sum_{i=1}^{n}\omega_{i}=1\;\text{and}\;\sum_{j=1}^{m}\upsilon_{j}=1$ respectively. Then q-RON $S_{f}$ WG operator is defined by the mapping q-RON $S_{f}$ WG: ${Đ}^{n}⟶$ Đ, where Đ represents the collection of q-RONF $S_{f}N_{s}$.

$$q-\text{RON}\;S_{f}\;\text{WG}\;\left({₳}_{{Ϧ}_{11}},{₳}_{{Ϧ}_{12}},\ldots .,{₳}_{{Ϧ}_{\text{nm}}}\right)={⊗}_{j=1}^{m}{\left({⊗}_{i=1}^{n}{₳}_{{Ϧ}_{ij}}^{\omega_{i}}\right)}^{\upsilon_{j}}$$

Theorem 4 Let ${₳}_{{\ddot{e}}_{\text{ij}}}$ = $\left({Ʈ}_{\text{ij}},{ƚ}_{\text{ij}},{Ƒ}_{\text{ij}}\right)$ ∀ i,j rang from i to 1 and j to m, be the collection of $q-\text{RON}S_{f}N_{s}$. Then for q-RON $S_{f}$ WG operator the aggregation result is defined:

$$q‐\text{RON}\;S_{f}\;\text{WG}\;\left({₳}_{{Ϧ}_{11}},{₳}_{{Ϧ}_{12}},\ldots .,{₳}_{{Ϧ}_{\text{nm}}}\right)={⊗}_{j=1}^{m}{\left({⊗}_{i=1}^{n}{₳}_{{Ϧ}_{ij}}^{\omega_{i}}\right)}^{\upsilon_{j}}$$

$$=\left(\Pi_{j=1}^{m}{\left(\Pi_{i=1}^{n}{Ʈ}_{ij}^{\omega_{i}}\right)}^{\upsilon_{j}},\sqrt[{q}]{1-\prod_{j=1}^{m}{\left(\prod_{i=1}^{n}{\left(1-{ƚ}_{ij}^{q}\right)}^{\omega_{i}}\right)}^{\upsilon_{j}}},\sqrt[{q}]{1-\prod_{j=1}^{m}{\left(\prod_{i=1}^{n}{\left(1-{Ƒ}_{ij}^{q}\right)}^{\omega_{i}}\right)}^{\upsilon_{j}}}\right)$$

(12) $$q‐\text{RON}\;S_{f}\;\text{WG}\;\left({₳}_{{Ϧ}_{11}},{₳}_{{Ϧ}_{12}},\ldots .,{₳}_{{Ϧ}_{\text{nm}}}\right)=\left(\begin{array}{c} \Pi_{j=1}^{m}{\left(\Pi_{i=1}^{n}{Ʈ}_{ij}^{\omega_{i}}\right)}^{\upsilon_{j}},\\ \sqrt[{q}]{1-\prod_{j=1}^{m}{\left(\prod_{i=1}^{n}{\left(1-{ƚ}_{ij}^{q}\right)}^{\omega_{i}}\right)}^{\upsilon_{j}}},\\ \sqrt[{q}]{1-\prod_{j=1}^{m}{\left(\prod_{i=1}^{n}{\left(1-{Ƒ}_{ij}^{q}\right)}^{\omega_{i}}\right)}^{\upsilon_{j}}} \end{array}\right)$$

Where WV of for expert x_i_ is $\omega_{i}$ and for parameters e_j_ is $\upsilon_{j}$ respectively, such that $\sum_{i=1}^{n}\omega_{i}=1$ and $\sum_{j=1}^{m}\upsilon_{j}=1\text{.}$

Equation (12) represent the q-RONSWG operator.

Proof**:** Straightforward.

### 3.4 Ordered weighted geometric operator based on q-RONS set

Consider the collection of q-RONF $S_{f}N_{s}\;{₳}_{{Ϧ}_{ij}}$ = $\left({Ʈ}_{\text{ij}},{ƚ}_{\text{ij}},{Ƒ}_{\text{ij}}\right)$ for i,j rang from i to 1 and j to m, having weight vector for experts $x_{i}$ is $\omega_{i}$ and for parameters $e_{j}$ is $\upsilon_{j}\text{,}$ with $\omega_{i}$, $\upsilon_{j}\in \left[0,1\right]\;\text{such}\;\text{that}\;\sum_{i=1}^{n}\omega_{i}=1\;\text{and}\;\sum_{j=1}^{m}\upsilon_{j}=1$ respectively. Then q-RON $S_{f}$ OWG operator is defined by the mapping q-RON $S_{f}$ OWG: ${Đ}^{n}⟶$ Đ, where Đ represents the collection of q-RONF $S_{f}N_{s}$.

$$q‐\text{RON}\;S_{f}\;\text{OWG}\;\left({₳}_{{Ϧ}_{11}},{₳}_{{Ϧ}_{12}},\ldots .,{₳}_{{Ϧ}_{\text{nm}}}\right)={⊗}_{j=1}^{m}{\left({⊗}_{i=1}^{n}{₳}_{{\sigma Ϧ}_{ij}}^{\omega_{i}}\right)}^{\upsilon_{j}}$$

Theorem 5 *Let*${₳}_{{Ϧ}_{ij}}$*=*$\left({Ʈ}_{ij},{ƚ}_{ij},{Ƒ}_{ij}\right)$*for i*,*j rang from i to* 1 *and j to m*, *be the collection of q-RON* $S_{f}N_{s}$. *Then for q-RON* $S_{f}$ *OWG operator the aggregation result is defined*:

$$q‐\text{RON}\;S_{f}\;\text{OWG}\;\left({₳}_{{Ϧ}_{11}},{₳}_{{Ϧ}_{12}},\ldots .,{₳}_{{Ϧ}_{\text{nm}}}\right)={⊗}_{j=1}^{m}{\left({⊗}_{i=1}^{n}{₳}_{{\sigma Ϧ}_{ij}}^{\omega_{i}}\right)}^{\upsilon_{j}}$$

$$=\left(\Pi_{j=1}^{m}{\left(\Pi_{i=1}^{n}{Ʈ}_{\sigma ij}^{\omega_{i}}\right)}^{\upsilon_{j}},\sqrt[{q}]{1-\prod_{j=1}^{m}{\left(\prod_{i=1}^{n}{\left(1-{ƚ}_{\sigma ij}^{q}\right)}^{\omega_{i}}\right)}^{\upsilon_{j}}},\sqrt[{q}]{1-\prod_{j=1}^{m}{\left(\prod_{i=1}^{n}{\left(1-{Ƒ}_{\sigma ij}^{q}\right)}^{\omega_{i}}\right)}^{\upsilon_{j}}}\right)$$

(13) $$q‐\text{RON}\;S_{f}\;\text{OWG}\;\left({₳}_{{Ϧ}_{11}},{₳}_{{Ϧ}_{12}},\ldots .,{₳}_{{Ϧ}_{\text{nm}}}\right)=\left(\begin{array}{c} \Pi_{j=1}^{m}{\left(\Pi_{i=1}^{n}{Ʈ}_{\sigma ij}^{\omega_{i}}\right)}^{\upsilon_{j}},\\ \sqrt[{q}]{1-\prod_{j=1}^{m}{\left(\prod_{i=1}^{n}{\left(1-{ƚ}_{\sigma ij}^{q}\right)}^{\omega_{i}}\right)}^{\upsilon_{j}}},\\ \sqrt[{q}]{1-\prod_{j=1}^{m}{\left(\prod_{i=1}^{n}{\left(1-{Ƒ}_{\sigma ij}^{q}\right)}^{\omega_{i}}\right)}^{\upsilon_{j}}} \end{array}\right)$$

Where ${₳}_{{\sigma Ϧ}_{\text{ij}}}$ = $\left({Ʈ}_{\sigma \text{ij}},{ƚ}_{\sigma \text{ij}},{Ƒ}_{\sigma \text{ij}}\right)$ expressed the permutations of $i^{\text{th}}$ and $j^{\text{th}}$ largest values of the collection of i×j of q-RON $S_{f}N_{s}$.

Equation (13) represent the q-RONSOWG operator.

Proof**:** Straightforward.

### 3.5 Some special properties related based on proposed AOs

The following properties are held by $q-RONS_{f}WA$, $q-RONS_{f}OWA$, $q-RONS_{f}WG$ and $q-RONS_{f}OWG$ operators.

Property 1 (*Idempotency*):

a. If ${₳}_{{Ϧ}_{\text{ij}}}$ = ${₦}_{Ϧ}$ , where ${₦}_{Ϧ=}\left(₩,₲,₱\right)$, then q-RON $S_{f}$ WA $\left({₳}_{{Ϧ}_{11}},{₳}_{{Ϧ}_{12}},\ldots .,{₳}_{{Ϧ}_{\text{nm}}}\right)$ = ${₦}_{Ϧ}$.
b. If ${₳}_{{Ϧ}_{\text{ij}}}$ = ${₦}_{Ϧ}$ , where ${₦}_{Ϧ=}\left(₩,₲,₱\right)$, then q-RON $S_{f}$ OWA $\left({₳}_{{Ϧ}_{11}},{₳}_{{Ϧ}_{12}},\ldots .,{₳}_{{Ϧ}_{\text{nm}}}\right)$ = ${₦}_{Ϧ}$.
c. If ${₳}_{{Ϧ}_{\text{ij}}}$ = ${₦}_{Ϧ}$ , where ${₦}_{Ϧ=}\left(₩,₲,₱\right)$, then q-RON $S_{f}$ WG $\left({₳}_{{Ϧ}_{11}},{₳}_{{Ϧ}_{12}},\ldots .,{₳}_{{Ϧ}_{\text{nm}}}\right)$ = ${₦}_{Ϧ}$.
d. If ${₳}_{{Ϧ}_{\text{ij}}}$ = ${₦}_{Ϧ}$ , where ${₦}_{Ϧ=}\left(₩,₲,₱\right)$, then q-RON $S_{f}$ OWG $\left({₳}_{{Ϧ}_{11}},{₳}_{{Ϧ}_{12}},\ldots .,{₳}_{{Ϧ}_{\text{nm}}}\right)$ = ${₦}_{Ϧ}$.

Proof**(a):** It is given that ${₳}_{{Ϧ}_{\text{ij}}}={₦}_{Ϧ}=\left(₩,₲,₱\right)\text{,}$ then

$$q‐\text{RON}\;S_{f}\;\text{WA}\;\left({₳}_{{Ϧ}_{11}},{₳}_{{Ϧ}_{12}},\ldots .,{₳}_{{Ϧ}_{\text{nm}}}\right)=\left(\begin{array}{c} \sqrt[{q}]{1-\prod_{j=1}^{m}{\left(\prod_{i=1}^{n}{\left(1-{Ʈ}_{ij}^{q}\right)}^{\omega_{i}}\right)}^{\upsilon_{j}}},\\ \Pi_{j=1}^{m}{\left(\Pi_{i=1}^{n}{ƚ}_{ij}^{\omega_{i}}\right)}^{\upsilon_{j}},\\ \Pi_{j=1}^{m}{\left(\Pi_{i=1}^{n}{Ƒ}_{ij}^{\omega_{i}}\right)}^{\upsilon_{j}} \end{array}\right)$$

$$=\left(\begin{array}{c} \sqrt[{q}]{1-\prod_{j=1}^{m}{\left(\prod_{i=1}^{n}{\left(1-{₩}^{q}\right)}^{\omega_{i}}\right)}^{\upsilon_{j}}},\\ \Pi_{j=1}^{m}{\left(\Pi_{i=1}^{n}{₲}^{q}\right)}^{\upsilon_{j}},\\ \Pi_{j=1}^{m}{\left(\Pi_{i=1}^{n}{₱}^{q}\right)}^{\upsilon_{j}} \end{array}\right)\;⟹\left(\sqrt[{q}]{1-\left(1-{₩}^{q}\right)},{₲}^{q},{₱}^{q}\right)$$

Hence, q-RON $S_{f}$ WA $\left({₳}_{{Ϧ}_{11}},{₳}_{{Ϧ}_{12}},\ldots .,{₳}_{{Ϧ}_{\text{nm}}}\right)$ = ${₦}_{Ϧ}$.

Proof**(b),(c),(d):** Straightforward as above.

Property 2 (Boundedness):

a. If ${₳}_{{Ϧ}_{\text{ij}}}^{+}$ = $\left\{\max_{j}\max_{i}\left({Ʈ}_{\text{ij}}\right),\min_{j}\min_{i}\left({ƚ}_{\text{ij}}\right),\min_{j}\min_{i}\left({Ƒ}_{\text{ij}}\right)\right\}$ and ${₳}_{{Ϧ}_{\text{ij}}}^{-}$ = $\left\{\min_{j}\underset{i}{\;\min}\left({Ʈ}_{\text{ij}}\right),\max_{j}\underset{i}{\;\max}\left({ƚ}_{\text{ij}}\right),\max_{j}\underset{i}{\;\max}\left({Ƒ}_{\text{ij}}\right)\right\}$, then ${₳}_{{Ϧ}_{\text{ij}}}^{-}\leq q-\text{RON}S_{f}\text{WA}\;\left({₳}_{{Ϧ}_{11}},{₳}_{{Ϧ}_{12}},\ldots .,{₳}_{{Ϧ}_{\text{nm}}}\right)\leq {₳}_{{Ϧ}_{\text{ij}}}^{+}$.
b. If ${₳}_{{Ϧ}_{\text{ij}}}^{+}$ = $\left\{\max_{j}\max_{i}\left({Ʈ}_{\text{ij}}\right),\min_{j}\min_{i}\left({ƚ}_{\text{ij}}\right),\min_{j}\min_{i}\left({Ƒ}_{\text{ij}}\right)\right\}$ and ${₳}_{{Ϧ}_{\text{ij}}}^{-}$ = $\left\{\min_{j}\underset{i}{\;\min}\left({Ʈ}_{\text{ij}}\right),\max_{j}\;\max_{i}\left({ƚ}_{\text{ij}}\right),\max_{j}\max_{i}\left({Ƒ}_{\text{ij}}\right)\right\}$, then ${₳}_{{Ϧ}_{\text{ij}}}^{-}\leq q-\text{RON}S_{f}\text{OWA}\;\left({₳}_{{Ϧ}_{11}},{₳}_{{Ϧ}_{12}},\ldots .,{₳}_{{Ϧ}_{\text{nm}}}\right)\leq {₳}_{{Ϧ}_{\text{ij}}}^{+}$.
c. If ${₳}_{{Ϧ}_{\text{ij}}}^{+}$ = $\left\{\max_{j}\max_{i}\left({Ʈ}_{\text{ij}}\right),\min_{j}\min_{i}\left({ƚ}_{\text{ij}}\right),\min_{j}\min_{i}\left({Ƒ}_{\text{ij}}\right)\right\}$ and ${₳}_{{Ϧ}_{\text{ij}}}^{-}$ = $\left\{\min_{j}\underset{i}{\;\min}\left({Ʈ}_{\text{ij}}\right),\max_{j}\;\max_{i}\left({ƚ}_{\text{ij}}\right),\max_{j}\underset{i}{\;\max}\left({Ƒ}_{\text{ij}}\right)\right\}$, then ${₳}_{{Ϧ}_{\text{ij}}}^{-}\leq q-\text{RON}S_{f}\text{WG}\;\left({₳}_{{Ϧ}_{11}},{₳}_{{Ϧ}_{12}},\ldots .,{₳}_{{Ϧ}_{\text{nm}}}\right)\leq {₳}_{{Ϧ}_{\text{ij}}}^{+}$.
d. If ${₳}_{{Ϧ}_{\text{ij}}}^{+}$ = $\left\{\max_{j}\max_{i}\left({Ʈ}_{\text{ij}}\right),\min_{j}\min_{i}\left({ƚ}_{\text{ij}}\right),\min_{j}\min_{i}\left({Ƒ}_{\text{ij}}\right)\right\}$ and ${₳}_{{Ϧ}_{\text{ij}}}^{-}$ = $\left\{\min_{j}\underset{i}{\;\min}\left({Ʈ}_{\text{ij}}\right),\max_{j}\underset{i}{\;\max}\left({ƚ}_{\text{ij}}\right),\max_{j}\underset{i}{\;\max}\left({Ƒ}_{\text{ij}}\right)\right\}$, then ${₳}_{{Ϧ}_{\text{ij}}}^{-}\leq q-\text{RON}S_{f}\text{OWG}\;\left({₳}_{{Ϧ}_{11}},{₳}_{{Ϧ}_{12}},\ldots .,{₳}_{{Ϧ}_{\text{nm}}}\right)\leq {₳}_{{Ϧ}_{\text{ij}}}^{+}$.

Proof**(a):** We know that

$${₳}_{{Ϧ}_{\text{ij}}}^{-}=\left\{\min_{j}\min_{i}\left({Ʈ}_{\text{ij}}\right),\max_{j}\max_{i}\left({ƚ}_{\text{ij}}\right),\max_{j}\max_{i}\left({Ƒ}_{\text{ij}}\right)\right\}$$

and

$${₳}_{{Ϧ}_{\text{ij}}}^{+}=\left\{\max_{j}\underset{i}{\;\max}\left({Ʈ}_{\text{ij}}\right),\min_{j}\underset{i}{\;\min}\left({ƚ}_{\text{ij}}\right),\min_{j}\underset{i}{\;\min}\left({Ƒ}_{\text{ij}}\right)\right\}\text{,}$$

To show that,

$${₳}_{{Ϧ}_{\text{ij}}}^{-}\leq q-\text{RON}S_{f}\text{WA}\;\left({₳}_{{Ϧ}_{11}},{₳}_{{Ϧ}_{12}},\ldots .,{₳}_{{Ϧ}_{\text{nm}}}\right)\leq {₳}_{{Ϧ}_{\text{ij}}}^{+}\text{.}$$

$$⟹\;\min_{j}\underset{i}{\;\min}\left\{{Ʈ}_{\text{ij}}\right\}\leq {Ʈ}_{\text{ij}}\leq \max_{j}\underset{i}{\;\max}\left\{{Ʈ}_{\text{ij}}\right\}$$

$$\Leftrightarrow \;1-\;\max_{j}\max_{i}\left\{{Ʈ}_{ij}^{q}\right\}\leq 1-{Ʈ}_{ij}^{q}\leq 1-\min_{j}\min_{i}\left\{{Ʈ}_{ij}^{q}\right\}$$

$$\Leftrightarrow \;\Pi_{j=1}^{m}{\left(\Pi_{i=1}^{n}{\left(1-\max_{j}\max_{i}\left\{{Ʈ}_{ij}^{q}\right\}\right)}^{\omega_{i}}\right)}^{\upsilon_{j}}\leq \Pi_{j=1}^{m}{\left(\Pi_{i=1}^{n}{\left(1-{Ʈ}_{ij}^{q}\right)}^{\omega_{i}}\right)}^{\upsilon_{j}}\leq \Pi_{j=1}^{m}{\left(\Pi_{i=1}^{n}{\left(1-\min_{j}\min_{i}\left\{{Ʈ}_{ij}^{q}\right\}\right)}^{\omega_{i}}\right)}^{\upsilon_{j}}$$

$$\Leftrightarrow \;{\left({\left(1-\max_{j}\max_{i}\left\{{Ʈ}_{ij}^{q}\right\}\right)}^{\sum_{i=1}^{n}\omega_{i}}\right)}^{\sum_{j=1}^{m}\upsilon_{j}}\leq \Pi_{j=1}^{m}{\left(\Pi_{i=1}^{n}{\left(1-{Ʈ}_{ij}^{q}\right)}^{\omega_{i}}\right)}^{\upsilon_{j}}\leq {\left({\left(1-\min_{j}\min_{i}\left\{{Ʈ}_{ij}^{q}\right\}\right)}^{\sum_{i=1}^{n}\omega_{i}}\right)}^{\sum_{i=1}^{m}\upsilon_{j}}$$

$$\Leftrightarrow \;\left(1-\max_{j}\max_{i}\left\{{Ʈ}_{ij}^{q}\right\}\right)\leq \Pi_{j=1}^{m}{\left(\Pi_{i=1}^{n}{\left(1-{Ʈ}_{ij}^{q}\right)}^{\omega_{i}}\right)}^{\upsilon_{j}}\leq \left(1-\min_{j}\min_{i}\left\{{Ʈ}_{ij}^{q}\right\}\right)$$

$$\Leftrightarrow \;1-\;\left(1-\max_{j}\max_{i}\left\{{Ʈ}_{ij}^{q}\right\}\right)\leq 1-\Pi_{j=1}^{m}{\left(\Pi_{i=1}^{n}{\left(1-{Ʈ}_{ij}^{q}\right)}^{\omega_{i}}\right)}^{\upsilon_{j}}\leq 1-\left(1-\min_{j}\min_{i}\left\{{Ʈ}_{ij}^{q}\right\}\right)$$

$$\Leftrightarrow \;\min_{j}\min_{i}\left\{{Ʈ}_{\text{ij}}\right\}\leq \sqrt[{q}]{1-\Pi_{j=1}^{m}{\left(\Pi_{i=1}^{n}{\left(1-{Ʈ}_{ij}^{q}\right)}^{\omega_{i}}\right)}^{\upsilon_{j}}}\leq \max_{j}\max_{i}\left\{{Ʈ}_{\text{ij}}\right\}$$

Next, we have

$$\Leftrightarrow \;\min_{j}\min_{i}\left\{{ƚ}_{\text{ij}}\right\}\leq {ƚ}_{\text{ij}}\leq \max_{j}\max_{i}\left\{{ƚ}_{\text{ij}}\right\}$$

$$\Leftrightarrow \;\Pi_{j=1}^{m}{\left(\Pi_{i=1}^{n}{\left(\min_{j}\min_{i}\left\{{ƚ}_{\text{ij}}\right\}\right)}^{\omega_{i}}\right)}^{\upsilon_{j}}\leq \Pi_{j=1}^{m}{\left(\Pi_{i=1}^{n}{\left({ƚ}_{\text{ij}}\right)}^{\omega_{i}}\right)}^{\upsilon_{j}}\leq \Pi_{j=1}^{m}{\left(\Pi_{i=1}^{n}{\left(\max_{j}\max_{i}\left\{{ƚ}_{\text{ij}}\right\}\right)}^{\omega_{i}}\right)}^{\upsilon_{j}}$$

$$\Leftrightarrow \;{\left({\left(\min_{j}\min_{i}\left\{{ƚ}_{\text{ij}}\right\}\right)}^{\sum_{i=1}^{n}\omega_{i}}\right)}^{\sum_{j=1}^{m}\upsilon_{j}}\leq \Pi_{j=1}^{m}{\left(\Pi_{i=1}^{n}{\left({ƚ}_{\text{ij}}\right)}^{\omega_{i}}\right)}^{\upsilon_{j}}\leq {\left({\left(\max_{j}\max_{i}\left\{{ƚ}_{\text{ij}}\right\}\right)}^{\sum_{i=1}^{n}\omega_{i}}\right)}^{\sum_{j=1}^{m}\upsilon_{j}}$$

$$\Leftrightarrow \;\min_{j}\min_{i}\left\{{ƚ}_{\text{ij}}\right\}\leq \Pi_{j=1}^{m}{\left(\Pi_{i=1}^{n}{\left(1-{ƚ}_{ij}^{q}\right)}^{\omega_{i}}\right)}^{\upsilon_{j}}\leq \max_{j}\max_{i}\left\{{ƚ}_{\text{ij}}\right\}$$

And finally

$$\Leftrightarrow \;\min_{j}\min_{i}\left\{{Ƒ}_{\text{ij}}\right\}\leq {Ƒ}_{\text{ij}}\leq \max_{j}\max_{i}\left\{{Ƒ}_{\text{ij}}\right\}$$

$$\Leftrightarrow \;\Pi_{j=1}^{m}{\left(\Pi_{i=1}^{n}{\left(\min_{j}\min_{i}\left\{{Ƒ}_{\text{ij}}\right\}\right)}^{\omega_{i}}\right)}^{\upsilon_{j}}\leq \Pi_{j=1}^{m}{\left(\Pi_{i=1}^{n}{\left({Ƒ}_{\text{ij}}\right)}^{\omega_{i}}\right)}^{\upsilon_{j}}\leq \Pi_{j=1}^{m}{\left(\Pi_{i=1}^{n}{\left(\max_{j}\max_{i}\left\{{Ƒ}_{\text{ij}}\right\}\right)}^{\omega_{i}}\right)}^{\upsilon_{j}}$$

$$\Leftrightarrow \;{\left({\left(\min_{j}\min_{i}\left\{{Ƒ}_{\text{ij}}\right\}\right)}^{\sum_{i=1}^{n}\omega_{i}}\right)}^{\sum_{j=1}^{m}\upsilon_{j}}\leq \Pi_{j=1}^{m}{\left(\Pi_{i=1}^{n}{\left({Ƒ}_{\text{ij}}\right)}^{\omega_{i}}\right)}^{\upsilon_{j}}\leq {\left({\left(\max_{j}\max_{i}\left\{{Ƒ}_{\text{ij}}\right\}\right)}^{\sum_{i=1}^{n}\omega_{i}}\right)}^{\sum_{j=1}^{m}\upsilon_{j}}$$

$$\Leftrightarrow \;\min_{j}\min_{i}\left\{{Ƒ}_{\text{ij}}\right\}\leq \Pi_{j=1}^{m}{\left(\Pi_{i=1}^{n}{\left({Ƒ}_{\text{ij}}\right)}^{\omega_{i}}\right)}^{\upsilon_{j}}\leq \max_{j}\max_{i}\left\{{Ƒ}_{\text{ij}}\right\}$$

Therefore,

$$\Leftrightarrow \;\min_{j}\min_{i}\left\{{Ʈ}_{\text{ij}}\right\}\leq \sqrt[{q}]{1-\Pi_{j=1}^{m}{\left(\Pi_{i=1}^{n}{\left(1-{Ʈ}_{ij}^{q}\right)}^{\omega_{i}}\right)}^{\upsilon_{j}}}\leq \max_{j}\max_{i}\left\{{Ʈ}_{\text{ij}}\right\}$$

$$\Leftrightarrow \;\min_{j}\min_{i}\left\{{ƚ}_{\text{ij}}\right\}\leq \Pi_{j=1}^{m}{\left(\Pi_{i=1}^{n}{\left(1-{ƚ}_{ij}^{q}\right)}^{\omega_{i}}\right)}^{\upsilon_{j}}\leq \max_{j}\max_{i}\left\{{ƚ}_{\text{ij}}\right\}$$

$$\Leftrightarrow \;\min_{j}\min_{i}\left\{{Ƒ}_{\text{ij}}\right\}\leq \Pi_{j=1}^{m}{\left(\Pi_{i=1}^{n}{\left({Ƒ}_{\text{ij}}\right)}^{\omega_{i}}\right)}^{\upsilon_{j}}\leq \max_{j}\max_{i}\left\{{Ƒ}_{\text{ij}}\right\}$$

Let δ = q-RON $S_{f}$ WA $\left({₳}_{{Ϧ}_{11}},{₳}_{{Ϧ}_{12}},\ldots .,{₳}_{{Ϧ}_{\text{nm}}}\right)=\left({Ʈ}_{\delta },{ƚ}_{\delta },{Ƒ}_{\delta }\right)$, then by the score function

$$S\;\left(\delta \right)={Ʈ}_{\delta }^{q}\;-\;{ƚ}_{\delta }^{q}-{Ƒ}_{\delta }^{q}+\left(\frac{e^{{Ʈ}_{\delta }^{q}-{ƚ}_{\delta }^{q}-{Ƒ}_{\delta }^{q}}}{e^{{Ʈ}_{\delta }^{q}-{ƚ}_{\delta }^{q}-{Ƒ}_{\delta }^{q}}+1}-\frac{1}{2}\right)\pi_{\delta }^{q}$$

$$\leq {\left(\max_{j}\max_{i}\left\{{Ʈ}_{\text{ij}}\right\}\right)}^{q}-{\left(\min_{j}\min_{i}\left\{{ƚ}_{\text{ij}}\right\}\right)}^{q}-{\left(\min_{j}\min_{i}\left\{{Ƒ}_{\text{ij}}\right\}\right)}^{q}+\left(\frac{e^{{\left(\max_{j}\max_{i}\left\{{Ʈ}_{\text{ij}}\right\}\right)}^{q}-{\left(\min_{j}\min_{i}\left\{{ƚ}_{\text{ij}}\right\}\right)}^{q}-{\left(\min_{j}\min_{i}\left\{{Ƒ}_{\text{ij}}\right\}\right)}^{q}}}{e^{\;{\left(\max_{j}\max_{i}\left\{{Ʈ}_{\text{ij}}\right\}\right)}^{q}-{\left(\min_{j}\min_{i}\left\{{ƚ}_{\text{ij}}\right\}\right)}^{q}-{\left(\min_{j}\min_{i}\left\{{Ƒ}_{\text{ij}}\right\}\right)}^{q}}+1}-\frac{1}{2}\right)\pi_{{\tilde{N}}_{{Ϧ}_{\text{ij}}}^{+}}^{q}$$

$$=S\;\left({₳}_{{Ϧ}_{\text{ij}}}^{+}\right)$$

$$\Rightarrow \;S\;\left(\delta \right)\leq S\;\left({₳}_{{Ϧ}_{\text{ij}}}^{+}\right)$$

and

$$\Leftrightarrow \;S\;\left(\delta \right)={Ʈ}_{\delta }^{q}\;-\;{ƚ}_{\delta }^{q}-{Ƒ}_{\delta }^{q}+\left(\frac{e^{{Ʈ}_{\delta }^{q}-{ƚ}_{\delta }^{q}-{Ƒ}_{\delta }^{q}}}{e^{{Ʈ}_{\delta }^{q}-{ƚ}_{\delta }^{q}-{Ƒ}_{\delta }^{q}}+1}-\frac{1}{2}\right)\pi_{\delta }^{q}$$

$$\geq {\left(\min_{j}\min_{i}\left\{{Ʈ}_{\text{ij}}\right\}\right)}^{q}-{\left(\max_{j}\max_{i}\left\{{ƚ}_{\text{ij}}\right\}\right)}^{q}-{\left(\max_{j}\max_{i}\left\{{Ƒ}_{\text{ij}}\right\}\right)}^{q}+\left(\frac{e^{\;{\left(\min_{j}\min_{i}\left\{{Ʈ}_{\text{ij}}\right\}\right)}^{q}-\;{\left(\max_{j}\max_{i}\left\{{ƚ}_{\text{ij}}\right\}\right)}^{q}-\;{\left(\max_{j}\max_{i}\left\{{Ƒ}_{\text{ij}}\right\}\right)}^{q}}}{e^{\;{\left(\min_{j}\min_{i}\left\{{Ʈ}_{\text{ij}}\right\}\right)}^{q}-\;{\left(\max_{j}\max_{i}\left\{{ƚ}_{\text{ij}}\right\}\right)}^{q}-{\left(\max_{j}\max_{i}\left\{{Ƒ}_{\text{ij}}\right\}\right)}^{q}}+1}-\frac{1}{2}\right)\pi_{{₳}_{{Ϧ}_{\text{ij}}}^{-}}^{q}$$

$$=S\;\left({₳}_{{Ϧ}_{\text{ij}}}^{-}\right)$$

$$\Rightarrow \;S\;\left(\delta \right)\geq S\;\left({₳}_{{Ϧ}_{\text{ij}}}^{-}\right)$$

We encounter the following cases, with the above methodology.

i. By the comparison of two q-RON $S_{f}N_{s}$, we obtain ${₳}_{{Ϧ}_{\text{ij}}}^{-}<q-\text{RON}S_{f}\text{WA}\;\left({₳}_{{\ddot{e}}_{11}},{₳}_{{\ddot{e}}_{12}},\ldots .,{₳}_{{\ddot{e}}_{\text{nm}}}\right){<₳}_{{Ϧ}_{\text{ij}}}^{+}$ if we have S (δ) ≤ S $\left({₳}_{{Ϧ}_{\text{ij}}}^{+}\right)$ and S (δ) ≥ S $\left({₳}_{{Ϧ}_{\text{ij}}}^{-}\right)$.
ii. If S (δ) = S $\left({₳}_{{Ϧ}_{\text{ij}}}^{+}\right)$, then

$${Ʈ}_{\delta }^{q}\;-\;{ƚ}_{\delta }^{q}-{Ƒ}_{\delta }^{q}+\left(\frac{e^{{Ʈ}_{\delta }^{q}-{ƚ}_{\delta }^{q}-{Ƒ}_{\delta }^{q}}}{e^{{Ʈ}_{\delta }^{q}-{ƚ}_{\delta }^{q}-{Ƒ}_{\delta }^{q}}+1}-\frac{1}{2}\right)\pi_{\delta }^{q}$$

$$={\left(\max_{j}\max_{i}\left\{{Ʈ}_{\text{ij}}\right\}\right)}^{q}-{\left(\min_{j}\min_{i}\left\{{ƚ}_{\text{ij}}\right\}\right)}^{q}-{\left(\min_{j}\min_{i}\left\{{Ƒ}_{\text{ij}}\right\}\right)}^{q}+\left(\frac{e^{{\left(\max_{j}\max_{i}\left\{{Ʈ}_{\text{ij}}\right\}\right)}^{q}-{\left(\min_{j}\min_{i}\left\{{ƚ}_{\text{ij}}\right\}\right)}^{q}-{\left(\min_{j}\min_{i}\left\{{Ƒ}_{\text{ij}}\right\}\right)}^{q}}}{e^{\;{\left(\max_{j}\max_{i}\left\{{Ʈ}_{\text{ij}}\right\}\right)}^{q}-{\left(\min_{j}\min_{i}\left\{{ƚ}_{\text{ij}}\right\}\right)}^{q}-{\left(\min_{j}\min_{i}\left\{{Ƒ}_{\text{ij}}\right\}\right)}^{q}}+1}-\frac{1}{2}\right)\pi_{{₳}_{{Ϧ}_{\text{ij}}}^{+}}^{q}$$

Using the above inequalities, we get

$$⟹\;{Ʈ}_{\delta }=\max_{j}\max_{i}\left\{{Ʈ}_{\text{ij}}\right\},{ƚ}_{\delta }=\min_{j}\min_{i}\left({ƚ}_{\text{ij}}\right),{Ƒ}_{\delta }=\min_{j}\min_{i}\left({Ƒ}_{\text{ij}}\right)$$

$$⟹\;\pi_{\delta }^{q}=\pi_{{₳}_{{Ϧ}_{\text{ij}}}^{+}}^{q}$$

$$⟹\;q-\text{RON}\;S_{f}\;\text{WA}\;\left({₳}_{{Ϧ}_{11}},{₳}_{{Ϧ}_{12}},\ldots .,{₳}_{{Ϧ}_{\text{nm}}}\right)={₳}_{{Ϧ}_{\text{ij}}}^{+}$$

iii. sIf S (δ) = S $\left({₳}_{{Ϧ}_{\text{ij}}}^{-}\right)$, then

$${Ʈ}_{\delta }^{q}\;-\;{ƚ}_{\delta }^{q}-{Ƒ}_{\delta }^{q}+\left(\frac{e^{{Ʈ}_{\delta }^{q}-{ƚ}_{\delta }^{q}-{Ƒ}_{\delta }^{q}}}{e^{{Ʈ}_{\delta }^{q}-{ƚ}_{\delta }^{q}-{Ƒ}_{\delta }^{q}}+1}-\frac{1}{2}\right)\pi_{\delta }^{q}$$

$$={\left(\min_{j}\min_{i}\left\{{Ʈ}_{\text{ij}}\right\}\right)}^{q}-{\left(\max_{j}\max_{i}\left\{{ƚ}_{\text{ij}}\right\}\right)}^{q}-{\left(\max_{j}\max_{i}\left\{{Ƒ}_{\text{ij}}\right\}\right)}^{q}+\left(\frac{e^{\;{\left(\min_{j}\min_{i}\left\{{Ʈ}_{\text{ij}}\right\}\right)}^{q}-\;{\left(\max_{j}\max_{i}\left\{{ƚ}_{\text{ij}}\right\}\right)}^{q}-\;{\left(\max_{j}\max_{i}\left\{{Ƒ}_{\text{ij}}\right\}\right)}^{q}}}{e^{\;{\left(\min_{j}\min_{i}\left\{{Ʈ}_{\text{ij}}\right\}\right)}^{q}-\;{\left(\max_{j}\max_{i}\left\{{ƚ}_{\text{ij}}\right\}\right)}^{q}-{\left(\max_{j}\max_{i}\left\{{Ƒ}_{\text{ij}}\right\}\right)}^{q}}+1}-\frac{1}{2}\right)\pi_{{₳}_{{Ϧ}_{\text{ij}}}^{-}}^{q}$$

then by the above inequalities, we get

$$⟹\;{Ʈ}_{\delta }=\min_{j}\min_{i}\left({Ʈ}_{\text{ij}}\right),{ƚ}_{\delta }=\max_{j}\max_{i}\left({ƚ}_{\text{ij}}\right),{Ƒ}_{\delta }=\max_{j}\max_{i}\left({Ƒ}_{\text{ij}}\right)$$

$$⟹\;\pi_{\delta }^{q}=\pi_{{₳}_{{Ϧ}_{\text{ij}}}^{-}}^{q}$$

$$⟹\;q-\text{RON}\;S_{f}\;\text{WA}\;\left({₳}_{{Ϧ}_{11}},{₳}_{{Ϧ}_{12}},\ldots .,{₳}_{{Ϧ}_{\text{nm}}}\right)={₳}_{{Ϧ}_{\text{ij}}}^{-}$$

Hence

$${₳}_{{Ϧ}_{\text{ij}}}^{-}\leq q-\text{RON}S_{f}\text{WA}\;\left({₳}_{{Ϧ}_{11}},{₳}_{{Ϧ}_{12}},\ldots .,{₳}_{{Ϧ}_{\text{nm}}}\right)\leq {₳}_{{Ϧ}_{\text{ij}}}^{+}\text{.}$$

Proof**(b),(c),(d):** Straightforward as above.

Property 3 (Ϻonotonicity):

a. If ${₦}_{{Ϧ}_{\text{ij}}}$ = $\left({₩}_{\text{ij}},{₲}_{\text{ij}},{\;₱}_{\text{ij}}\right)$ be a q-RON $S_{f}N_{s}$ such that ${Ʈ}_{\text{ij}}\leq {₩}_{\text{ij}},{ƚ}_{\text{ij}}\geq {₲}_{\text{ij}}$, ${Ƒ}_{\text{ij}}\geq {\;₱}_{\text{ij}}$ then q-RON $S_{f}$ WA $\left({₳}_{{Ϧ}_{11}},{₳}_{{Ϧ}_{12}},\ldots .,{₳}_{{Ϧ}_{\text{nm}}}\right)$ ≤ q-RONF $S_{f}$ WA $\left({₦}_{{Ϧ}_{11}},{₦}_{{Ϧ}_{12}},\ldots .,{₦}_{{Ϧ}_{\text{nm}}}\right)$.
b. If ${₦}_{{Ϧ}_{\text{ij}}}$ = $\left({₩}_{\text{ij}},{₲}_{\text{ij}},{\;₱}_{\text{ij}}\right)$ be a q-RON $S_{f}N_{s}$ such that ${Ʈ}_{\text{ij}}\leq {₩}_{\text{ij}},{ƚ}_{\text{ij}}\geq {₲}_{\text{ij}}$, ${Ƒ}_{\text{ij}}\geq {\;₱}_{\text{ij}}$ then q-RON $S_{f}O$ WA $\left({₳}_{{Ϧ}_{11}},{₳}_{{Ϧ}_{12}},\ldots .,{₳}_{{Ϧ}_{\text{nm}}}\right)$ ≤ q-RONF $S_{f}O$ WA $\left({₦}_{{Ϧ}_{11}},{₦}_{{Ϧ}_{12}},\ldots .,{₦}_{{Ϧ}_{\text{nm}}}\right)$.
c. If ${₦}_{{Ϧ}_{\text{ij}}}$ = $\left({₩}_{\text{ij}},{₲}_{\text{ij}},{\;₱}_{\text{ij}}\right)$ be a q-RON $S_{f}N_{s}$ such that ${Ʈ}_{\text{ij}}\leq {₩}_{\text{ij}},{ƚ}_{\text{ij}}\geq {₲}_{\text{ij}}$, ${Ƒ}_{\text{ij}}\geq {\;₱}_{\text{ij}}$ then q-RON $S_{f}$ WG $\left({₳}_{{Ϧ}_{11}},{₳}_{{Ϧ}_{12}},\ldots .,{₳}_{{Ϧ}_{\text{nm}}}\right)$ ≤ q-RONF $S_{f}$ WG $\left({₦}_{{Ϧ}_{11}},{₦}_{{Ϧ}_{12}},\ldots .,{₦}_{{Ϧ}_{\text{nm}}}\right)$.
d. If ${₦}_{{Ϧ}_{\text{ij}}}$ = $\left({₩}_{\text{ij}},{₲}_{\text{ij}},{\;₱}_{\text{ij}}\right)$ be a q-RON $S_{f}N_{s}$ such that ${Ʈ}_{\text{ij}}\leq {₩}_{\text{ij}},{ƚ}_{\text{ij}}\geq {₲}_{\text{ij}}$, ${Ƒ}_{\text{ij}}\geq {\;₱}_{\text{ij}}$ then q-RON $S_{f}O$ WG $\left({₳}_{{Ϧ}_{11}},{₳}_{{Ϧ}_{12}},\ldots .,{₳}_{{Ϧ}_{\text{nm}}}\right)$ ≤ q-RONF $S_{f}O$ WG $\left({₦}_{{Ϧ}_{11}},{₦}_{{Ϧ}_{12}},\ldots .,{₦}_{{Ϧ}_{\text{nm}}}\right)$

Proof**(a):** Since ${{Ʈ}_{\text{ij}}\leq ₩}_{\text{ij}},{{ƚ}_{\text{ij}}\geq ₲}_{\text{ij}}\;\text{and}{\;{Ƒ}_{\text{ij}}\geq ₱}_{\text{ij}}$, then

$$\Rightarrow \;{{Ʈ}_{\text{ij}}\leq ₩}_{\text{ij}}\;\Rightarrow \;1\;-\;{₩}_{\text{ij}}\leq 1-\;{Ʈ}_{\text{ij}}\;\Rightarrow \;1\;-\;{₩}_{ij}^{q}\leq 1-\;{Ʈ}_{ij}^{q}$$

$$\Rightarrow \;\Pi_{j=1}^{m}{\left(\Pi_{i=1}^{n}{\left(1-{₩}_{ij}^{q}\right)}^{\omega_{i}}\right)}^{\upsilon_{j}}\leq \Pi_{j=1}^{m}{\left(\Pi_{i=1}^{n}{\left(1-{Ʈ}_{ij}^{q}\right)}^{\omega_{i}}\right)}^{\upsilon_{j}}$$

$$\Rightarrow \;1-\Pi_{j=1}^{m}{\left(\Pi_{i=1}^{n}{\left(1-{Ʈ}_{ij}^{q}\right)}^{\omega_{i}}\right)}^{\upsilon_{j}}\leq 1-\Pi_{j=1}^{m}{\left(\Pi_{i=1}^{n}{\left(1-{₩}_{ij}^{q}\right)}^{\omega_{i}}\right)}^{\upsilon_{j}}$$

$$\Rightarrow \;\sqrt[{q}]{\;1-\Pi_{j=1}^{m}{\left(\Pi_{i=1}^{n}{\left(1-{Ʈ}_{ij}^{q}\right)}^{\omega_{i}}\right)}^{\upsilon_{j}}}\leq \sqrt[{q}]{1-\Pi_{j=1}^{m}{\left(\Pi_{i=1}^{n}{\left(1-{₩}_{ij}^{q}\right)}^{\omega_{i}}\right)}^{\upsilon_{j}}}$$

next ${{ƚ}_{\text{ij}}\geq ₲}_{\text{ij}}$

$$\Rightarrow \;\Pi_{i=1}^{n}{\left({ƚ}_{\text{ij}}\right)}^{\omega_{i}}\geq \Pi_{i=1}^{n}{\left({₲}_{\text{ij}}\right)}^{\omega_{i}}$$

$$\Rightarrow \;\Pi_{j=1}^{m}{\left(\Pi_{i=1}^{n}{\left({ƚ}_{\text{ij}}\right)}^{\omega_{i}}\right)}^{\upsilon_{j}}\geq \Pi_{j=1}^{m}{\left(\Pi_{i=1}^{n}{\left({₲}_{\text{ij}}\right)}^{\omega_{i}}\right)}^{\upsilon_{j}}$$

and ${\;{Ƒ}_{\text{ij}}\geq ₱}_{\text{ij}}$

$$\Rightarrow \;\Pi_{i=1}^{n}{\left({Ƒ}_{\text{ij}}\right)}^{\omega_{i}}\geq \Pi_{i=1}^{n}{\left({₱}_{\text{ij}}\right)}^{\omega_{i}}$$

$$\Rightarrow \;\Pi_{j=1}^{m}{\left(\Pi_{i=1}^{n}{\left({Ƒ}_{\text{ij}}\right)}^{\omega_{i}}\right)}^{\upsilon_{j}}\geq \Pi_{j=1}^{m}{\left(\Pi_{i=1}^{n}{\left({₱}_{\text{ij}}\right)}^{\omega_{i}}\right)}^{\upsilon_{j}}$$

Suppose that $\delta_{₳}$ = q-RON $S_{f}$ WA $\left({₳}_{{Ϧ}_{11}},{₳}_{{Ϧ}_{12}},\ldots .,{₳}_{{Ϧ}_{\text{nm}}}\right)$ = $\left({Ʈ}_{\delta_{₳}},{ƚ}_{\delta_{₳}},{Ƒ}_{\delta_{₳}}\right)$ and

$$\delta_{₦}=q-\text{RON}\;S_{f}\;\text{WA}\;\left({₦}_{{Ϧ}_{11}},{₦}_{{Ϧ}_{12}},\ldots .,{₦}_{{Ϧ}_{\text{nm}}}\right)=\left({₩}_{\delta_{₦}},{₲}_{\delta_{₦}},{₱}_{\delta_{₦}}\right)$$

Now, from the above equation, we have

$${{Ʈ}_{\text{ij}}\leq ₩}_{\text{ij}},{{ƚ}_{\text{ij}}\geq ₲}_{\text{ij}}\;\text{and}{\;{Ƒ}_{\text{ij}}\geq ₱}_{i\;j}$$

S $\left(\delta_{₳}\right)\leq$ S $\left(\delta_{₦}\right)$, by using the score value

The following cases are encounter:

I. By the comparison of two q-ROP soft numbers, if S $\left(\delta_{₳}\right)<$ S $\left(\delta_{₦}\right)$, then

$$q-\text{RON}\;S_{f}\;\text{WA}\;\left({₳}_{{Ϧ}_{11}},{₳}_{{Ϧ}_{12}},\ldots .,{₳}_{{Ϧ}_{\text{nm}}}\right)<\;q-\text{RON}S_{f}\text{WA}\;\left({₦}_{{Ϧ}_{11}},{₦}_{{Ϧ}_{12}},\ldots .,{₦}_{{Ϧ}_{\text{nm}}}\right)\text{.}$$

II. If S $\left(\delta_{₳}\right)=$ S $\left(\delta_{R}\right)$, where

$$S\;\left(\delta_{₳}\right)={Ʈ}_{\delta_{₳}}^{q}\;-\;{ƚ}_{\delta_{₳}}^{q}-{Ƒ}_{\delta_{₳}}^{q}+\left(\frac{e^{{Ʈ}_{\delta_{₳}}^{q}-{ƚ}_{\delta_{₳}}^{q}-{Ƒ}_{\delta_{₳}}^{q}}}{e^{{Ʈ}_{\delta_{₳}}^{q}-{ƚ}_{\delta_{₳}}^{q}-{Ƒ}_{\delta_{₳}}^{q}}+1}-\frac{1}{2}\right)\pi_{\delta_{₳}}^{q}$$

$$S\;\left(\delta_{₦}\right)={Ʈ}_{\delta_{₦}}^{q}\;-\;{ƚ}_{\delta_{₦}}^{q}-{Ƒ}_{\delta_{₦}}^{q}+\left(\frac{e^{{Ʈ}_{\delta_{₦}}^{q}-{ƚ}_{\delta_{₦}}^{q}-{Ƒ}_{\delta_{₦}}^{q}}}{e^{{Ʈ}_{\delta_{₦}}^{q}-{ƚ}_{\delta_{₦}}^{q}-{Ƒ}_{\delta_{₦}}^{q}}+1}-\frac{1}{2}\right)\pi_{\delta_{₦}}^{q}$$

We have, ${{Ʈ}_{\delta_{₳}}=₩}_{\delta_{₦}},{{ƚ}_{\delta_{₳}}=₲}_{\delta_{₦}}\;\text{and}{\;{Ƒ}_{\delta_{₳}}=₱}_{\delta_{₦}}$. Hence

$$\Rightarrow \;\pi_{\delta_{₳}}^{q}=\pi_{\delta_{₦}}^{q}$$

$$\Rightarrow \;\left({Ʈ}_{\delta_{₳}},{ƚ}_{\delta_{₳}},{Ƒ}_{\delta_{₳}}\right)=\left({₩}_{\delta_{₦}},{₲}_{\delta_{₦}},{₱}_{\delta_{₦}}\right)$$

Proved that q-RON $S_{f}$ WA $\left({₳}_{{Ϧ}_{11}},{₳}_{{Ϧ}_{12}},\ldots .,{₳}_{{Ϧ}_{\text{nm}}}\right)<\;q-\text{RON}S_{f}\text{WA}\;\left({₦}_{{Ϧ}_{11}},{₦}_{{Ϧ}_{12}},\ldots .,{₦}_{{Ϧ}_{\text{nm}}}\right)$.

Proof**(b),(c),(d):** Straightforward as above.

Property 4 (ShiftInvariance):

a. If ${₦}_{\ddot{e}=}\left({₩}_{\text{ij}},{₲}_{\text{ij}},{\;₱}_{\text{ij}}\right)$ is q-RON $S_{f}N_{s}$, then q-RON $S_{f}$ WA $\left({₳}_{{Ϧ}_{11}}⊕{₦}_{Ϧ},{₳}_{{Ϧ}_{12}}⊕{₦}_{Ϧ},\ldots .,{₳}_{{Ϧ}_{\text{nm}}}⊕{₦}_{Ϧ}\right)=$ q-RON $S_{f}$ WA $\left({₳}_{{Ϧ}_{11}},{₳}_{{Ϧ}_{12}},\ldots .,{₳}_{{Ϧ}_{\text{nm}}}\right)⊕{₦}_{Ϧ}$.
b. If ${₦}_{\ddot{e}=}\left({₩}_{\text{ij}},{₲}_{\text{ij}},{\;₱}_{\text{ij}}\right)$ is q-RON $S_{f}N_{s}$, then q-RON $S_{f}O$ WA $\left({₳}_{{Ϧ}_{11}}⊕{₦}_{Ϧ},{₳}_{{Ϧ}_{12}}⊕{₦}_{Ϧ},\ldots .,{₳}_{{Ϧ}_{\text{nm}}}⊕{₦}_{Ϧ}\right)=$ q-RON $S_{f}O$ WA $\left({₳}_{{Ϧ}_{11}},{₳}_{{Ϧ}_{12}},\ldots .,{₳}_{{Ϧ}_{\text{nm}}}\right)⊕{₦}_{Ϧ}$.
c. If ${₦}_{\ddot{e}=}\left({₩}_{\text{ij}},{₲}_{\text{ij}},{\;₱}_{\text{ij}}\right)$ is q-RON $S_{f}N_{s}$, then q-RON $S_{f}$ WG $\left({₳}_{{Ϧ}_{11}}⊕{₦}_{Ϧ},{₳}_{{Ϧ}_{12}}⊕{₦}_{Ϧ},\ldots .,{₳}_{{Ϧ}_{\text{nm}}}⊕{₦}_{Ϧ}\right)=$ q-RON $S_{f}$ WG $\left({₳}_{{Ϧ}_{11}},{₳}_{{Ϧ}_{12}},\ldots .,{₳}_{{Ϧ}_{\text{nm}}}\right)⊕{₦}_{Ϧ}$.
d. If ${₦}_{\ddot{e}=}\left({₩}_{\text{ij}},{₲}_{\text{ij}},{\;₱}_{\text{ij}}\right)$ is q-RON $S_{f}N_{s}$, then q-RON $S_{f}O$ WG $\left({₳}_{{Ϧ}_{11}}⊕{₦}_{Ϧ},{₳}_{{Ϧ}_{12}}⊕{₦}_{Ϧ},\ldots .,{₳}_{{Ϧ}_{\text{nm}}}⊕{₦}_{Ϧ}\right)=$ q-RON $S_{f}$ OWG $\left({₳}_{{Ϧ}_{11}},{₳}_{{Ϧ}_{12}},\ldots .,{₳}_{{Ϧ}_{\text{nm}}}\right)⊕{₦}_{Ϧ}$

Proof**(a):** Since ${₦}_{Ϧ}=\left(₩,₲,₱\right)$ and ${₳}_{{Ϧ}_{\text{ij}}}$ = $\left({Ʈ}_{{Ϧ}_{\text{ij}}},{ƚ}_{{Ϧ}_{\text{ij}}},{Ƒ}_{{Ϧ}_{\text{ij}}}\right)$ are the q-ROP soft numbers, so

$${₳}_{{Ϧ}_{\text{ij}}}⊕{₦}_{Ϧ=}\left(\sqrt[{q}]{\left(1-{Ʈ}_{ij}^{q}\right)\left(1-{₩}^{q}\right)},{ƚ}_{ij}^{q}₲,{Ƒ}_{ij}^{q}₱\right).\;\text{Therefore,}$$

$$q-\text{RON}\;S_{f}\;\text{WA}\;\left({₳}_{{Ϧ}_{11}}⊕{₦}_{Ϧ},{₳}_{{Ϧ}_{12}⊕}{₦}_{Ϧ},\ldots .,{₳}_{{Ϧ}_{\text{nm}}}⊕{₦}_{Ϧ}\right)={⊕}_{j=1}^{m}\upsilon_{j}\left({⊕}_{i=1}^{n}\omega_{i}\left({₳}_{{Ϧ}_{\text{ij}}}⊕{₦}_{Ϧ}\right)\right)$$

$$=\left(\sqrt[{q}]{1-\Pi_{j=1}^{m}{\left(\Pi_{i=1}^{n}{\left(1-{Ʈ}_{ij}^{q}\right)}^{\omega_{i}}{\left(1-{₩}^{q}\right)}^{\omega_{i}}\right)}^{\upsilon_{j}}},\Pi_{j=1}^{m}{\left(\Pi_{i=1}^{n}{ƚ}_{ij}^{\omega_{i}}{₲}^{\omega_{i}}\right)}^{\upsilon_{j}},\Pi_{j=1}^{m}{\left(\Pi_{i=1}^{n}{Ƒ}_{ij}^{\omega_{i}}{₱}^{\omega_{i}}\right)}^{\upsilon_{j}}\right)$$

$$=\left(\sqrt[{q}]{1-{\left(1-{₩}^{q}\right)\Pi }_{j=1}^{m}{\left(\Pi_{i=1}^{n}{\left(1-{Ʈ}_{ij}^{q}\right)}^{\omega_{i}}\right)}^{\upsilon_{j}}},₲\Pi_{j=1}^{m}{\left(\Pi_{i=1}^{n}{ƚ}_{ij}^{\omega_{i}}\right)}^{\upsilon_{j}},{₱\Pi }_{j=1}^{m}{\left(\Pi_{i=1}^{n}{Ƒ}_{ij}^{\omega_{i}}\right)}^{\upsilon_{j}}\right)$$

$$=\left(\sqrt[{q}]{1-\Pi_{j=1}^{m}{\left(\Pi_{i=1}^{n}{\left(1-{Ʈ}_{ij}^{q}\right)}^{\omega_{i}}\right)}^{\upsilon_{j}}},\Pi_{j=1}^{m}{\left(\Pi_{i=1}^{n}{ƚ}_{ij}^{\omega_{i}}\right)}^{\upsilon_{j}},\Pi_{j=1}^{m}{\left(\Pi_{i=1}^{n}{Ƒ}_{ij}^{\omega_{i}}\right)}^{\upsilon_{j}}\right)⊕\left(₩,₲,₱\right)$$

$$q-\text{RON}\;S_{f}\;\text{WA}\;\left({₳}_{{Ϧ}_{11}},{₳}_{{Ϧ}_{12}},\ldots .,{₳}_{{Ϧ}_{\text{nm}}}\right)⊕{₦}_{Ϧ}\text{.}$$

Proof**(b),(c),(d):** Straightforward as above.

Property 5 (Homogeneity):

a. If λ ≥ 0, then q-RON $S_{f}$ WA $\left(\lambda {₳}_{{Ϧ}_{11}},{\lambda ₳}_{{Ϧ}_{12}},\ldots .,{\lambda ₳}_{{Ϧ}_{\text{nm}}}\right)$ = λ q-RON $S_{f}$ WA $\left({₳}_{{Ϧ}_{11}},{₳}_{{Ϧ}_{12}},\ldots .,{₳}_{{Ϧ}_{\text{nm}}}\right)$.
b. If λ ≥ 0, then q-RON $S_{f}O$ WA $\left(\lambda {₳}_{{Ϧ}_{11}},{\lambda ₳}_{{Ϧ}_{12}},\ldots .,{\lambda ₳}_{{Ϧ}_{\text{nm}}}\right)$ = λ q-RON $S_{f}O$ WA $\left({₳}_{{Ϧ}_{11}},{₳}_{{Ϧ}_{12}},\ldots .,{₳}_{{Ϧ}_{\text{nm}}}\right)$.
c. If λ ≥ 0, then q-RON $S_{f}$ WG $\left(\lambda {₳}_{{Ϧ}_{11}},{\lambda ₳}_{{Ϧ}_{12}},\ldots .,{\lambda ₳}_{{Ϧ}_{\text{nm}}}\right)$ = λ q-RON $S_{f}$ WG $\left({₳}_{{Ϧ}_{11}},{₳}_{{Ϧ}_{12}},\ldots .,{₳}_{{Ϧ}_{\text{nm}}}\right)$.
d. If λ ≥ 0, then q-RON $S_{f}O$ WG $\left(\lambda {₳}_{{Ϧ}_{11}},{\lambda ₳}_{{Ϧ}_{12}},\ldots .,{\lambda ₳}_{{Ϧ}_{\text{nm}}}\right)$ = λ q-RON $S_{f}O$ WG $\left({₳}_{{Ϧ}_{11}},{₳}_{{Ϧ}_{12}},\ldots .,{₳}_{{Ϧ}_{\text{nm}}}\right)$.

Proof**(a):** Assume that λ ≥ 0 and let ${₳}_{{Ϧ}_{\text{ij}}}$ = $\left({Ʈ}_{{Ϧ}_{\text{ij}}},{ƚ}_{{Ϧ}_{\text{ij}}},{Ƒ}_{{Ϧ}_{\text{ij}}}\right)$ be a q-RON $S_{f}N_{s}\text{,}$ then

$$\lambda ₳=\left(\sqrt[{q}]{1-{\left(1-{Ʈ}_{ij}^{q}\right)}^{\lambda }},{ƚ}_{ij}^{q},{Ƒ}_{ij}^{q}\right)$$

$$q-RONS_{f}WA\;\left(\lambda {₳}_{{Ϧ}_{11}},\lambda {₳}_{{Ϧ}_{12}},\ldots .,{\lambda ₳}_{{Ϧ}_{nm}}\right)=\left(\begin{array}{c} \sqrt[{q}]{1-{\left(\prod_{j=1}^{m}{\left(\prod_{i=1}^{n}{\left(1-{Ʈ}_{ij}^{q}\right)}^{\omega_{i}}\right)}^{\upsilon_{j}}\right)}^{\lambda }},\\ {\left(\Pi_{j=1}^{m}{\left(\Pi_{i=1}^{n}{ƚ}_{ij}^{\omega_{i}}\right)}^{\upsilon_{j}}\right)}^{\lambda },\\ {\left(\Pi_{j=1}^{m}{\left(\Pi_{i=1}^{n}{Ƒ}_{ij}^{\omega_{i}}\right)}^{\upsilon_{j}}\right)}^{\lambda } \end{array}\right)$$

$$=\lambda \;q-RONS_{f}WA\;\left({₳}_{{Ϧ}_{11}},{₳}_{{Ϧ}_{12}},\ldots .,{₳}_{{Ϧ}_{nm}}\right)\text{.}$$

Proof**(b),(c),(d):** Straightforward as above.

## 4 Mathematical model based on q-RON soft information for MCGDM

In this section, we consider a mathematical model based on the proposed structure for solving the MCDM problem. DM is a pre-planned procedure for identifying and selecting the optimal option from a variety of alternatives.

For this let the set of alternatives represented by Ą = {ą₁, ą₂, …...ą_l_} and set of parameters Ĉ = {ĉ₁, ĉ₂ …..., ĉ_m_}. After evaluating each alternative to their corresponding parameters, expert give their assessment in the form of from ${₳}_{{\ddot{e}}_{ij}}$ = $\left({Ʈ}_{ij},{ƚ}_{ij},{Ƒ}_{ij}\right)$ with WV ω = ${\left(\omega ₁,\omega 2,.....,\omega n\right)}^{T}$ for expert x_i_ having $\sum_{i=1}^{n}\omega_{i}=1$ and for parameters $\sum_{i=1}^{n}\upsilon_{i}=1\text{.}$.

**Fig. 3:**
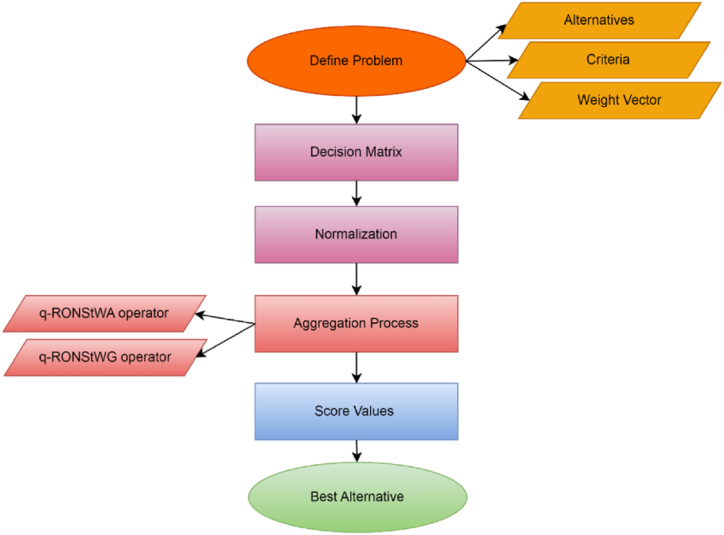
Flow chart of the proposed model problem through aggregation operators.

Where M = ${\left[{₳}_{{Ϧ}_{ij}}\right]}_{m\times n\;}$ expressed the collected information from professional experts and then applied a developed model on the decision matrix to get an aggregated $q-\text{RON}S_{f}N_{s}$ for each alternative against their parameters. Finally, we compute the score value of aggregated data and rank the alternatives for optimum results. The overall framework and sequential procedure for the proposed methodology are outlined as follows: Fig. 3 represent the flow chat of proposed model.

**Table:** 

Algorithm: Using $q-\mathrm{RON}S_{f}\text{WA}$ and $q-\mathrm{RON}S_{f}\text{WG}$, to deal MADM problem.
Input:
Step 1. Established a decision matrix M = ${\left[{₳}_{{Ϧ}_{ij}}\right]}_{m\times n\;}\text{.}$
Step 2. Normalize decision matrix M = ${\left[{₳}_{{Ϧ}_{ij}}\right]}_{m\times n\;}$
$$P_{ij}=\left\{ \begin{array}{c} {₳}_{{Ϧ}_{\text{ij}}}^{C}:\mathrm{for}\;\mathrm{cost}\;\mathrm{type}\;\mathrm{parameter}\\ {₳}_{{Ϧ}_{\text{ij}}}\mathrm{for}\;\mathrm{benefit}\;\mathrm{type}\;\mathrm{parameter}\; \end{array}\right.$$
where ${₳}_{{Ϧ}_{\text{ij}}}^{C}$ represent the complement of ${₳}_{{Ϧ}_{\text{ij}}}\text{.}$
Step 3. To evaluate the decision matrix M = ${\left[{₳}_{{Ϧ}_{ij}}\right]}_{m\times n\;}$ using q-RON $S_{f}$ WA and q-RON $S_{f}$ WG operators.
Step 4. To aggregate data compute score values.
Step 5. For optimum results rank the alternatives using score values.
Output:
The choice will be determined by the alternative that achieves the highest score.

### 4.1 Decision making problem for the selection of employees of a particular position in a real estate company

In real-life real estate industry play an important role for the development of country by encompasses a wide-range of activities related to buying, selling, leasing and development of properties such as agriculture, residential, commercial and industrial land. Real estate industry affected by various thing such as technology, laws and economy of a country. Also, it plays an important role in urban development, job creation, wealth generation and economic growth. Here we consider aa example of a typical decision-making challenge in this field. That company not only value the technical skill of the candidates (employee) for a particular position in a real estate company but also their soft skills like commination, adaptability and leadership, which are crucial for managing team and dealing with clients effectively. For this scenario, we utilized our proposed model of q-RONSS which characterized the problem by tripled memberships with attributes and qualification required for the job including both aspect of qualitative and quantitative. To assess their suitability for the particular position in a company, each candidate must be evaluated their profile using the proposed model.

• Representation of candidate's profile: The qualification, experience related to the field, communication skills and general aptitude of each candidate are representing by utilizing the proposed model ($q-RONS_{f}S$).
• Assessment of suitability: Utilizing q-rung orthopair neutrosophic soft operators, the attributes of each candidate can be combining and compare by the company with the requirement of particular position.
• Decision-making: Based on $q-RONS_{f}$ evaluation, the company can make informed decision related to candidate selection.

Adaptability of changing requirement: To ensuring the continued effectiveness of the selection process of employ, the company need to updated and improve the proposed model according to feedback and evolving business requirement.

### 4.2 Application of proposed model through numerical example

In this section, we consider a numerical example related to the personal selection problem of employees for a particular position in a real estate company namely “Makaan Solution”. But this selection not only value the technical skill of the employee but also their soft skills like commination, adaptability and leadership, which are crucial for managing team and dealing with clients effectively. For this scenario, we utilized our proposed model of q-RONSS which characterized the problem by tripled memberships with attributes and qualification required for the job including both aspect of qualitative and quantitative. After the preliminary elimination process, four candidates $\left\{\times_{1},\times_{2},\times_{3},\times_{4}\right\}$ which express the alternatives, are entering the final round of interviews. Which are evaluated by a team of four expert decision-makers:

**Group of experts**:

$${\text{DM}}_{1}=\text{Rizwan}\;\text{chema,}$$

$${\text{DM}}_{2}=\text{Sana}\;\text{ch}.,$$

$${\text{DM}}_{3}=\text{Nimra}\;\text{ch}.,$$

$${\text{DM}}_{4}=\text{Salman}\;\text{chema}$$

having weight vector ω = ${\left(0.20,0.25,0.27,0.28\right)}^{T}$. To asses their suitability for the particular position in a company, each candidate must be evaluated their profile using the proposed model on the bases on criteria

**Fig. 4:**
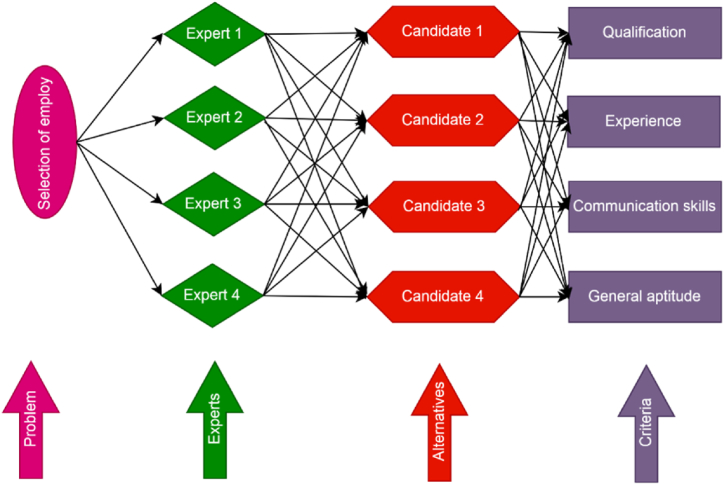
Decision tree for site selection of best employ for the company.

**Fig. 5:**
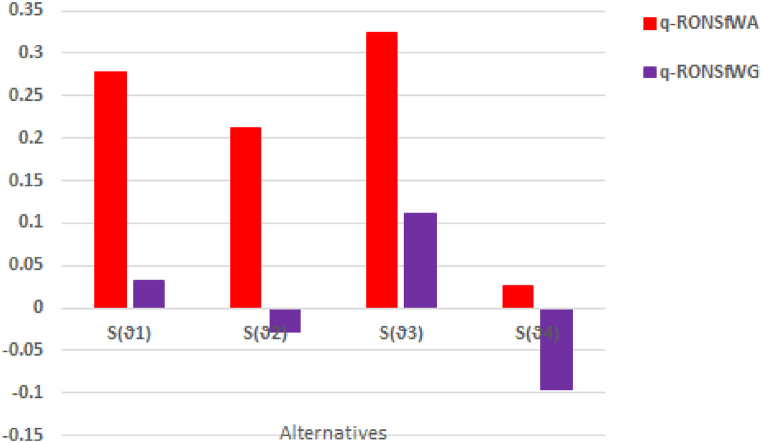
Graphical representation of score values $q-\text{RON}S_{f}\text{WA}$ and $q-\text{RON}S_{f}\text{WG}$.

**Criteria**:

$y_{1}=\text{Qualification}$: The expert evaluates the education background of candidate, that he possesses the necessary academic qualification required for the position in the real estate company.
$y_{2}=\text{Experience}\;\text{related}\;\text{to}\;\text{the}\;\text{field}$: Candidate must have a strong track record of success and demonstrated expertise in the real estate sector.
$y_{3}=\text{Communication}\;\text{skills}$: Strong communication skills are the main factor of real estate sector. So the candidate must have the ability to convey information clearly, listen actively, negotiate effectively, and built rapport with other.
$y_{4}=\text{General}\;\text{aptitude}$: Candidate with a well-rounded skill, decision-making capabilities, adaptability of new situations and positive attitude are often considered suitable for various role in a real estate company.

having weight vector υ = ${\left(0.5,0.16,0.14,0.20\right)}^{T}$. For the selection of the best employee for the company, a step-wise algorithm is constructed. Fig. 4 express a decision tree for the personal selection problem of employees (see The graphical image of score values is shown in Fig. 5.).

**By using q-RONSWAO**:

**Step 1:** Established M = ${\left[{₳}_{{Ϧ}_{\text{ij}}}\right]}_{m\times n\;}$, shown in Table 4, Table 5, Table 6, Table 7, respectively (see Table 8).

**Table 4:** Rating values $\text{for}\;\text{alternative}\;\times_{1}$ provided by a decision maker.

Experts	$y_{1}$	$y_{2}$	$y_{3}$	$y_{4}$
${\text{DM}}_{1}$	(.8,.3,.1)	(.9,.1,.2)	(.8,.6,.3)	(.7,.4,.2)
${\text{DM}}_{2}$	(0.7,0.4,0.2)	(0.6,0.3,0.3)	(0.7,0.4,0.3)	(0.8,0.2,0.4)
${\text{DM}}_{3}$	(0.5,0.4,0.2)	(0.2,0.1,0.3)	(0.3,0.4,0.3)	(0.5,0.3,0.7)
${\text{DM}}_{4}$	(0.4,0.1,0.3)	(0.3,0.6,0.2)	(0.4,0.1,0.2)	(0.1,0.1,0.1)

**Table 5:** Rating values $\text{for}\;\text{alternative}\;\times_{2}$ provided by a decision maker.

Experts	$y_{1}$	$y_{2}$	$y_{3}$	$y_{4}$
${\text{DM}}_{1}$	(.8,.4,.2)	(.9,.1,.5)	(.8,.4,.3)	(.1,.3,.2)
${\text{DM}}_{2}$	(0.2,0.1,0.2)	(0.5,0.3,0.3)	(0.5,0.4,0.3)	(0.8,0.2,0.2)
${\text{DM}}_{3}$	(0.3,0.2,0.3)	(0.2,0.5,0.1)	(0.3,0.3,0.3)	(0.2,0.1,0.1)
${\text{DM}}_{4}$	(0.4,0.6,0.1)	(0.3,0.1,0.2)	(0.4,0.1,0.2)	(0.7,0.3,0.4)

**Table 6:** Rating values $\text{for}\;\text{alternative}\;\times_{3}$ provided by a decision maker.

Experts	$y_{1}$	$y_{2}$	$y_{3}$	$y_{4}$
${\text{DM}}_{1}$	(.8,.3,.1)	(.9,.1,.5)	(.8,.6,.3)	(.7,.4,.2)
${\text{DM}}_{2}$	(0.4,0.1,0.3)	(0.7,0.3,0.1)	(0.2,0.1,0.3)	(0.4,0.1,0.4)
${\text{DM}}_{3}$	(0.7,0.4,0.2)	(0.6,0.1,0.3)	(0.7,0.5,0.1)	(0.6,0.3,0.1)
${\text{DM}}_{4}$	(0.3,0.2,0.3)	(0.8,0.6,0.2)	(0.4,0.3,01)	(0.8,0.2,0.4)

**Table 7:** Rating values $\text{for}\;\text{alternative}\;\times_{4}$ provided by a decision maker.

Experts	$y_{1}$	$y_{2}$	$y_{3}$	$y_{4}$
${\text{DM}}_{1}$	(.4,.3,.1)	(.5,.1,.2)	(.1,.1,.3)	(.2,.1,.2)
${\text{DM}}_{2}$	(0.3,0.2,0.4)	(0.4,0.3,0.3)	(0.5,0.4,0.3)	(0.1,0.2,0.4)
${\text{DM}}_{3}$	(0.5,0.5,0.5)	(0.3,0.2,0.3)	(0.3,0.2,0.1)	(0.5,0.3,0.7)
${\text{DM}}_{4}$	(0.1,0.1,0.4)	(0.1,0.1,0.1)	(0.4,0.1,0.2)	(0.4,0.2,0.4)

**Table 8:** Show the Ranking Order using $q-\text{RON}S_{f}\text{WA}$ and $q-\text{RON}S_{f}\text{WG}$ operators.

Operators	Score values				Ranking
	$\vartheta_{1}$	$\vartheta_{2}$	$\vartheta_{3}$	$\vartheta_{4}$	
$q-RONS_{f}\text{WA}$	0.2768	0.2124	0.3233	0.0261	$S\left(\vartheta_{3}\right)\;>\;S\left(\vartheta_{1}\right)\;>S\left(\vartheta_{2}\right)\;>S\left(\vartheta_{4}\right)$
$q-RONS_{f}\text{WG}$	0.0316	−0.0283	0.1109	−0.0951	$S\left(\vartheta_{3}\right)\;>\;S\left(\vartheta_{1}\right)\;>S\left(\vartheta_{2}\right)\;>S\left(\vartheta_{4}\right)$

**Step 2:** All the parameters are the same, so no need for normalization.
**Step 3:** Using the $q-\text{RON}S_{f}\text{WA}$ operator, to evaluate each value of alternative, so we get

$$\vartheta_{1}=\left(0.6394,0.2447,0.2268\right)$$

$$\vartheta_{2}=\left(0.5845,0.2376,0.2031\right)$$

$$\vartheta_{3}=\left(0.6681,0.2284,0.2135\right)$$

$$\vartheta_{4}=\left(0.3790,0.2004,0.2928\right)$$

**Step 4.** To compare each alternative, determine the score value.

$$S\left(\vartheta_{1}\right)=0.2768$$

$$S\left(\vartheta_{2}\right)=0.2124$$

$$S\left(\vartheta_{3}\right)=0.3233$$

$$S\left(\vartheta_{4}\right)=0.0261$$

**Step 5.** To get the best choice, rank the score values.

0.3233>0.2768>0.2124>0.026

$$S\left(\vartheta_{3}\right)\;>\;S\left(\vartheta_{1}\right)\;>S\left(\vartheta_{2}\right)\;>S\left(\vartheta_{4}\right)$$

The above ranking shows that, $\vartheta_{3}$ is the best employ for a particular position in a real estate company, using $q-\text{RON}S_{f}\text{WAO}$.

By using q-RPNSWG operator:

**Step 1:** Similar as above.
**Step 2:** Similar as above.
**Step 3:** Using the $q-\text{RON}S_{f}\text{WG}$ operator, to evaluate each value of alternative, so we get

$$\vartheta_{1}=\left(0.4829,0.3615,0.3393\right)$$

$$\vartheta_{2}=\left(0.3712,0.3816,0.2659\right)$$

$$\vartheta_{3}=\left(0.5404,0.3468,0.2853\right)$$

$$\vartheta_{4}=\left(0.2697,0.3040,0.4080\right)$$

**Step 4.** To compare each alternative, determine the score value.

$$S\left(\vartheta_{1}\right)=0.0316$$

$$S\left(\vartheta_{2}\right)=-0.0283$$

$$S\left(\vartheta_{3}\right)=0.1109$$

$$S\left(\vartheta_{4}\right)=-0.0951$$

**Step 5.** To get the best choice, rank the score values.

0.1109>0.0316>−0.0283>−0.0951

$$S\left(\vartheta_{3}\right)\;>\;S\left(\vartheta_{1}\right)\;>S\left(\vartheta_{2}\right)\;>S\left(\vartheta_{4}\right)$$

The above ranking shows that, $\vartheta_{3}$ is the best employ for a particular position in a real estate company, using $q-\text{RON}S_{f}\text{WG}$ operator.

## 5 Managerial implications

The authenticity and superiority of proposed model two pivotal analyses are conducted, comparison analysis and characteristic analysis with existing approaches, express in this section.

### 5.1 Comparative analysis

**Fig. 6:**
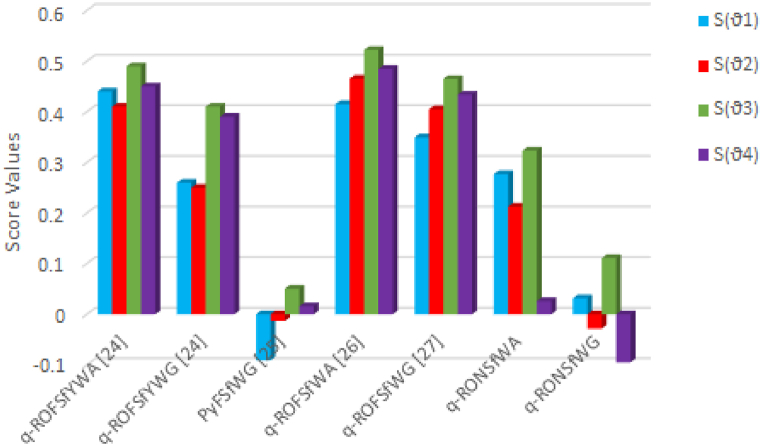
Graphical representation of comparison analysis.

To utilize a variety of proposed operators, we perform comparative analysis of proposed model with existing approaches see ([[56], [57], [58], [59]]). In this section of main goal to show the authenticity of our proposed model, for this we compare the ranking ordered of alternative of suggested model with existing. The ranking order and similarity of best alternative (ϑ₃), emphasizes the exceptional flexibility and comprehensiveness of our proposed technique. This flexibility is particularly given in Table 9 and the comparative analysis is express in Fig. 6.

**Table 9:** Show comparative analysis with existing operators.

Aggregation Operators	Score values				Ranking	Best Alternative
	$\vartheta_{1}$	$\vartheta_{2}$	$\vartheta_{3}$	$\vartheta_{4}$		
$q-\text{ROF}S_{f}\text{YWA}$[24]	0.44	0.41	0.49	0.45	$\vartheta_{3}\;>\;\vartheta_{4}\;>\;\vartheta_{1}>\;\vartheta_{2}$	$\vartheta_{3}$
$q-\text{ROF}S_{f}\text{YWG}$[24]	0.26	0.25	0.41	0.39	$\vartheta_{3}\;>\;\vartheta_{4}\;>\;\vartheta_{1}>\;\vartheta_{2}$	$\vartheta_{3}$
$P_{y}FS_{f}\text{WG}$[25]	−0.0911	−0.0132	0.0505	0.0159	$\vartheta_{3}\;>\;\vartheta_{4}\;>\;\vartheta_{2}>\;\vartheta_{1}$	$\vartheta_{3}$
$q-\text{ROF}S_{f}\text{WA}$[26]	0.4149	0.4654	0.5224	0.4849	$\vartheta_{3}\;>\;\vartheta_{4}\;>\;\vartheta_{1}>\;\vartheta_{2}$	$\vartheta_{3}$
$q-\text{ROF}S_{f}\text{WG}$[27]	0.3493	0.4048	0.4648	0.4337	$\vartheta_{3}\;>\;\vartheta_{4}\;>\;\vartheta_{1}>\;\vartheta_{2}$	$\vartheta_{3}$
$q-\text{RON}S_{f}\text{WA}$ (Proposed)	0.2768	0.2124	0.3233	0.0261	$\vartheta_{3}\;>\;\vartheta_{1}\;>\;\vartheta_{2}>\;\vartheta_{4}$	$\vartheta_{3}$
$q-\text{RON}S_{f}\text{WG}$ (Proposed)	0.0316	−0.0283	0.1109	−0.0951	$\vartheta_{3}\;>\;\vartheta_{1}\;>\;\vartheta_{2}>\;\vartheta_{4}$	$\vartheta_{3}$

### 5.2 Characteristic analysis

**Fig. 7:**
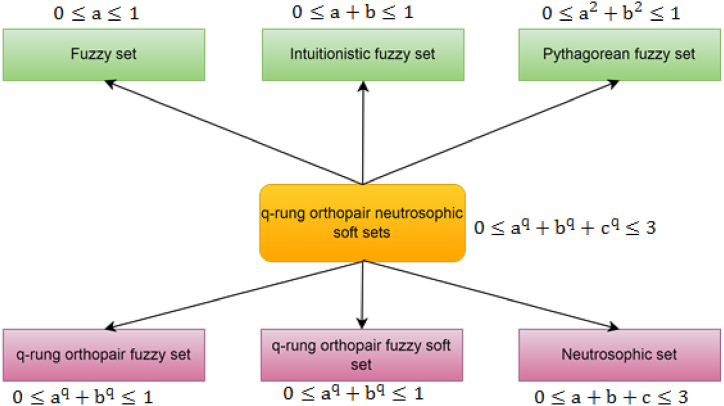
Show the characteristic analysis of proposed model with existing theories.

Through characteristic analysis with various existing theories see ([1,15,21,[11], [36], [43]]) as summarized in Table 10, we explore the superiority of proposed structure. After analysis the existing theories, we conclude that existing approaches deal the uncertainties in MADM problem with MF and non-MF associated with attributes, while it does not address the degree of indeterminacy and also it is important to admit that the existing theories has its specific limitations, which restricts decision-maker to evaluating alternatives within the domain. So, to tackle these limitations we proposed more flexible and free structure by using NS in q-ROFSS and proposed novel structure of q-RONSS, characterized by three parameter, truth, indeterminacy and false membership with attributes provides the more flexible environment to the decision maker by relaxing the domain. This is the logic behind our proposed approach that neutrosophic set handles the situation 0≤μ+η+ν≤3, but failed the condition ($0\leq {\left(\mu \right)}^{q}+{\left(\eta \right)}^{q}+{\left(\nu \right)}^{q}\leq 3$)**.** Fig. 7 Show the characteristic analysis of proposed model with existing theories.

**Table 10:** Characteristic analysis of various theories.

**Theories**	**Truth** **Membership**	**Indeterminacy membership**	**False membership**	**Parameter**	**Domain**
**FS**[1]	✓	✕	✕	✕	0≤a≤1
**IFS**[20]	✓	✕	✓	✕	0≤a+b≤1
**PyFS**[6]	✓	✕	✓	✕	$0\leq a^{2}+b^{2}\leq 1$
**q-ROFS**[14]	✓	✕	✓	✕	$0\leq a^{q}+b^{q}\leq 1\;\left(q\geq 1\right)$
**q-ROF**$S_{t}S$[18]	✓	✕	✓	✓	$0\leq a^{q}+b^{q}\leq 1\;\left(q\geq 1\right)$
**NS**[19]	✓	✓	✓	✕	0≤a+b+c≤3
**[This Paper]**	✓	✓	✓	✓	$0\leq a^{q}+b^{q}+c^{q}\leq 3\;\left(q\geq 1\right)\;)$

### 5.3 Sensitivity analysis

**Fig. 8:**
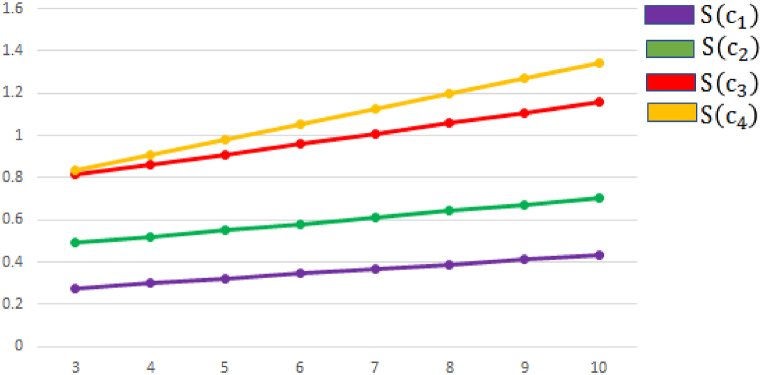
Ranking order of various values of parameter r and q by using $q-\text{RONF}S_{t}\text{WA}$.

**Fig. 9:**
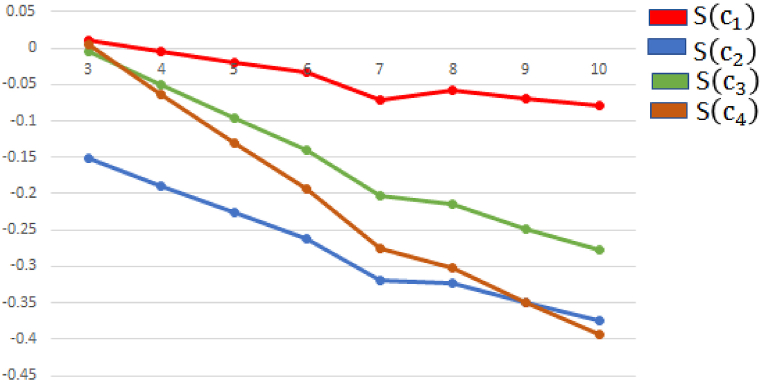
Ranking order of various values of parameter r and q by using $q-\text{RONF}S_{t}\text{WG}$.

In this segment, we address the reliability and influence of various values of parameters $\prime \prime q^{\prime \prime }$ impact the alternatives and the ultimate ranking outcomes by using aggregation operators. Our main goal investigate the effect of “q” on the score of alternatives. To understand the relation between parameters variation and decision-outcome, we utilize $q-\text{RONF}S_{t}\text{WA}$ operator on the various values of q = 3, 4, …..,10 as shown in Table 11. If we increase the value of parameter q and the ranking order of alternative remain consistent $\vartheta_{3}\;>\;\vartheta_{1}\;>\;\vartheta_{2}>\;\vartheta^{4}$ and the score values of alternatives increase, which show a strong consistency in decision outcomes. The behaver of alternatives for different values of “q” by using $q-\text{RONF}S_{t}\text{WA}$ operator is presented in Fig. 8. If we utilize $q-\text{RONF}S_{t}$ WG operator by increase the value of parameter q = 3, 4, …,10, the ranking order of alternative $\vartheta_{3}\;>\;\vartheta_{1}\;>\;\vartheta_{2}>\;\vartheta^{4}$ and best alternative $\vartheta_{3}$ remain consistent and the score values of alternatives decrease, which show a strong consistency in decision outcomes. The behaver of alternatives for different values of “q” by using $q-\text{RONF}S_{t}\text{WG}$ operator is presented in Fig. 9.

**Table 11:** Sorting alternatives according to their respective parameter q values using $q-\text{RONF}S_{t}\text{WA}$ and $q-\text{RONF}S_{t}\text{WG}$ operator.

**Parameter**q	Ranking order	Best alternative
q=3	$\vartheta_{3}\;>\;\vartheta_{1}\;>\;\vartheta_{2}>\;\vartheta_{4}$	$\vartheta_{3}$
q=4	$\vartheta_{3}\;>\;\vartheta_{1}\;>\;\vartheta_{2}>\;\vartheta_{4}$	$\vartheta_{3}$
q=5	$\vartheta_{3}\;>\;\vartheta_{1}\;>\;\vartheta_{2}>\;\vartheta_{4}$	$\vartheta_{3}$
q=6	$\vartheta_{3}\;>\;\vartheta_{1}\;>\;\vartheta_{2}>\;\vartheta_{4}$	$\vartheta_{3}$
q=7	$\vartheta_{3}\;>\;\vartheta_{1}\;>\;\vartheta_{2}>\;\vartheta_{4}$	$\vartheta_{3}$
q=8	$\vartheta_{3}\;>\;\vartheta_{1}\;>\;\vartheta_{2}>\;\vartheta_{4}$	$\vartheta_{3}$
q=9	$\vartheta_{3}\;>\;\vartheta_{1}\;>\;\vartheta_{2}>\;\vartheta_{4}$	$\vartheta_{3}$
q=10	$\vartheta_{3}\;>\;\vartheta_{1}\;>\;\vartheta_{2}>\;\vartheta_{4}$	$\vartheta_{3}$

Which show that the attitudes of decision maker can be reflected by the various values of parameter. When the value of parameter lower decision-maker get more optimistic approach and if higher the value, more doubtful the decision-maker. So, decision-maker have the flexibility to select a suitable value of parameter according to their preferences.

### **5.4** Advantages

The proposed technique has different benefits:

1. Our proposed model effectively overcome the challenges posed by the existing approaches have also the ability to demonstrating high level of proficiency and provide robust framework to manage uncertainty across diverse scenarios.
2. Our proposed model having a unique characteristic to address the limitation of indeterminacy degree and provides the more flexible environment to the decision maker by relaxing the domain.
3. The suggested approach is proficient in addressing MCDM problems, particularly in the context of more realistic and complex scenarios.

## 6 Conclusion and future recommendation

The main goal of this article is to develop a flexible model, to cover the limitation of indeterminacy. Because in the existing approaches, we deal the uncertainties in MADM problem with MD and NMD associated with attributes, while it does not address the degree of indeterminacy and also it is important to admit that the existing theories has its specific limitations, which restricts decision-maker to evaluating alternatives within the domain. So, to tackle these limitations we proposed more flexible and free structure by merging NS with q-ROFSS and proposed more q-RONSS, characterized by three parameter, truth, indeterminacy and false membership with attributes provides the more flexible environment to the decision maker by relaxing the domain. This manuscript's primary achievement is to originate q-rung orthopair neutrosophic soft set and their basic operational laws. We also investigate some aggregation operators like q-RONSWA, q-RONSOWA, q-RONSWG and q-RONSOWG operators as well as a demonstration of their fundamental properties. Moreover, on the base of the proposed structure, we build a step-wise algorithm and mathematical model for solving MADM problem. At the end, for application, we consider a numerical example related to the personal selection problem of employ for a particular position in a real estate company and finally, show the superiority and authenticity of the proposed model using various analysis test analysis with the existing model.

A proposed structure has certain limitation, related to the personal selection problem of employees for a particular position in a real estate company on the bases of criteria. In this particular scenario, the scope of validation may be limited. It's important to assess the wide range of real-world decision challenge, to validate the robustness of $q-\text{RON}S_{f}S_{s}$ framework.

In future, the proposed model could find application in various direction such as TOPSIS Method, AHP Method, CODAS Method, quasirung orthopair fuzzy set [60], 3,4-quasirung fuzzy sets [61] and cubic interval-valued intuitionistic fuzzy subsemigroup and ideals [62], complex q-rung picture fuzzy Aczel–Alsina prioritized ordered operators [63], also in various fields, including business, engineering sectors, medical sector and different method for the selection of various projects.

## Funding

This research did not receive any external funding.

## Consent for publication

This manuscript has not been published and is not under consideration for publication elsewhere.

## Data availability statement

The accompanying manuscript does not contain any associated data. The paper only presents the written text and does not have any additional data that supports the claims and conclusions presented in the manuscript.

## CRediT authorship contribution statement

**Sumbal Ali:** Conceptualization. **Asad Ali:** Supervision. **Ahmad Bin Azim:** Software. **Ahmad Aloqaily:** Investigation. **Nabil Mlaiki:** Data curation.

## Declaration of competing interest

The authors declare that they have no known competing financial interests or personal relationships that could have appeared to influence the work reported in this paper.
