# Supplementary material for: Utilizing aggregation operators based on q-rung orthopair neutrosophic soft sets and their applications in multi-attributes decision making problems
Source: Heliyon. 2024 Jul 26;10(15):e35059. doi: 10.1016/j.heliyon.2024.e35059 (PMC11336292; doi:10.1016/j.heliyon.2024.e35059)
Supplement: Multimedia component 1 [file mmc1.pdf]

Plg report

by Ahmad Bin Azim

Submission date: 18-Jul-2024 02:03AM (UTC-0700)
Submission ID: 2258963187
File name: Ppr.pdf (1.35M)
Word count: 12146
Character count: 58759

Contents lists available at ScienceDirect

Heliyon

journal homepage: www.cell.com/heliyon

Original Research article

Utilizing Aggregation Operators Based On q-Rung Orthopair Neutrosophic Soft Sets and Their Applications in Multi-Attributes Decision Making Problems

Sumbal Ali[a,*], Asad Ali [a], Ahmad Bin Azim [a], Ahmad Aloqaily [b,c], Nabil Mlaiki [b]

[a] Department of Mathematics and Statistics, Hazara University, Mansehra 21300, Khyber Pakhtunkhwa, Pakistan

[b] Department of Mathematics and Sciences, Prince Sultan University, Riyadh 11587, Saudi Arabia

[c] School of Computer, Data and Mathematical Sciences, Western Sydney University, Australia, Sydney 2150

ARTICLE INFO

Keywords:
q-rung orthopair neutrosophic soft set,
Operational properties of q $-$ RONS$_f$S$_s$,
q-rung orthopair neutrosophic soft weighted averaging,
q-rung orthopair neutrosophic soft ordered weighted averaging,
q-rung orthopair neutrosophic soft weighted geometric,
q-rung orthopair neutrosophic soft ordered weighted geometric,
Decision-making and optimization

ABSTRACT

Neutrosophic sets provide greater versatility in dealing with a variety of uncertainties, including independent, partially independent, and entirely dependent scenarios, which q-ROF soft sets cannot handle. Indeterminacy, on the other hand, is ignored completely or partially by q-ROF soft sets. To address this issue, this study offers a unique novel concept as known as q-RONSS which combines neutrosophic set with q-ROF soft set. This technique addresses vagueness using a set of truth, indeterminacy, and false membership degrees associated with the parametrization tool, with the condition that the sum of the qth power of the truth, indeterminacy, and false membership degrees be less than or equal to one. In addition, this study outlines operational laws for the suggested structure. The main purpose this article is to define some averaging and geometric operators based on the q-rung orthopair neutrosophic soft set. Furthermore, this article provides a step-by-step method and a mathematical model for the suggested techniques. To solve a MADM issue, this research article proposes a numerical example of people selection for a specific position in a real estate business based on a variety of criteria. Finally, to demonstrate the proposed model's superiority and authenticity, this article performs several analyses, including sensitivity analysis, to address the reliability and influence of various parameter "q" values on the alternatives and the ultimate ranking outcomes using the averaging and geometric operators. A comparison of the proposed operators to current operators demonstrates the validity of the proposed structure. Furthermore, a comparison of the proposed structure to current theories demonstrates its superiority by overcoming their limits and offering a more flexible and adaptable framework. Finally, this study reviews the findings and consequences of our research.

* Corresponding author.
Department of Mathematics and Statistics,
Hazara University Mansehra 21300, Khyber Pakhtunkhwa, Pakistan.
E-mail address: asumbal302@gmail.com (S. Ali).

1. Introduction

In daily situations, decision-making (DM) is a complicated process that entails analysing several possibilities and selecting the optimal alternative based on decision makers' judgment. In the past, for the assessment of data decision makers only used real numbers, but with time experts assigned real numbers to evaluate the alternatives for decreasing the complexity of MADM problem.

Zadeh [1] established a strong mathematical framework called the fuzzy set in 1965. Fuzzy set have a wild application in various field see [28], [29],[30]. In 1975, Zadeh [2] provided intervals to each element with a broader range and developed interval-valued fuzzy set I-VFS with the restriction that LFS ≤ UFS. To solve MCGDM problem a novel technique of bipolar fuzzy set have a large rang of [-1,1], to navigate uncertainty in particular solutions was proposed by Zang [31,32]. A number of aggregation techniques have been developed with bipolar fuzzy sets in mind. Some notable examples are the efficient city supply chain management through spherical fuzzy dynamic multistage decision analysis proposed by Riaz et al. [38]; the hybrid multi-criteria decision-making method with a bipolar fuzzy approach and its applications to economic condition analysis introduced by Jana et al. [39]; and the MABAC framework for logarithmic bipolar fuzzy MAGDM for supplier selection proposed by Jana et al. [37]. Atanassov [20] developed the intuitionistic fuzzy set (IFS) in 1986 to address the gap in the non-MF in FS. Aggregation operators (AOs) were developed inside the IFS framework by Zhao et al. [3] and Tan et al. [4], [5]. When the value of non-membership is 0.7 and the value of membership is 0.8, intuitionistic fuzzy sets fail to meet their condition. In order to overcome this restriction, Yager [6] created the Pythagorean fuzzy set in 2013 and substituted the IFS constraint that the square of membership degree plus the square of non-membership degree must be between the [0,1]. Numerous aggregation operators have been proposed in the context of Pythagorean fuzzy sets. The following are some notable examples: portfolio selection in a Pythagorean fuzzy environment with a GRA and FAHP framework by Paul et al. [33]; managing climate change and controlling global warming with carbon-dioxide storage assessment in geological media under a Pythagorean fuzzy VIKOR and DEMATEL framework by Paul et al. [34]; and basic uncertain information order geometric aggregation operators proposed by Jin et al. [35]. In 2016, Yager [14] expanded upon the idea of IFS and PFS by substituting the condition of IFS and PFS with the condition that the q power of membership degree plus the q power of non-membership degree must belong to [0,1]. Within the framework of q-ROFS put forward by Liu and Wang [9], Liu and Liu [10], Liu et al. [11], Jana et al. [12], Garg and Chen [13], and Jana et al. [36], several aggregation operators have been presented. Furthermore, other aggregation operations were proposed by Seikh et al. [40], [41], and [42] in the context of q-ROFSs for MADM issues. A new cache reallocation-based page-level flash translation layer (CRFTL) was presented by Zhang et al. [45] along with its uses in smartphones; Chen et al. [46] suggested a non-negative temporal dimension preserved tensor completion model for the imputation of missing traffic data; and Lin et al. [47] suggested Linguistic q-ROFSs and their use in MADM issues. Lin et al. [48] use Pythagorean fuzzy sets to solve problems related to disease detection and cluster analysis, and they offer picture fuzzy interactional partitioned Heronian mean aggregation operators [49]. Ambiguity uncertainty in MADM problems are handled by MF and non-MF in the literature on classic fuzzy sets and its expansions. On the other hand, little information has been available regarding the level of uncertainty. It is significant to remember that current theories have particular restrictions within their fields. Smarandache [19] created a unique mathematical method in 1998 with tripled MF within the range of [0,3]. More efficiently, this tool deals with uncertainties by treating different kinds of scenarios: independent, somewhat independent, and totally dependent; on the other hand, fuzzy sets and all their generalized structures either wholly or partially neglect indeterminacy. Membership degrees (MD) and non-membership degrees (NMD) are normally evaluated using real values in all of the fuzzy structures that have been mentioned. But because MADM issues are so complex, Molodtsov [15] proposed soft sets in 1999 to overcome this constraint. These sets have now been merged with a variety of architectures (see [16], [17], [21], [22], and [23]). Soft sets use real numbers. Q-rung orthopair fuzzy soft sets are a novel notion that was first described by Hussain et al. [18]. The degree of indeterminacy in object appraisal is frequently overlooked, despite the focus on MD and NMD in current discourse. In order to get over this restriction, this study suggest a brand-new hybrid strategy known as q-RONSSs, which combines neutrosophic sets and q-ROFS$_f$S. This method is unique in that it includes parameters that quantify the degrees of falsehood, indeterminacy, and truth.

1.1. Literature Review

Neutrosophic sets (NS), in contrast to conventional fuzzy sets and its extensions, provide a more thorough knowledge of uncertainty. Including IDM improves the framework ability to handle uncertainty using indeterminate membership, which provide flexible and more effective approach to expressing complex information compared to traditional fuzzy sets. Neutrosophic set are well suited for different fields like AI, and data miming, effectively address the uncertainties in practical situations. Numerous experts have suggested various approaches for using Neutrosophic Sets (NSs). For instant, a serious of hybrid aggregation operators proposed by Garg and Nancy see [50]. A multi-distance interval-valued neutrosophic approach was proposed by Torkayesh et al. [51] to identify societal shortcomings in sustainable municipal garbage management. A multicriteria decision-making approach for 3D printing economic manufacturing utilizing a neutrosophic environment was presented by Liu and Tang [52]. In a neutrosophic set setting, Garg and Nancy [53] proposed a multi-criteria decision-making approach based on the prioritized Muirhead mean aggregation operator. Single-valued neutrosophic aggregation operations were proposed by Riaz et al. [54] for MCDM. Neutrosophic aggregation operators based on the soft max function were suggested by Garai et al. [55] and used in multi-attribute decision-making scenarios. Fuzzy set theory and its applications have advanced significantly in the last few years, examining a number of extensions and generalizations like q-SFRSs and q-ROPFSSs. In the context of q-ROPFSS, averaging aggregation operators was covered by Ali et al. [56]. Building on this, Ali et al. [57] addressed problems in green supply chain management by using the TOPSIS approach in a q-ROPF soft environment. q-spherical fuzzy rough sets were introduced by Azim et al. [58], who also showed how to use them in MADM scenarios. Furthermore, Azim et al. [59] investigated sine trigonometric q-SFR aggregation operators for group decision-making and digital transformation [60] and evaluated indoor positioning systems utilizing q-SFR TOPSIS analysis. Azim et al.'s [61] important addition was the application of the q-spherical fuzzy rough analytic hierarchy process for Industry 4.0 project prioritization. These sophisticated fuzzy set ideas offer strong frameworks to handle challenging situations involving decision-making in a variety of fields.

Upon analyzing the preceding discourse, it is evident that current investigations within the neutrosophic framework encounter challenges in effectively regulating the impact of an object's membership, indeterminacy membership, and non-membership within a set. In response to these limitations, we have introduced an innovative iteration of the q-ROFS, termed q–RONSSs. The primary objective of this novel construct is to precisely manage and govern the influence of MD, IMD, and NMD with attributes.

1.2. Research Gap

In the literature, traditional fuzzy sets and their extension sets handle the uncertainties with MD and NMD, but there was no information about the indeterminacy degree also it is important to admit that the existing theories have specific limitations, which restricts decision-makers to evaluating alternatives within the domain. So, to tackle these limitations we proposed a more flexible and free structure by merging neutrosophic sets to q-rung orthopair fuzzy soft sets and proposed q-rung orthopair neutrosophic soft sets, support by tripled MF associated with attributes which provide the more flexible environment to the decision maker by relaxing the domain.

1.3. Motivations

Across various fields many researchers are interested in using neutrosophic sets and their way of combining information. In the previous structure of q-ROFSS, there is a lack of indeterminacy parameters and also some restrictions on their domain to regulate the influence of membership degree in the DM process. So for this indeed more flexible structure of neutrosophic set to handle the various types of uncertainties including independent, partially independent, and completely dependent, while q-fuzzy set and all of its generalized structure are entirely or partially ignored indeterminacy, and proposed q-RON soft sets which provide more reliable and flexible than PFS, SFS, and NS, because of condition $0 \leq (\alpha) + (\beta) + (\gamma) \leq 1$, $0 \leq (\alpha)^2 + (\beta)^2 + (\gamma)^2 \leq 1$ and $0 \leq (\alpha) + (\beta) + (\gamma) \leq 3$ but also $0 \leq (\alpha)^q + (\beta)^q + (\gamma)^q \leq 3$.

1.4. Contributions

This research article presents several significant contributions:

i. To establish q-RONS sets.

ii. To define basic operational laws of q-rung orthopair neutrosophic soft sets.

iii. To develop aggregation operators based on the proposed structure such as $q - RONS_f WA$, $q - RONS_f OWA$, $q - RONS_f WG$ and $q - RONS_f OWG$ operators and to demon straight their fundamental properties.

iv. To address DM challenges, we adopted a novel technique of MCGDM within the framework of developed aggregation operators.

v. To address the reliability, of the proposed structure we performed a sensitivity analysis test.

vi. To demonstrate the comparative analysis, to show the authenticity of the proposed structure.

vii. To demonstrate characteristic analysis with existing theories, to express the superiority of the proposed structure.

This manuscript is organized as a Section 1 and serves as an introduction. Section 2, consists of basic preliminaries. Section 3, describes q-rung orthopair neutrosophic soft set and it's also developed averaging and geometric AOs based on the proposed structure and its related properties. Section 4, develops a mathematical model based on the proposed structure for solving the MCDM problem and considers a numerical example related to the personal selection problem of employment for a particular position in a real estate company for application. Section 5, the show shows the superiority and authenticity of the proposed model using a various analysis test with the existing model. In section 6, provide a conclusion. Figure 1 expresses the detailed layout of the article.

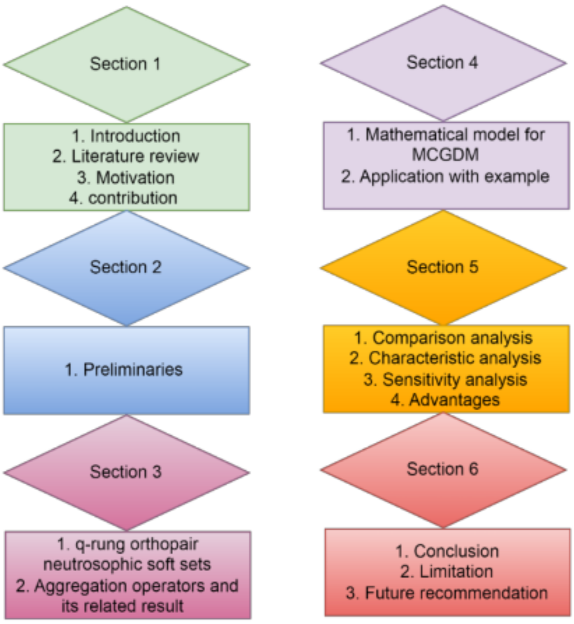

Figure 1 expresses the detailed layout of the article.

2. Preliminaries

This section presents a number of mathematical ideas. It begins with a thorough analysis of soft sets, IFS, PFS, q-ROFS, $q - ROFS_f S$ and NS.

Definition 1. [15] A pair $(F, ₳)$ is defined as a soft set over the parameters € and the fixed set $\mathbb{S}$, where $₳ \subseteq €$ and $F: ₳ \to P(\mathbb{S})$.

Definition 2. [20] Given a given set S, the mathematical structure of an IFS ₳ can be written as follows:

$$₳ = \{\langle \mu, \mathcal{L}_{₳}(\mathcal{S}), G_{₳}(\mathcal{S}) \rangle : \mathcal{S} \in \mathbb{S},\ \mathcal{L}_{₳}(\mathcal{S}), G_{₳}(\mathcal{S}) \in [0,1]\} \quad (1)$$

Where $\mathcal{L}_{Ⱥ}(\mathscr{s})$ and $G_{Ⱥ}(\mathscr{s})$ express mem and n-mem, such that $0 \leq (\mathcal{L}_{Ⱥ}(\mathscr{s})) + (G_{Ⱥ}(\mathscr{s})) \leq 1$ and the score value $S(Ⱥ) \in [-1,1]$ and accuracy function $A(Ⱥ) \in [0,1]$ is express as

$$S(Ⱥ) = \mathcal{L}_{Ⱥ}(\mathscr{s}) - G_{Ⱥ}(\mathscr{s})$$

$$A(Ⱥ) = \mathcal{L}_{Ⱥ}(\mathscr{s}) + G_{Ⱥ}(\mathscr{s}).$$

Equation (1) depicts the essential composition of IFS.

Definition 3. [6] P_yFS "Ⱥ" for a fixed set $\mathbb{S}$ is defined as

$$Ⱥ = \{\langle \mu, \mathcal{L}_{Ⱥ}(\mathscr{s}), G_{Ⱥ}(\mathscr{s})\rangle : \mathscr{s} \in \mathbb{S}, \mathcal{L}_{Ⱥ}(\mathscr{s}), G_{Ⱥ}(\mathscr{s}) \in [0,1]\} \quad (2)$$

Where $\mathcal{L}_{Ⱥ}(\mathscr{s})$ and $G_{Ⱥ}(\mathscr{s})$ express mem and n-mem, such that $0 \leq (\mathcal{L}_{Ⱥ}(\mathscr{s}))^2 + (G_{Ⱥ}(\mathscr{s}))^2 \leq 1$ and the score and accuracy value are express

$$S(Ⱥ) = (\mathcal{L}_{Ⱥ}(\mathscr{s}))^2 - (G_{Ⱥ}(\mathscr{s}))^2, S(Ⱥ) \in [-1,1]$$

$$A(Ⱥ) = (\mathcal{L}_{Ⱥ}(\mathscr{s}))^2 + (G_{Ⱥ}(\mathscr{s}))^2, A(Ⱥ) \in [0,1].$$

Equation (2) depicts the essential composition of PyFS.

Definition 4. Given a given set $\mathbb{S}$, the mathematical structure of a q-ROFS is represented by Ⱥ and is defined as follows:

$$Ⱥ=\{\langle \mu, \mathcal{L}_{Ⱥ}(\mathscr{s}), G_{Ⱥ}(\mathscr{s})\rangle : \mathscr{s} \in \mathbb{S}, \mathcal{L}_{Ⱥ}(\mathscr{s}), G_{Ⱥ}(\mathscr{s}) \in [0,1]\} \quad (3)$$

Where the membership and non-membership degrees are represented by $\mathcal{L}_{Ⱥ}(\mathscr{s})$ and $G_{Ⱥ}(\mathscr{s})$, such that $0 \leq (\mathcal{L}_{Ⱥ}(\mathscr{s}))^q + (G_{Ⱥ}(\mathscr{s}))^q \leq 1$.

The accuracy as well as and score values are written as:

$$S(Ⱥ) \in [-1,1] = (\mathcal{L}_{Ⱥ}(\mathscr{s}))^q - (G_{Ⱥ}(\mathscr{s}))^q$$

$$A(Ⱥ) \in [0,1] = (\mathcal{L}_{Ⱥ}(\mathscr{s}))^q + (G_{Ⱥ}(\mathscr{s}))^q.$$

Equation (3) represent the mathematical structure of an q-rung orthopair fuzzy set. Figure 2 represent the difference between intuitionistic fuzzy set, Pythagorean fuzzy set and q-rung orthopair fuzzy set.

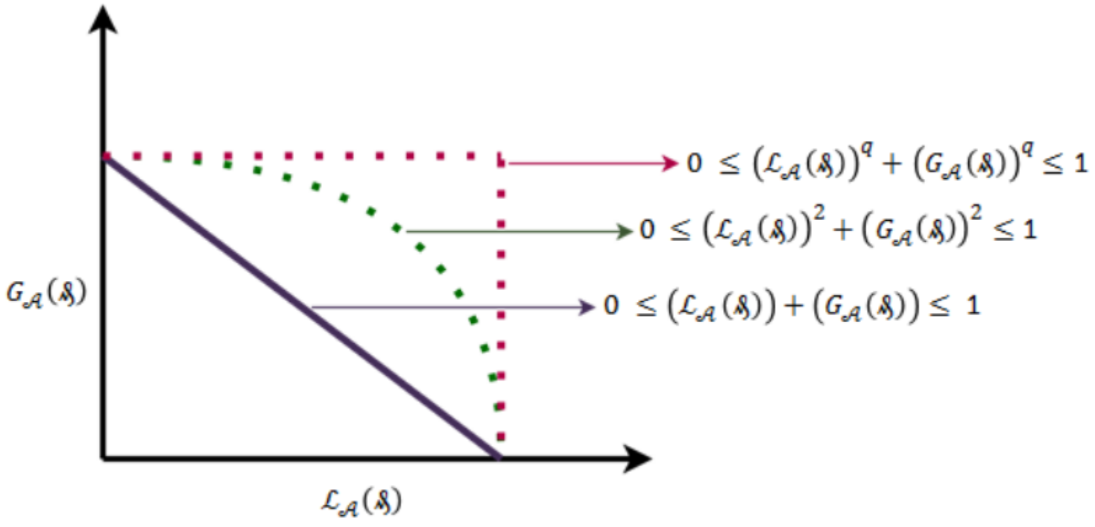

Figure 2. Express the analysis of distinctions among IF, PyF and q-ROF spaces.

Definition 5. [18] A pair $(\mathbb{A}, \mathbb{C})$ is known as q-ROFS$_f$S over the soft universe $(\mathbb{S}, \mathbb{C})$ and $\mathbb{C} \subseteq \mathbb{E}$. Where $\mathbb{A}$ is given by $\mathbb{A}: \mathbb{C} \to q - ROFS^{(\mathbb{S})}$ and defined as ƅ i j

$$\mathbb{A}_{b_j}(\mu_i) = \{\langle \mathcal{S}_i, \mathcal{L}_j(\mathcal{S}_i), G_j(\mathcal{S}_i) \rangle : \mathcal{S}_i \in \mathbb{S}\} \quad (4)$$

Where $\mathcal{L}_j(\mathcal{S}_i)$ and $G_j(\mathcal{S}_i)$ express mem and n-mem $\mathcal{S}_i \in \mathbb{S}$ to a set $\mathbb{A}_{b_j}(\mathcal{S}_i)$, such that $0 \le \left(\mathcal{L}_j(\mathcal{S}_i)\right)^q + \left(G_j(\mathcal{S}_i)\right)^q \le 1 \; (q \ge 1)$

and the indeterminacy degree is expressed as $\pi_{\mathbb{A}_{b_j}} = \sqrt[q]{1 - \left(\left(\mathcal{L}_j(\mathcal{S}_i)\right)^q + \left(G_j(\mathcal{S}_i)\right)^q\right)}$.

Equation (4) represent the mathematical structure of an q-rung orthopair fuzzy soft set.

Definition 6. [19] Let $\mathbb{S}$ be a fixed set. A neutrosophic set $\mathbb{A}$ is defined as:

$$\mathbb{A} = \{\langle \mu, \mathcal{L}_{\mathbb{A}}(\mathcal{S}), G_{\mathbb{A}}(\mathcal{S}), Ł_{\mathbb{A}}(\mathcal{S}) \rangle : \mu \in \mathbb{S}, \mathcal{L}_{\mathbb{A}}(\mathcal{S}), G_{\mathbb{A}}(\mathcal{S}), Ł_{\mathbb{A}}(\mathcal{S}) \in [0,1]\} \quad (5)$$

Where $\mathcal{L}_{\mathbb{A}}(\mathcal{S})$, $G_{\mathbb{A}}(\mathcal{S})$ and $Ł_{N}(\mathcal{S})$ express truth, indeterminacy, and falsity degree, such that $0 \le (\mathcal{L}_{\mathbb{A}}(\mathcal{S})) + \left(G_{\mathbb{A}}(\mathcal{S}) + (Ł_{\mathbb{A}}(\mathcal{S}))\right) \le 3$.

The score and accuracy values are written as:

$$S(\mathbb{A}) = (\mathcal{L}_{\mathbb{A}}(\mathcal{S})) - (G_{\mathbb{A}}(\mathcal{S})) - (Ł_{\mathbb{A}}(\mathcal{S}))$$

$$A(\mathbb{A}) = (\mathcal{L}_{\mathbb{A}}(\mathcal{S})) + (G_{\mathbb{A}}(\mathcal{S})) + (Ł_{\mathbb{A}}(\mathcal{S})).$$

Equation (5) depicts the essential composition of NS.

Definition 7. Using the t-norm $\mathcal{T}$, t-conorm $\mathcal{S}$, Einstein's operations are as follows:

$$\mathcal{T}_E(X, Y) = \frac{X+Y}{1+(1-X)(1-Y)} \quad (6)$$

$$\mathcal{S}_E(X, Y) = \frac{X+Y}{1+XY} \quad (7)$$

Equation (6) and Equation (7) represent the t-norm and t-conorm of Einstein operations.

3. q-rung orthopair neutrosophic soft set:

This section is devoted to a thorough investigation of the operational laws guiding the proposed structure of q-RONSS. It also includes an explanation of the basic features of aggregation operators, including $q - RONS_fWA$, $q - RONS_fOWA$, $q - RONS_fWG$ and $q - RONS_fOWG$ operators, as well as their construction and analysis.

Definition 8. Let $(\mathbb{S}, \mathbb{C})$ be a soft universe and $\mathbb{C} \subseteq \mathbb{E}$. And the pair $(\mathbb{A}, \mathbb{C})$ over $\mathbb{S}$, where $\mathbb{A}$ is a function given by $\mathbb{A}: \mathbb{C} \to q - RONFS^{(\mathbb{S})}$ is express q-RONS$_f$S which is defined as

$$\mathbb{A}_{b_j}(\mathcal{S}_i) = \{\langle \mathcal{S}_i, \mathcal{T}_j(\mathcal{S}_i), ł_j(\mathcal{S}_i), \mathcal{F}_j(\mathcal{S}_i) \rangle : \mathcal{S}_i \in \mathbb{S} \text{ and } q \ge 1\} \quad (8)$$

Where $\mathcal{T}_j(\mathcal{S}_i), ł_j(\mathcal{S}_i), \mathcal{F}_j(\mathcal{S}_i) \in [0,1]$ signify the degree of truth, indeterminacy, and falsity of memberships of $\mathcal{S}_i \in \mathbb{S}$ to a set $\mathbb{A}_{b_j}(\mathcal{S}_i)$, and respectively

$$0 \le \left(\mathcal{T}_j(\mathcal{S}_i)\right)^q + \left(ł_j(\mathcal{S}_i)\right)^q + \left(\mathcal{F}_j(\mathcal{S}_i)\right)^q \le 3 \; (q \ge 1)$$

For convenience $\mathbb{A}_{b_j}(\mathcal{S}_i) = \langle \mathcal{S}_i, \mathcal{T}_j(\mathcal{S}_i), ł_j(\mathcal{S}_i), \mathcal{F}_j(\mathcal{S}_i) \rangle_q$ called q-RONS$_f$S. And the degree of hesitancy is defined as

$$\pi_{Ⱥ_{ƀ_{ij}}} = \sqrt[q]{1-\left(\left(Ʈ_j(\mathcal{S}_i)\right)^q + \left(ƚ_j(\mathcal{S}_i)\right)^q + \left(Ƒ_j(\mathcal{S}_i)\right)^q\right)}.$$

Equation (8) represent the mathematical structure of an q-rung orthopair neutrosophic soft set.

Definition 9. Consider be any three q-RONF soft numbers $Ⱥ = (Ʈ, ƚ, Ƒ)$ and $Ⱥ_{ƀ_{1j}} = (Ʈ_{1j}, ƚ_{1j}, Ƒ_{1j})$ for j = 1,2 and $\lambda > 0$., the operations are defined as follows

(i). $Ⱥ^C = (Ƒ, ƚ, Ʈ)$

(ii). $Ⱥ_{ƀ_{11}} \leq Ⱥ_{ƀ_{12}}$ if and only if $(Ʈ_{11} \leq Ʈ_{12}, ƚ_{11} \geq ƚ_{12}, Ƒ_{11} \geq Ƒ_{12})$

(iii). $Ⱥ_{ƀ_{11}} \cup Ⱥ_{ƀ_{12}} = (\max(Ʈ_{11}, Ʈ_{12}), \min(ƚ_{11}, ƚ_{12}), \min(Ƒ_{11}, Ƒ_{12}))$

(iv). $Ⱥ_{ƀ_{11}} \cap Ⱥ_{ƀ_{12}} = (\min(Ʈ_{11}, Ʈ_{12}), \max(ƚ_{11}, ƚ_{12}), \max(Ƒ_{11}, Ƒ_{12}))$

(v). $Ⱥ_{ƀ_{11}} \oplus Ⱥ_{ƀ_{12}} = \left(\sqrt[q]{(Ʈ_{11})^q + (Ʈ_{12})^q - (Ʈ_{11})^q(Ʈ_{12})^q}, ƚ_{11}ƚ_{12}, Ƒ_{11}Ƒ_{12}\right)$

(vi). $Ⱥ_{ƀ_{11}} \otimes Ⱥ_{ƀ_{12}} = \left(Ʈ_{11}Ʈ_{12}, \sqrt[q]{(ƚ_{11})^q + (ƚ_{12})^q - (ƚ_{11})^q(ƚ_{12})^q}, \sqrt[q]{(Ƒ_{11})^q + (Ƒ_{12})^q - (Ƒ_{11})^q(Ƒ_{12})^q}\right)$

(vii). $\lambda Ⱥ = \left(\sqrt[q]{1-(1-(Ʈ)^q)^\lambda}, (ƚ)^\lambda, (Ƒ)^\lambda\right)$

(viii). $Ⱥ^\lambda = \begin{pmatrix} (Ʈ)^\lambda, \\ \sqrt[q]{1-(1-(ƚ)^q)^\lambda}, \\ \sqrt[q]{1-(1-(Ƒ)^q)^\lambda} \end{pmatrix}.$

Definition 10. A score function of q-RONFS$_f$N$_s$ $Ⱥ_{ƀ_{ij}} = (Ʈ_{ij}, ƚ_{ij}, Ƒ_{ij})$ can be defined as

$$S\left(Ⱥ_{ƀ_{ij}}\right) = S\left(Ⱥ_{ƀ_{ij}}\right) \in [-3,3] = (Ʈ_{ij})^q - (ƚ_{ij})^q - (Ƒ_{ij})^q + \left(\frac{e^{(Ʈ_{ij})^q - (ƚ_{ij})^q - (Ƒ_{ij})^q}}{e^{(Ʈ_{ij})^q - (ƚ_{ij})^q - (Ƒ_{ij})^q} + 1} - \frac{1}{2}\right)\pi^q_{Ⱥ_{ƀ_{ij}}}, (q \geq 1) \quad (9)$$

Equation (9) represent the score function of q-rung orthopair neutrosophic soft set.

Definition 11. The accuracy function for q-RONFS$_t$N$_s$ $Ⱥ_{ƀ_{ij}} = (Ʈ_{ij}, ƚ_{ij}, Ƒ_{ij})$ is defined as

$$Acc\left(Ⱥ_{ƀ_{ij}}\right) = (Ʈ_{ij})^q + (ƚ_{ij})^q + (Ƒ_{ij})^q$$

Definition 12. Let $Ⱥ_{ƀ_{11}} = (Ʈ_{11}, ƚ_{11}, Ƒ_{11})$ and $Ⱥ_{ƀ_{12}} = (Ʈ_{12}, ƚ_{12}, Ƒ_{12})$ be two $q-$RONFS$_f$N$_s$. Then

I. $S(Ⱥ_{ƀ_{11}}) < S(Ⱥ_{ƀ_{12}})$, $Ⱥ_{ƀ_{11}} < Ⱥ_{ƀ_{12}}$

II. $S(Ⱥ_{ƀ_{11}}) > S(Ⱥ_{ƀ_{12}})$, $Ⱥ_{ƀ_{11}} > Ⱥ_{ƀ_{12}}$

III. $S(Ⱥ_{ƀ_{11}}) = S(Ⱥ_{ƀ_{12}})$, then

   a. $Acc(Ⱥ_{ƀ_{11}}) < Acc(Ⱥ_{ƀ_{12}})$ then $Ⱥ_{ƀ_{11}} < Ⱥ_{ƀ_{12}}$
   b. $Acc(Ⱥ_{ƀ_{11}}) > Acc(Ⱥ_{ƀ_{12}})$ then $Ⱥ_{ƀ_{11}} > Ⱥ_{ƀ_{12}}$
   c. $Acc(Ⱥ_{ƀ_{11}}) = Acc(Ⱥ_{ƀ_{12}})$ then $Ⱥ_{ƀ_{11}} = Ⱥ_{ƀ_{12}}$

Example 1. Assume a decision-maker who is in the process of selecting a smartwatch from a market offering four alternatives $Ύ = \{O_1, O_2, O_3, O_4\}$ associated with specific parameters $€ = \{x_1, x_2, x_3, x_4\}$

x_1 = Fitness Features

x_2 = Assess Notifications

x_3 = Smart Compass

x_4 = Play Music

Table 1. Represent q − $RONS_fN_s$ $Ⱥ_{ƀ_{ij}}$=($Ƭ_{ij}$,$ɨ_{ij}$,$Ƒ_{ij}$) for q ≥ 3.

Ƴ	Fitness Features	Assess Notifications	Smart Compass	Play Music
O_1	(.8,0.5,0.6)	(0.6,0.2,0.4)	(0.4,0.1,0.4)	(0.6,0.4,0.2)
O_2	(.6,0.3,0.2)	(0.4,0.1,0.3)	(0.7,0.3,0.4)	(0.5,0.3,0.4)
O_3	(.5,0.2,0.1)	(0.7,0.2,0.5)	(0.3,0.1,0.6)	(0.5,0.5,0.1)
O_4	(.4,0.3,0.2)	(0.8,0.1,0.1)	(0.3,0.3,0.4)	(0.7,0.1,0.2)

When we assess the various options using the assigned rating values, we obtain the outcome present in "Table 1", which is expressed in the form of q − $RONFS_fN_s$.

Theorem 1: Let $Ⱥ_{ƀ_{ij}} = (Ƭ_{ij}, ɨ_{ij}, Ƒ_{ij})$ and Ⱥ = (Ƭ, ɨ, Ƒ) be any two q-$RONFS_fN_s$and λ, λ_1, λ_2 >0, having the properties.

i. $Ⱥ_{ƀ_{11}} \oplus Ⱥ_{ƀ_{12}} = Ⱥ_{ƀ_{12}} \oplus Ⱥ_{ƀ_{11}}$

ii. $Ⱥ_{ƀ_{11}} \otimes Ⱥ_{ƀ_{12}} = Ⱥ_{ƀ_{12}} \otimes Ⱥ_{ƀ_{11}}$

iii. $\lambda(Ⱥ_{ƀ_{11}} \oplus Ⱥ_{ƀ_{12}}) = \lambda Ⱥ_{ƀ_{11}} \oplus \lambda Ⱥ_{ƀ_{12}}$

iv. $(\lambda_1 \oplus \lambda_2)Ⱥ = \lambda_1 Ⱥ \oplus \lambda_2 Ⱥ$

v. $Ⱥ^{(\lambda_1 \oplus \lambda_2)} = Ⱥ^{\lambda_1} \otimes Ⱥ^{\lambda_2}$

vi. $Ⱥ^{\lambda}_{ƀ_{11}} \otimes Ⱥ^{\lambda}_{ƀ_{12}} = (Ⱥ_{ƀ_{11}} \oplus Ⱥ_{ƀ_{12}})^{\lambda}$.

Proof: Straightforward.

3.2. Weighted Averaging Operator Using q-RONS set:

Let $Ⱥ_{ƀ_{ij}}$=($Ƭ_{ij}$,$ɨ_{ij}$,$Ƒ_{ij}$) $(i = 1,2,3, \dots, n)(j = 1,2,3, \dots, m)$ be a family of q-$RONFS_fN_s$ having the weight vector of the experts x_i is ω_i and for the parameters e_j is υ_j such that $\omega_i, \upsilon_j \in [0,1]$ with the constraints that $\sum_{i=1}^{n} \omega_i = 1$ and $\sum_{j=1}^{m} \upsilon_j = 1$ respectively.

Then q-$RONS_fWA$ operator is defined by the mapping q-$RONS_fWA$: $Đ^n \longrightarrow Đ$, where Đ represents the collection of q-$RONFS_fN_s$.

$$\text{q-RONS}_f\text{WA}(Ⱥ_{ƀ_{11}}, Ⱥ_{ƀ_{12}}, \dots, Ⱥ_{ƀ_{nm}}) = \oplus_{j=1}^{m} \upsilon_i \left(\oplus_{i=1}^{n} \omega_i Ⱥ_{ƀ_{ij}}\right)$$

Theorem 2: Let $Ⱥ_{ƀ_{ij}}$=($Ƭ_{ij}$,$ɨ_{ij}$,$Ƒ_{ij}$) be a collection of q-$RONFS_fN_s$. Then, the aggregation result for the q- q-$RONS_fWA$ operator is defined as:

$$\text{q-RONS}_f\text{WA}(Ⱥ_{ƀ_{11}}, Ⱥ_{ƀ_{12}}, \dots, Ⱥ_{ƀ_{nm}}) = \oplus_{j=1}^{m} \upsilon_i \left(\oplus_{i=1}^{n} \omega_i Ⱥ_{ƀ_{ij}}\right)$$

$$= \left(\sqrt[q]{1 - \prod_{j=1}^{m}\left(\prod_{i=1}^{n}\left(1 - Ƭ_{ij}^{q}\right)^{\omega_i}\right)^{\upsilon_j}}, \prod_{j=1}^{m}\left(\prod_{i=1}^{n} ɨ_{ij}^{\omega_i}\right)^{\upsilon_j}, \prod_{j=1}^{m}\left(\prod_{i=1}^{n} Ƒ_{ij}^{\omega_i}\right)^{\upsilon_j}\right)$$

$$\text{q-RONS}_f\text{WA}(Ⱥ_{ƀ_{11}}, Ⱥ_{ƀ_{12}}, \dots, Ⱥ_{ƀ_{nm}}) = \begin{pmatrix} \sqrt[q]{1 - \prod_{j=1}^{m}\left(\prod_{i=1}^{n}\left(1 - Ƭ_{ij}^{q}\right)^{\omega_i}\right)^{\upsilon_j}}, \\ \prod_{j=1}^{m}\left(\prod_{i=1}^{n} ɨ_{ij}^{\omega_i}\right)^{\upsilon_j}, \\ \prod_{j=1}^{m}\left(\prod_{i=1}^{n} Ƒ_{ij}^{\omega_i}\right)^{\upsilon_j} \end{pmatrix} \quad (10)$$

The q-rung orthopair neutrosophic soft weighted averaging operator is represented by equation (10).

Where ω_i and υ_j are the WV for expert x_i and parameters e_j, respectively, with the condition $\sum_{i=1}^{n} \omega_i = 1$ and $\sum_{j=1}^{m} \upsilon_j = 1$..

Proof: By mathematical induction, we solve this result. We have,

$\text{Ⱥ}_{\mathfrak{b}_{11}} \oplus \text{Ⱥ}_{\mathfrak{b}_{12}} = \left(\sqrt[q]{(\text{Ʈ}_{11})^q + (\text{Ʈ}_{12})^q - (\text{Ʈ}_{11})^q(\text{Ʈ}_{12})^q},\ \text{ɨ}_{11}\text{ɨ}_{12},\ \text{Ƒ}_{11}\text{Ƒ}_{12}\right)$ and $\lambda\text{Ⱥ} = \left(\left(\sqrt[q]{1-[1-\text{Ʈ}^q]^{\lambda}}, \text{ɨ}^{\lambda}, \text{Ƒ}^{\lambda}\right)\right)$.

Step 1: Initially, examine the case when n is equal to 2 and m is equal to 2.

$$\text{q-RONS}_f\text{WA}\left(\text{Ⱥ}_{\mathfrak{b}_{11}}, \text{Ⱥ}_{\mathfrak{b}_{12}}, \dots, \text{Ⱥ}_{\mathfrak{b}_{nm}}\right) = \oplus_{j=1}^{2} \upsilon_i \left(\oplus_{i=1}^{2} \omega_i \text{Ⱥ}_{\mathfrak{b}_{ij}}\right)$$

$$= \upsilon_1\left(\oplus_{i=1}^{2} \omega_i \text{Ⱥ}_{\mathfrak{b}_{11}}\right) \oplus \upsilon_2\left(\oplus_{i=1}^{2} \omega_i \text{Ⱥ}_{\mathfrak{b}_{12}}\right)$$

$$= \upsilon_1\left(\omega_1 \text{Ⱥ}_{\mathfrak{b}_{11}} \oplus \omega_2 \text{Ⱥ}_{\mathfrak{b}_{21}}\right) \oplus \upsilon_2\left(\omega_1 \text{Ⱥ}_{\mathfrak{b}_{12}} \oplus \omega_2 \text{Ⱥ}_{\mathfrak{b}_{22}}\right)$$

$$\therefore \omega_1 \text{Ⱥ}_{\mathfrak{b}_{11}} = \left(\sqrt[q]{1-\left(1-\text{Ʈ}_{11}^q\right)^{\omega_1}}, \text{ɨ}_{11}^{\omega_1}, \text{Ƒ}_{11}^{\omega_1}\right) \text{ and } \omega_2 \text{Ⱥ}_{\mathfrak{b}_{21}} = \left(\sqrt[q]{1-\left(1-\text{Ʈ}_{21}^q\right)^{\omega_2}}, \text{ɨ}_{21}^{\omega_2}, \text{Ƒ}_{21}^{\omega_2}\right)$$

$$= \upsilon_1\left\{\left(\sqrt[q]{1-\left(1-\text{Ʈ}_{11}^q\right)^{\omega_1}}, \text{ɨ}_{11}^{\omega_1}, \text{Ƒ}_{11}^{\omega_1}\right) \oplus \left(\sqrt[q]{1-\left(1-\text{Ʈ}_{21}^q\right)^{\omega_2}}, \text{ɨ}_{21}^{\omega_2}, \text{Ƒ}_{21}^{\omega_2}\right)\right\} \oplus \upsilon_2\left\{\left(\sqrt[q]{1-\left(1-\text{Ʈ}_{12}^q\right)^{\omega_1}}, \text{ɨ}_{12}^{\omega_1}, \text{Ƒ}_{12}^{\omega_1}\right) \oplus \left(\sqrt[q]{1-\left(1-\text{Ʈ}_{22}^q\right)^{\omega_2}}, \text{ɨ}_{22}^{\omega_2}, \text{Ƒ}_{22}^{\omega_2}\right)\right\}$$

$$= \upsilon_1\left(\sqrt[q]{1-\Pi_{i=1}^{2}\left(1-\text{Ʈ}_{i1}^q\right)^{\omega_i}}, \Pi_{i=1}^{2}\text{ɨ}_{i1}^{\omega_i}, \Pi_{i=1}^{2}\text{Ƒ}_{i1}^{\omega_i}\right) \oplus \upsilon_2\left(\sqrt[q]{1-\Pi_{i=1}^{2}\left(1-\text{Ʈ}_{i2}^q\right)^{\omega_i}}, \Pi_{i=1}^{2}\text{ɨ}_{i2}^{\omega_i}, \Pi_{i=1}^{2}\text{Ƒ}_{i2}^{\omega_i}\right)$$

$$= \upsilon_1\left(\sqrt[q]{1-\left(\Pi_{i=1}^{2}\left(1-\text{Ʈ}_{i1}^q\right)^{\omega_i}\right)^{\upsilon_1}}, \left(\Pi_{i=1}^{2}\text{ɨ}_{i1}^{\omega_i}\right)^{\upsilon_1}, \left(\Pi_{i=1}^{2}\text{Ƒ}_{i1}^{\omega_i}\right)^{\upsilon_1}\right) \oplus \upsilon_2\left(\sqrt[q]{1-\left(\Pi_{i=1}^{2}\left(1-\text{Ʈ}_{i2}^q\right)^{\omega_i}\right)^{\upsilon_2}}, \left(\Pi_{i=1}^{2}\text{ɨ}_{i2}^{\omega_i}\right)^{\upsilon_2}, \left(\Pi_{i=1}^{2}\text{Ƒ}_{i2}^{\omega_i}\right)^{\upsilon_2}\right)$$

$$= \left(\sqrt[q]{1-\Pi_{j=1}^{2}\left(\Pi_{i=1}^{2}\left(1-\text{Ʈ}_{ij}^q\right)^{\omega_i}\right)^{\upsilon_j}}, \Pi_{j=1}^{2}\left(\Pi_{i=1}^{2}\text{ɨ}_{ij}^{\omega_i}\right)^{\upsilon_j}, \Pi_{j=1}^{2}\left(\Pi_{i=1}^{2}\text{Ƒ}_{ij}^{\omega_i}\right)^{\upsilon_j}\right)$$

Step 2: In second step, we consider for n=κ_1 and m=κ_2.

$$\text{q-RONS}_f\text{WA}\left(\text{Ⱥ}_{\mathfrak{b}_{11}}, \text{Ⱥ}_{\mathfrak{b}_{12}}, \dots, \text{Ⱥ}_{\mathfrak{b}_{\kappa_1\kappa_2}}\right) = \oplus_{j=1}^{\kappa_2} \upsilon_i \left(\oplus_{i=1}^{\kappa_1} \omega_i \text{Ⱥ}_{\mathfrak{b}_{ij}}\right) = \left(\sqrt[q]{1-\Pi_{j=1}^{\kappa_2}\left(\Pi_{i=1}^{\kappa_1}\left(1-\text{Ʈ}_{ij}^q\right)^{\omega_i}\right)^{\upsilon_j}}, \Pi_{j=1}^{\kappa_2}\left(\Pi_{i=1}^{\kappa_1}\text{ɨ}_{ij}^{\omega_i}\right)^{\upsilon_j}, \Pi_{j=1}^{\kappa_2}\left(\Pi_{i=1}^{\kappa_1}\text{Ƒ}_{ij}^{\omega_i}\right)^{\upsilon_j}\right)$$

Step 3: Finally, for n=κ_1+1 and m=κ_2+1.

$$\text{q-RONS}_f\text{WA}\left(\text{Ⱥ}_{\mathfrak{b}_{11}}, \text{Ⱥ}_{\mathfrak{b}_{12}}, \dots, \text{Ⱥ}_{\mathfrak{b}_{\kappa_1+1\,\kappa_2+1}}\right) = \left\{\oplus_{j=1}^{\kappa_2} \upsilon_j \left(\oplus_{i=1}^{\kappa_1} \omega_i \text{Ⱥ}_{\mathfrak{b}_{ij}}\right)\right\} \oplus \upsilon_{(\kappa_1+1)} \left(\omega_{\kappa_2+1} \text{Ⱥ}_{\mathfrak{e}_{(\kappa_1+1)(\kappa_2+1)}}\right)$$

$$= \begin{pmatrix} \sqrt[q]{1-\prod_{j=1}^{\kappa_2}\left(\prod_{i=1}^{\kappa_1}\left(1-\text{Ʈ}_{ij}^q\right)^{\omega_i}\right)^{\upsilon_j}}, \\ \Pi_{j=1}^{\kappa_2}\left(\Pi_{i=1}^{\kappa_1}\text{ɨ}_{ij}^{\omega_i}\right)^{\upsilon_i}, \Pi_{j=1}^{\kappa_2}\left(\Pi_{i=1}^{\kappa_1}\text{Ƒ}_{ij}^{\omega_i}\right)^{\upsilon_j} \end{pmatrix} \oplus \upsilon_{(\kappa_1+1)} \left(\omega_{\kappa_2+1} \text{Ⱥ}_{\mathfrak{b}_{(\kappa_1+1)(\kappa_2+1)}}\right)$$

$$= \left(\sqrt[q]{1-\Pi_{j=1}^{(\kappa_2+1)}\left(\Pi_{i=1}^{(\kappa_1+1)}\left(1-\text{Ʈ}_{ij}^q\right)^{\omega_i}\right)^{\upsilon_j}}, \Pi_{j=1}^{(\kappa_2+1)}\left(\Pi_{i=1}^{(\kappa_1+1)}\text{ɨ}_{ij}^{\omega_i}\right)^{\upsilon_j}, \Pi_{j=1}^{(\kappa_2+1)}\left(\Pi_{i=1}^{(\kappa_1+1)}\text{Ƒ}_{ij}^{\omega_i}\right)^{\upsilon_j}\right)$$

Now, in the cases where n=κ_1+1 and m=κ_2+1, where $\forall$ m, n $\geq$ 1, Equation (10) holds true. Given that $\text{Ⱥ}_{\mathfrak{b}_{ij}} = (\text{Ʈ}_{ij}, \text{ɨ}_{ij}, \text{Ƒ}_{ij})$ denote a collection of q-RONS$_f$N$_s$, where $0 \le \text{Ʈ}_{ij}, \text{ɨ}_{ij}, \text{Ƒ}_{ij} \le 3$ satisfies the condition $0 \le \text{Ʈ}_{ij} + \text{ɨ}_{ij} + \text{Ƒ}_{ij} \le 3$ for expert x_i and parameters e_j, with weights ω_i such that $\sum_{i=1}^{n} \omega_i = 1$ and υ_j such that $\sum_{j=1}^{m} \upsilon_j = 1$.

$0 \le \text{Ʈ}_{ij} \le 3$

$\Rightarrow 0 \le 1 - \text{Ʈ}_{ij} \le 3$

$$\Rightarrow 0 \le \left(1-\mathcal{T}_{ij}^{q}\right)^{\omega_i} \le 3$$

$$\Rightarrow 0 \le \Pi_{i=1}^{n}\left(1-\mathcal{T}_{ij}^{q}\right)^{\omega_i} \le 3$$

$$\Rightarrow 0 \le \Pi_{j=1}^{m}\left(\Pi_{i=1}^{n}\left(1-\mathcal{T}_{ij}^{q}\right)^{\omega_i}\right)^{\upsilon_j} \le 3$$

$$\Rightarrow 0 \le \sqrt[q]{\Pi_{j=1}^{m}\left(\Pi_{i=1}^{n}\left(1-\mathcal{T}_{ij}^{q}\right)^{\omega_i}\right)^{j}} \le 3.$$

Now, for $0 \le \mathcal{I}_{ij} \le 3$

$$\Rightarrow 0 \le \Pi_{i=1}^{n}\, \mathcal{I}_{ij}^{\omega_i} \le 3$$

$$\Rightarrow 0 \le \Pi_{j=1}^{m}\left(\Pi_{i=1}^{n}\, \mathcal{I}_{ij}^{\omega_i}\right)^{\upsilon_j} \le 3$$

and finally

$$0 \le \mathcal{F}_{ij} \le 3$$

$$\Rightarrow 0 \le \Pi_{i=1}^{n}\, \mathcal{F}_{ij}^{\omega_i} \le 3$$

$$\Rightarrow 0 \le \Pi_{j=1}^{m}\left(\Pi_{i=1}^{n}\, \mathcal{F}_{ij}^{\omega_i}\right)^{\upsilon_j} \le 3.$$

As, $0 \le \mathcal{T}_{ij}^{q} + \mathcal{I}_{ij}^{q} + \mathcal{F}_{ij}^{q} \le 3 \Rightarrow \mathcal{T}_{ij}^{q} + \mathcal{F}_{ij}^{q} \le 1 - \mathcal{I}_{ij}^{q}$

$$\Rightarrow \Pi_{i=1}^{n}\left(\mathcal{T}_{ij}^{q}\right)^{\omega_i} + \Pi_{i=1}^{n}\left(\mathcal{F}_{ij}^{q}\right)^{\omega_i} \le \Pi_{i=1}^{n}\left(1-\mathcal{I}_{ij}^{q}\right)^{\omega_i}$$

$$\Rightarrow \Pi_{j=1}^{m}\left(\Pi_{i=1}^{n}\left(\mathcal{T}_{ij}^{q}\right)^{\omega_i}\right)^{\upsilon_j} + \Pi_{j=1}^{m}\left(\Pi_{i=1}^{n}\left(\mathcal{F}_{ij}^{q}\right)^{\omega_i}\right)^{\upsilon_j} \le \Pi_{j=1}^{m}\left(\Pi_{i=1}^{n}\left(1-\mathcal{I}_{ij}^{q}\right)^{\omega_i}\right)^{\upsilon_j}$$

Now, we have

$$0 \le \left\{\sqrt[q]{1-\Pi_{j=1}^{m}\left(\Pi_{i=1}^{n}\left(1-\mathcal{I}_{ij}^{q}\right)^{\omega_i}\right)^{\upsilon_j}}\right\}^{q} + \left\{\Pi_{j=1}^{m}\left(\Pi_{i=1}^{n}\mathcal{T}_{ij}^{\omega_i}\right)^{\upsilon_j}\right\}^{q} + \left\{\Pi_{j=1}^{m}\left(\Pi_{i=1}^{n}\mathcal{F}_{ij}^{\omega_i}\right)^{\upsilon_j}\right\}^{q},$$

$$0 \le 1-\Pi_{j=1}^{m}\left(\Pi_{i=1}^{n}\left(1-\mathcal{I}_{ij}^{q}\right)^{\omega_i}\right)^{\upsilon_j} + \Pi_{j=1}^{m}\left(\Pi_{i=1}^{n}\mathcal{T}_{ij}^{\omega_i}\right)^{\upsilon_j} + \Pi_{j=1}^{m}\left(\Pi_{i=1}^{n}\mathcal{L}_{ij}^{\omega_i}\right)^{\upsilon_j} = 3$$

Therefore,

$$0 \le \left\{\sqrt[q]{1-\Pi_{j=1}^{m}\left(\Pi_{i=1}^{n}\left(1-\mathcal{I}_{ij}^{q}\right)^{\omega_i}\right)^{\upsilon_j}}\right\}^{q} + \left\{\Pi_{j=1}^{m}\left(\Pi_{i=1}^{n}\mathcal{T}_{ij}^{\omega_i}\right)^{\upsilon_j}\right\}^{q} + \left\{\Pi_{j=1}^{m}\left(\Pi_{i=1}^{n}\mathcal{F}_{ij}^{\omega_i}\right)^{\upsilon_j}\right\}^{q} \le 3.$$

Hence, we proved the required result.

Example 2: Miss Marjan is charged with choosing the best life mate from among four people in the domain S = { $\hbar_1$, $\hbar_2$, $\hbar_3$, $\hbar_4$}, where $\hbar_1$ = Ali, $\hbar_2$ = Shoaib, $\hbar_3$ = Majeed and $\hbar_4$ = Ahmad. This selection is based on the parameters €= {g_1, g_2, g_3, g_4}, where g_1 = Trustworthy, g_2 = Loyal, g_3 = Dependable and g_4 = Compromising. Weights ω = {0.20,0.25,0.27,0.28} and υ = {0.5,0.16,0.14,0.20} respectively were assigned by the expert. The decision makers use q-RONS$_f$N$_s$, as indicated in Table 2, to deliver their assessments after weighing each alternative against these parameters.

Table 2. Represent q-RONS$_f$N$_s$ $\mathcal{A}_{b_{ij}} = (\mathcal{T}_{ij}, \mathcal{I}_{ij}, \mathcal{F}_{ij})$ for q ≥ 3.

S	g_1	g_2	g_3	g_4

$ħ_1$	(.77,.50,.30)	(.70,.40,.10)	(.55,.35,.20)	(.87,.42,.40)
$ħ_2$	(.67,.30,.30)	(.66,.25,.30)	(.77,.40,.30)	(.80,.10,.30)
$ħ_3$	(.61,.40,.30)	(.50,.10,.20)	(.87,.44,.10)	(.57,.22,.50)
$ħ_4$	(.88,.30,.20)	(.99,.10,.25)	(.44,.10,.30)	(.50,.33,.70)

By equation (10) we have

$$\text{q-RONS}_f\text{WA}\left(Ⱥ_{b_{11}}, Ⱥ_{b_{12}}, \ldots, Ⱥ_{b_{44}}\right) = \begin{pmatrix} \sqrt[q]{1-\prod_{j=1}^{m}\left(\prod_{i=1}^{n}\left(1-Ţ_{ij}^{q}\right)^{\omega_i}\right)^{\upsilon_j}}, \\ \prod_{j=1}^{m}\left(\prod_{i=1}^{n} ƚ_{ij}^{\omega_i}\right)^{\upsilon_j}, \\ \prod_{j=1}^{m}\left(\prod_{i=1}^{n} Ƒ_{ij}^{\omega_i}\right)^{\upsilon_j} \end{pmatrix}$$

= (0.7844,0.2792,0.2758)

3.3. Ordered Weighted Averaging Operator based on q-RONS set:

Consider the collection of q-RONFS$_f$N$_s$ $Ⱥ_{b_{ij}} = \left(Ţ_{ij}, ƚ_{ij}, Ƒ_{ij}\right)$ for variable i from 1 to n and j from 1 to m, having weight vector for experts x_i is ω_i and for parameters e_j is υ_j, with $\omega_i, \upsilon_j \in [0,1]$ such that $\sum_{i=1}^{n}\omega_i = 1$ and $\sum_{j=1}^{m}\upsilon_j = 1$ respectively. Then q-RONS$_f$OWA operator is defined by the mapping q-RONS$_f$OWA: $Đ^n \longrightarrow Đ$, where Đ represents the collection of q-RONFS$_f$N$_s$.

$$\text{q-RONFS}_f\text{OWA}\left(Ⱥ_{b_{11}}, Ⱥ_{b_{12}}, \ldots, Ⱥ_{b_{nm}}\right) = \oplus_{j=1}^{m} \upsilon_j \left(\oplus_{i=1}^{n} \omega_i Ⱥ_{\sigma b_{ij}}\right)$$

Theorem 3: Let $Ⱥ_{b_{ij}} = \left(Ţ_{ij}, ƚ_{ij}, Ƒ_{ij}\right)$ ∀ i,j rang from i to 1 and j to m, be the collection of q-RONS$_f$N$_s$. Then the aggregation result for q-RONS$_f$OWA operator is defined as:

$$\text{q-RONS}_f\text{OWA}\left(Ⱥ_{b_{11}}, Ⱥ_{b_{12}}, \ldots, Ⱥ_{b_{nm}}\right) = \oplus_{j=1}^{m} \upsilon_j \left(\oplus_{i=1}^{n} \omega_i Ⱥ_{\sigma b_{ij}}\right)$$

$$= \left(\sqrt[q]{1-\prod_{j=1}^{m}\left(\prod_{i=1}^{n}\left(1-Ţ_{\sigma ij}^{q}\right)^{\omega_i}\right)^{\upsilon_j}}, \prod_{j=1}^{m}\left(\prod_{i=1}^{n} ƚ_{\sigma ij}^{\omega_i}\right)^{\upsilon_j}, \prod_{j=1}^{m}\left(\prod_{i=1}^{n} Ƒ_{\sigma ij}^{\omega_i}\right)^{\upsilon_j}\right)$$

$$\text{q-RONS}_f\text{OWA}\left(Ⱥ_{b_{11}}, Ⱥ_{b_{12}}, \ldots, Ⱥ_{b_{nm}}\right) = \begin{pmatrix} \sqrt[q]{1-\prod_{j=1}^{m}\left(\prod_{i=1}^{n}\left(1-Ţ_{\sigma ij}^{q}\right)^{\omega_i}\right)^{\upsilon_j}}, \\ \prod_{j=1}^{m}\left(\prod_{i=1}^{n} ƚ_{\sigma ij}^{\omega_i}\right)^{\upsilon_j}, \\ \prod_{j=1}^{m}\left(\prod_{i=1}^{n} Ƒ_{\sigma ij}^{\omega_i}\right)^{\upsilon_j} \end{pmatrix} \quad (11)$$

Where WV of for expert x_i is ω_i and for parameters e_j is υ_j respectively, such that $\sum_{i=1}^{n}\omega_i = 1$ and $\sum_{j=1}^{m}\upsilon_j = 1$.

Equation (11) represent the q-RONWA operator.

Proof: Straightforward as the above result of " q-RONS$_f$WA " operator.

Example 3: From the above Example No.2 of "Table 2", we consider the collections of q-RONS$_f$N$_s$ $Ⱥ_{b_{ij}} = \left(Ţ_{ij}, ƚ_{ij}, Ƒ_{ij}\right)$, to get the tabular representation of $Ⱥ_{b_{ij}} = \left(Ţ_{\sigma ij}, ƚ_{\sigma ij}, Ƒ_{\sigma ij}\right)$ is expressed in "Table "3" by using the score function.

Table 3. Represent q-RONS$_f$N$_s$ $Ⱥ_{\sigma b_{ij}} = \left(Ţ_{\sigma ij}, ƚ_{\sigma ij}, Ƒ_{\sigma ij}\right)$ for q ≥ 3.

S	g_1	g_2	g_3	g_4
$ħ_1$	(0.88,0.30,0.20)	(0.99,0.10,0.25)	(0.87,0.44,0.10)	(0.87,0.42,0.40)
$ħ_2$	(0.77,0.50,0.30)	(0.70,0.40,0.10)	(0.77,0.40,0.30)	(0.80,0.10,0.30)

$\hbar_3$	(0.67,0.30,0.30)	(0.66,0.25,0.30)	(0.55,0.35,0.20)	(0.66,0.40,0.30)
$\hbar_4$	(0.61,0.40,0.30)	(0.51,0.40,0.30)	(0.44,0.10,0.30)	(0.57,0.22,0.5)

By equation (11) we have

$$\text{q-RONS}_f\text{OWA}(A_{b_{11}}, A_{b_{12}}, \ldots, A_{b_{nm}}) = \begin{pmatrix} \sqrt[q]{1-\prod_{j=1}^{m}\left(\prod_{i=1}^{n}\left(1-T_{\sigma ij}^{q}\right)^{\omega_i}\right)^{\upsilon_j}}, \\ \prod_{j=1}^{m}\left(\prod_{i=1}^{n} I_{\sigma ij}^{\omega_i}\right)^{\upsilon_j}, \\ \prod_{j=1}^{m}\left(\prod_{i=1}^{n} F_{\sigma ij}^{\omega_i}\right)^{\upsilon_j} \end{pmatrix}$$

$=(0.7657, 0.3084, 0.2725)$.

3.4. Weighted Geometric Operator Based on q-RONS set:

Consider the collection of q-RONFS$_f$N$_s$ $A_{b_{ij}}=(T_{ij}, I_{ij}, F_{ij})$ for i,j rang from i to 1 and j to m, having weight vector for experts x_i is ω_i and for parameters e_j is υ_j, with $\omega_i, \upsilon_j \in [0,1]$ such that $\sum_{i=1}^{n}\omega_i = 1$ and $\sum_{j=1}^{m}\upsilon_j = 1$ respectively. Then q-RONS$_f$WG operator is defined by the mapping q-RONS$_f$WG: $Đ^n \longrightarrow Đ$, where Đ represents the collection of q-RONFS$_f$N$_s$.

$$\text{q-RONS}_f\text{WG}\left(A_{b_{11}}, A_{b_{12}}, \ldots, A_{b_{nm}}\right) = \otimes_{j=1}^{m}\left(\otimes_{i=1}^{n} A_{b_{ij}}^{\omega_i}\right)^{\upsilon_j}$$

Theorem 4: Let $A_{b_{ij}}=(T_{ij}, I_{ij}, F_{ij})$ ∀ i,j rang from i to 1 and j to m, be the collection of q − RONS$_f$N$_s$. Then for q-RONS$_f$WG operator the aggregation result is defined:

$$\text{q-RONS}_f\text{WG}(A_{b_{11}}, A_{b_{12}}, \ldots, A_{b_{nm}}) = \otimes_{j=1}^{m}\left(\otimes_{i=1}^{n} A_{b_{ij}}^{\omega_i}\right)^{\upsilon_j}$$

$$=\left(\prod_{j=1}^{m}\left(\prod_{i=1}^{n} T_{ij}^{\omega_i}\right)^{\upsilon_j}, \sqrt[q]{1-\prod_{j=1}^{m}\left(\prod_{i=1}^{n}\left(1-I_{ij}^{q}\right)^{\omega_i}\right)^{\upsilon_j}}, \sqrt[q]{1-\prod_{j=1}^{m}\left(\prod_{i=1}^{n}\left(1-F_{ij}^{q}\right)^{\omega_i}\right)^{\upsilon_j}}\right)$$

$$\text{q-RONS}_f\text{WG}(A_{b_{11}}, A_{b_{12}}, \ldots, A_{b_{nm}}) = \begin{pmatrix} \prod_{j=1}^{m}\left(\prod_{i=1}^{n} T_{ij}^{\omega_i}\right)^{\upsilon_j}, \\ \sqrt[q]{1-\prod_{j=1}^{m}\left(\prod_{i=1}^{n}\left(1-I_{ij}^{q}\right)^{\omega_i}\right)^{\upsilon_j}}, \\ \sqrt[q]{1-\prod_{j=1}^{m}\left(\prod_{i=1}^{n}\left(1-F_{ij}^{q}\right)^{\omega_i}\right)^{\upsilon_j}} \end{pmatrix} \quad (12)$$

Where WV of for expert x_i is ω_i and for parameters e_j is υ_j respectively, such that $\sum_{i=1}^{n}\omega_i = 1$ and $\sum_{j=1}^{m}\upsilon_j = 1$.

Equation (12) represent the q-RONSWG operator.

Proof: Straightforward.

3.5. Order Weighted Geometric Operator Based on q-RONS set:

Consider the collection of q-RONFS$_f$N$_s$ $A_{b_{ij}}=(T_{ij}, I_{ij}, F_{ij})$ for i,j rang from i to 1 and j to m, having weight vector for experts x_i is ω_i and for parameters e_j is υ_j, with $\omega_i, \upsilon_j \in [0,1]$ such that $\sum_{i=1}^{n}\omega_i = 1$ and $\sum_{j=1}^{m}\upsilon_j = 1$ respectively. Then q-RONS$_f$OWG operator is defined by the mapping q-RONS$_f$OWG: $Đ^n \longrightarrow Đ$, where Đ represents the collection of q-RONFS$_f$N$_s$.

$$\text{q-RONS}_f\text{OWG}\left(A_{b_{11}}, A_{b_{12}}, \ldots, A_{b_{nm}}\right) = \otimes_{j=1}^{m}\left(\otimes_{i=1}^{n} A_{\sigma b_{ij}}^{\omega_i}\right)^{\upsilon_j}$$

Theorem 5: Let $A_{b_{ij}}=(T_{ij}, I_{ij}, F_{ij})$ for i,j rang from i to 1 and j to m, be the collection of q-RONS$_f$N$_s$. Then for q-RONS$_f$OWG operator the aggregation result is defined:

q-RONS$_f$OWG$\left(₳_{\flat_{11}},₳_{\flat_{12}},\ldots,₳_{\flat_{nm}}\right)$=$\otimes_{j=1}^{m}\left(\otimes_{i=1}^{n}₳_{\sigma\flat_{ij}}^{\omega_i}\right)^{\upsilon_j}$

$$=\left(\prod_{j=1}^{m}\left(\prod_{i=1}^{n}Ʈ_{\sigma ij}^{\omega_i}\right)^{\upsilon_j},\sqrt[q]{1-\prod_{j=1}^{m}\left(\prod_{i=1}^{n}\left(1-ɨ_{\sigma ij}^{q}\right)^{\omega_i}\right)^{\upsilon_j}},\sqrt[q]{1-\prod_{j=1}^{m}\left(\prod_{i=1}^{n}\left(1-Ƒ_{\sigma ij}^{q}\right)^{\omega_i}\right)^{\upsilon_j}}\right)$$

$$\text{q-RONS}_f\text{OWG}\left(₳_{\flat_{11}},₳_{\flat_{12}},\ldots,₳_{\flat_{nm}}\right)=\begin{pmatrix}\prod_{j=1}^{m}\left(\prod_{i=1}^{n}Ʈ_{\sigma ij}^{\omega_i}\right)^{\upsilon_j},\\ \sqrt[q]{1-\prod_{j=1}^{m}\left(\prod_{i=1}^{n}\left(1-ɨ_{\sigma ij}^{q}\right)^{\omega_i}\right)^{\upsilon_j}},\\ \sqrt[q]{1-\prod_{j=1}^{m}\left(\prod_{i=1}^{n}\left(1-Ƒ_{\sigma ij}^{q}\right)^{\omega_i}\right)^{\upsilon_j}}\end{pmatrix} \quad (13)$$

Where $₳_{\sigma\flat_{ij}}=\left(Ʈ_{\sigma ij},ɨ_{\sigma ij},Ƒ_{\sigma ij}\right)$ expressed the permutations of ith and j^{th} largest values of the collection of i×j of q-RONS$_f$N$_s$.

Equation (13) represent the q-RONSOWG operator.

Proof: Straightforward.

3.6. Some special properties related based on proposed AOs:

The following properties are held by $q-RONS_fWA$, $q-RONS_fOWA$, $q-RONS_fWG$ and $q-RONS_fOWG$ operators.

Property 1 (Idempotency):

a. If $₳_{\flat_{ij}}$= $₦_\flat$, where $₦_\flat=(₩,₵,₱)$, then q-RONS$_f$WA $\left(₳_{\flat_{11}},₳_{\flat_{12}},\ldots,₳_{\flat_{nm}}\right)$ = $₦_\flat$.

b. If $₳_{\flat_{ij}}$= $₦_\flat$, where $₦_\flat=(₩,₵,₱)$, then q-RONS$_f$OWA $\left(₳_{\flat_{11}},₳_{\flat_{12}},\ldots,₳_{\flat_{nm}}\right)$ = $₦_\flat$.

c. If $₳_{\flat_{ij}}$= $₦_\flat$, where $₦_\flat=(₩,₵,₱)$, then q-RONS$_f$WG $\left(₳_{\flat_{11}},₳_{\flat_{12}},\ldots,₳_{\flat_{nm}}\right)$ = $₦_\flat$.

d. If $₳_{\flat_{ij}}$= $₦_\flat$, where $₦_\flat=(₩,₵,₱)$, then q-RONS$_f$OWG $\left(₳_{\flat_{11}},₳_{\flat_{12}},\ldots,₳_{\flat_{nm}}\right)$ = $₦_\flat$.

Proof (a): It is given that $₳_{\flat_{ij}}=₦_\flat=(₩,₵,₱)$, then

$$\text{q-RONS}_f\text{WA}\left(₳_{\flat_{11}},₳_{\flat_{12}},\ldots,₳_{\flat_{nm}}\right)=\begin{pmatrix}\sqrt[q]{1-\prod_{j=1}^{m}\left(\prod_{i=1}^{n}\left(1-Ʈ_{ij}^{q}\right)^{\omega_i}\right)^{\upsilon_j}},\\ \prod_{j=1}^{m}\left(\prod_{i=1}^{n}ɨ_{ij}^{\omega_i}\right)^{\upsilon_j},\\ \prod_{j=1}^{m}\left(\prod_{i=1}^{n}Ƒ_{ij}^{\omega_i}\right)^{\upsilon_j}\end{pmatrix}$$

$$=\begin{pmatrix}\sqrt[q]{1-\prod_{j=1}^{m}(\prod_{i=1}^{n}(1-₩^q)^{\omega_i})^{\upsilon_j}},\\ \prod_{j=1}^{m}(\prod_{i=1}^{n}₵^q)^{\upsilon_j},\\ \prod_{j=1}^{m}(\prod_{i=1}^{n}₱^q)^{\upsilon_j}\end{pmatrix}\Rightarrow\left(\sqrt[q]{1-(1-₩^q)},₵^q,₱^q\right)$$

Hence, q-RONS$_f$WA $\left(₳_{\flat_{11}},₳_{\flat_{12}},\ldots,₳_{\flat_{nm}}\right)$ = $₦_\flat$.

Proof (b),(c),(d): Straightforward as above.

Property 2 (Boundedness):

a. If $₳_{\flat_{ij}}^{+}=\left\{\max_j\max_i(Ʈ_{ij}),\min_j\min_i(ɨ_{ij}),\min_j\min_i(Ƒ_{ij})\right\}$ and $₳_{\flat_{ij}}^{-}=\left\{\min_j\min_i(Ʈ_{ij}),\max_j\max_i(ɨ_{ij}),\max_j\max_i(Ƒ_{ij})\right\}$, then $₳_{\flat_{ij}}^{-}\le q-\text{RONS}_f\text{WA}\left(₳_{\flat_{11}},₳_{\flat_{12}},\ldots,₳_{\flat_{nm}}\right)\le ₳_{\flat_{ij}}^{+}$.

b. If $\text{₳}^{+}_{\text{ƀ}_{ij}} = \left\{ \max\limits_{j} \max\limits_{i} (\text{Ʈ}_{ij}), \min\limits_{j} \min\limits_{i} (\text{ɨ}_{ij}), \min\limits_{j} \min\limits_{i} (\text{Ƒ}_{ij}) \right\}$ and $\text{₳}^{-}_{\text{ƀ}_{ij}} = \left\{ \min\limits_{j} \min\limits_{i} (\text{Ʈ}_{ij}), \max\limits_{j} \max\limits_{i} (\text{ɨ}_{ij}), \max\limits_{j} \max\limits_{i} (\text{Ƒ}_{ij}) \right\}$, then $\text{₳}^{-}_{\text{ƀ}_{ij}} \leq q - \mathrm{RONS_fOWA}\left(\text{₳}_{\text{ƀ}_{11}}, \text{₳}_{\text{ƀ}_{12}}, \ldots, \text{₳}_{\text{ƀ}_{nm}}\right) \leq \text{₳}^{+}_{\text{ƀ}_{ij}}$.

c. If $\text{₳}^{+}_{\text{ƀ}_{ij}} = \left\{ \max\limits_{j} \max\limits_{i} (\text{Ʈ}_{ij}), \min\limits_{j} \min\limits_{i} (\text{ɨ}_{ij}), \min\limits_{j} \min\limits_{i} (\text{Ƒ}_{ij}) \right\}$ and $\text{₳}^{-}_{\text{ƀ}_{ij}} = \left\{ \min\limits_{j} \min\limits_{i} (\text{Ʈ}_{ij}), \max\limits_{j} \max\limits_{i} (\text{ɨ}_{ij}), \max\limits_{j} \max\limits_{i} (\text{Ƒ}_{ij}) \right\}$, then $\text{₳}^{-}_{\text{ƀ}_{ij}} \leq q - \mathrm{RONS_fWG}\left(\text{₳}_{\text{ƀ}_{11}}, \text{₳}_{\text{ƀ}_{12}}, \ldots, \text{₳}_{\text{ƀ}_{nm}}\right) \leq \text{₳}^{+}_{\text{ƀ}_{ij}}$.

d. If $\text{₳}^{+}_{\text{ƀ}_{ij}} = \left\{ \max\limits_{j} \max\limits_{i} (\text{Ʈ}_{ij}), \min\limits_{j} \min\limits_{i} (\text{ɨ}_{ij}), \min\limits_{j} \min\limits_{i} (\text{Ƒ}_{ij}) \right\}$ and $\text{₳}^{-}_{\text{ƀ}_{ij}} = \left\{ \min\limits_{j} \min\limits_{i} (\text{Ʈ}_{ij}), \max\limits_{j} \max\limits_{i} (\text{ɨ}_{ij}), \max\limits_{j} \max\limits_{i} (\text{Ƒ}_{ij}) \right\}$, then $\text{₳}^{-}_{\text{ƀ}_{ij}} \leq q - \mathrm{RONS_fOWG}\left(\text{₳}_{\text{ƀ}_{11}}, \text{₳}_{\text{ƀ}_{12}}, \ldots, \text{₳}_{\text{ƀ}_{nm}}\right) \leq \text{₳}^{+}_{\text{ƀ}_{ij}}$.

Proof (a): We know that

$$\text{₳}^{-}_{\text{ƀ}_{ij}} = \left\{ \min\limits_{j} \min\limits_{i} (\text{Ʈ}_{ij}), \max\limits_{j} \max\limits_{i} (\text{ɨ}_{ij}), \max\limits_{j} \max\limits_{i} (\text{Ƒ}_{ij}) \right\}$$

and

$$\text{₳}^{+}_{\text{ƀ}_{ij}} = \left\{ \max\limits_{j} \max\limits_{i} (\text{Ʈ}_{ij}), \min\limits_{j} \min\limits_{i} (\text{ɨ}_{ij}), \min\limits_{j} \min\limits_{i} (\text{Ƒ}_{ij}) \right\},$$

To show that,

$$\text{₳}^{-}_{\text{ƀ}_{ij}} \leq q - \mathrm{RONS_fWA}\left(\text{₳}_{\text{ƀ}_{11}}, \text{₳}_{\text{ƀ}_{12}}, \ldots, \text{₳}_{\text{ƀ}_{nm}}\right) \leq \text{₳}^{+}_{\text{ƀ}_{ij}}.$$

$$\Rightarrow \min\limits_{j} \min\limits_{i} \{\text{Ʈ}_{ij}\} \leq \text{Ʈ}_{ij} \leq \max\limits_{j} \max\limits_{i} \{\text{Ʈ}_{ij}\}$$

$$\Leftrightarrow 1 - \max\limits_{j} \max\limits_{i} \{\text{Ʈ}_{ij}^{q}\} \leq 1 - \text{Ʈ}_{ij}^{q} \leq 1 - \min\limits_{j} \min\limits_{i} \{\text{Ʈ}_{ij}^{q}\}$$

$$\Leftrightarrow \Pi_{j=1}^{m}\left(\Pi_{i=1}^{n}\left(1 - \max\limits_{j} \max\limits_{i} \{\text{Ʈ}_{ij}^{q}\}\right)^{\omega_i}\right)^{\upsilon_j} \leq \Pi_{j=1}^{m}\left(\Pi_{i=1}^{n}\left(1 - \text{Ʈ}_{ij}^{q}\right)^{\omega_i}\right)^{\upsilon_j} \leq \Pi_{j=1}^{m}\left(\Pi_{i=1}^{n}\left(1 - \min\limits_{j} \min\limits_{i} \{\text{Ʈ}_{ij}^{q}\}\right)^{\omega_i}\right)^{\upsilon_j}$$

$$\Leftrightarrow \left(\left(1 - \max\limits_{j} \max\limits_{i} \{\text{Ʈ}_{ij}^{q}\}\right)^{\sum_{i=1}^{n}\omega_i}\right)^{\sum_{j=1}^{m}\upsilon_j} \leq \Pi_{j=1}^{m}\left(\Pi_{i=1}^{n}\left(1 - \text{Ʈ}_{ij}^{q}\right)^{\omega_i}\right)^{\upsilon_j} \leq \left(\left(1 - \min\limits_{j} \min\limits_{i} \{\text{Ʈ}_{ij}^{q}\}\right)^{\sum_{i=1}^{n}\omega_i}\right)^{\sum_{i=1}^{m}\upsilon_j}$$

$$\Leftrightarrow \left(1 - \max\limits_{j} \max\limits_{i} \{\text{Ʈ}_{ij}^{q}\}\right) \leq \Pi_{j=1}^{m}\left(\Pi_{i=1}^{n}\left(1 - \text{Ʈ}_{ij}^{q}\right)^{\omega_i}\right)^{\upsilon_j} \leq \left(1 - \min\limits_{j} \min\limits_{i} \{\text{Ʈ}_{ij}^{q}\}\right)$$

$$\Leftrightarrow 1 - \left(1 - \max\limits_{j} \max\limits_{i} \{\text{Ʈ}_{ij}^{q}\}\right) \leq 1 - \Pi_{j=1}^{m}\left(\Pi_{i=1}^{n}\left(1 - \text{Ʈ}_{ij}^{q}\right)^{\omega_i}\right)^{\upsilon_j} \leq 1 - \left(1 - \min\limits_{j} \min\limits_{i} \{\text{Ʈ}_{ij}^{q}\}\right)$$

$$\Leftrightarrow \min\limits_{j} \min\limits_{i} \{\text{Ʈ}_{ij}\} \leq \sqrt[q]{1 - \Pi_{j=1}^{m}\left(\Pi_{i=1}^{n}\left(1 - \text{Ʈ}_{ij}^{q}\right)^{\omega_i}\right)^{\upsilon_j}} \leq \max\limits_{j} \max\limits_{i} \{\text{Ʈ}_{ij}\}$$

Next, we have

$$\Leftrightarrow \min_j \min_i \{\text{ɨ}_{\mathrm{ij}}\} \le \text{ɨ}_{\mathrm{ij}} \le \max_j \max_i \{\text{ɨ}_{\mathrm{ij}}\}$$

$$\Leftrightarrow \Pi_{j=1}^{m}\left(\Pi_{i=1}^{n}\left(\min_j \min_i \{\text{ɨ}_{\mathrm{ij}}\}\right)^{\omega_i}\right)^{\upsilon_j} \le \Pi_{j=1}^{m}\left(\Pi_{i=1}^{n}(\text{ɨ}_{\mathrm{ij}})^{\omega_i}\right)^{\upsilon_j} \le \Pi_{j=1}^{m}\left(\Pi_{i=1}^{n}\left(\max_j \max_i \{\text{ɨ}_{\mathrm{ij}}\}\right)^{\omega_i}\right)^{\upsilon_j}$$

$$\Leftrightarrow \left(\left(\min_j \min_i \{\text{ɨ}_{\mathrm{ij}}\}\right)^{\sum_{i=1}^{n}\omega_i}\right)^{\sum_{j=1}^{m}\upsilon_j} \le \Pi_{j=1}^{m}\left(\Pi_{i=1}^{n}(\text{ɨ}_{\mathrm{ij}})^{\omega_i}\right)^{\upsilon_j} \le \left(\left(\max_j \max_i \{\text{ɨ}_{\mathrm{ij}}\}\right)^{\sum_{i=1}^{n}\omega_i}\right)^{\sum_{j=1}^{m}\upsilon_j}$$

$$\Leftrightarrow \min_j \min_i \{\text{ɨ}_{\mathrm{ij}}\} \le \Pi_{j=1}^{m}\left(\Pi_{i=1}^{n}(1-\text{ɨ}_{ij}^{q})^{\omega_i}\right)^{\upsilon_j} \le \max_j \max_i \{\text{ɨ}_{\mathrm{ij}}\}$$

And finally

$$\Leftrightarrow \min_j \min_i \{\text{Ƒ}_{\mathrm{ij}}\} \le \text{Ƒ}_{\mathrm{ij}} \le \max_j \max_i \{\text{Ƒ}_{\mathrm{ij}}\}$$

$$\Leftrightarrow \Pi_{j=1}^{m}\left(\Pi_{i=1}^{n}\left(\min_j \min_i \{\text{Ƒ}_{\mathrm{ij}}\}\right)^{\omega_i}\right)^{\upsilon_j} \le \Pi_{j=1}^{m}\left(\Pi_{i=1}^{n}(\text{Ƒ}_{\mathrm{ij}})^{\omega_i}\right)^{\upsilon_j} \le \Pi_{j=1}^{m}\left(\Pi_{i=1}^{n}\left(\max_j \max_i \{\text{Ƒ}_{\mathrm{ij}}\}\right)^{\omega_i}\right)^{\upsilon_j}$$

$$\Leftrightarrow \left(\left(\min_j \min_i \{\text{Ƒ}_{\mathrm{ij}}\}\right)^{\sum_{i=1}^{n}\omega_i}\right)^{\sum_{j=1}^{m}\upsilon_j} \le \Pi_{j=1}^{m}\left(\Pi_{i=1}^{n}(\text{Ƒ}_{\mathrm{ij}})^{\omega_i}\right)^{\upsilon_j} \le \left(\left(\max_j \max_i \{\text{Ƒ}_{\mathrm{ij}}\}\right)^{\sum_{i=1}^{n}\omega_i}\right)^{\sum_{j=1}^{m}\upsilon_j}$$

$$\Leftrightarrow \min_j \min_i \{\text{Ƒ}_{\mathrm{ij}}\} \le \Pi_{j=1}^{m}\left(\Pi_{i=1}^{n}(\text{Ƒ}_{\mathrm{ij}})^{\omega_i}\right)^{\upsilon_j} \le \max_j \max_i \{\text{Ƒ}_{\mathrm{ij}}\}$$

Therefore,

$$\Leftrightarrow \min_j \min_i \{\text{Ʈ}_{\mathrm{ij}}\} \le \sqrt[q]{1-\Pi_{j=1}^{m}\left(\Pi_{i=1}^{n}\left(1-\text{Ʈ}_{ij}^{q}\right)^{\omega_i}\right)^{\upsilon_j}} \le \max_j \max_i \{\text{Ʈ}_{\mathrm{ij}}\}$$

$$\Leftrightarrow \min_j \min_i \{\text{ɨ}_{\mathrm{ij}}\} \le \Pi_{j=1}^{m}\left(\Pi_{i=1}^{n}(1-\text{ɨ}_{ij}^{q})^{\omega_i}\right)^{\upsilon_j} \le \max_j \max_i \{\text{ɨ}_{\mathrm{ij}}\}$$

$$\Leftrightarrow \min_j \min_i \{\text{Ƒ}_{\mathrm{ij}}\} \le \Pi_{j=1}^{m}\left(\Pi_{i=1}^{n}(\text{Ƒ}_{\mathrm{ij}})^{\omega_i}\right)^{\upsilon_j} \le \max_j \max_i \{\text{Ƒ}_{\mathrm{ij}}\}$$

Let δ =q-RONS$_{\mathrm{f}}$WA $\left(\text{Ⱥ}_{\mathfrak{b}_{11}}, \text{Ⱥ}_{\mathfrak{b}_{12}}, \ldots., \text{Ⱥ}_{\mathfrak{b}_{\mathrm{nm}}}\right) = (\text{Ʈ}_\delta, \text{ɨ}_\delta, \text{Ƒ}_\delta)$, then by the score function

$$S(\delta) = \text{Ʈ}_\delta^{q} - \text{ɨ}_\delta^{q} - \text{Ƒ}_\delta^{q} + \left(\frac{e^{\text{Ʈ}_\delta^{q} - \text{ɨ}_\delta^{q} - \text{Ƒ}_\delta^{q}}}{e^{\text{Ʈ}_\delta^{q} - \text{ɨ}_\delta^{q} - \text{Ƒ}_{\delta+1}^{q}}} - \frac{1}{2}\right)\pi_\delta^{q}$$

$$\leq \left(\max_j \max_i \{\text{Ʈ}_{ij}\}\right)^q - \left(\min_j \min_i \{\text{ɨ}_{ij}\}\right)^q - \left(\min_j \min_i \{\text{Ƒ}_{ij}\}\right)^q + \left(\frac{e^{\left(\max_j \max_i \{\text{Ʈ}_{ij}\}\right)^q - \left(\min_j \min_i \{\text{ɨ}_{ij}\}\right)^q - \left(\min_j \min_i \{\text{Ƒ}_{ij}\}\right)^q}}{e^{\left(\max_j \max_i \{\text{Ʈ}_{ij}\}\right)^q - \left(\min_j \min_i \{\text{ɨ}_{ij}\}\right)^q - \left(\min_j \min_i \{\text{Ƒ}_{ij}\}\right)^q}+1} - \frac{1}{2}\right)\pi^q_{\text{Ñ}^+_{\text{Ϸ}_{ij}}}$$

$= S\left(\text{Ⱥ}^+_{\text{Ϸ}_{ij}}\right)$

$\Rightarrow S(\delta) \leq S\left(\text{Ⱥ}^+_{\text{Ϸ}_{ij}}\right)$

and

$$\Leftrightarrow S(\delta) = \text{Ʈ}^q_\delta - \text{ɨ}^q_\delta - \text{Ƒ}^q_\delta + \left(\frac{e^{\text{Ʈ}^q_\delta - \text{ɨ}^q_\delta - \text{Ƒ}^q_\delta}}{e^{\text{Ʈ}^q_\delta - \text{ɨ}^q_\delta - \text{Ƒ}^q_\delta}+1} - \frac{1}{2}\right)\pi^q_\delta$$

$$\geq \left(\min_j \min_i \{\text{Ʈ}_{ij}\}\right)^q - \left(\max_j \max_i \{\text{ɨ}_{ij}\}\right)^q - \left(\max_j \max_i \{\text{Ƒ}_{ij}\}\right)^q + \left(\frac{e^{\left(\min_j \min_i \{\text{Ʈ}_{ij}\}\right)^q - \left(\max_j \max_i \{\text{ɨ}_{ij}\}\right)^q - \left(\max_j \max_i \{\text{Ƒ}_{ij}\}\right)^q}}{e^{\left(\min_j \min_i \{\text{Ʈ}_{ij}\}\right)^q - \left(\max_j \max_i \{\text{ɨ}_{ij}\}\right)^q - \left(\max_j \max_i \{\text{Ƒ}_{ij}\}\right)^q}+1} - \frac{1}{2}\right)\pi^q_{\text{Ⱥ}^-_{\text{Ϸ}_{ij}}}$$

$= S\left(\text{Ⱥ}^-_{\text{Ϸ}_{ij}}\right)$

$\Rightarrow S(\delta) \geq S\left(\text{Ⱥ}^-_{\text{Ϸ}_{ij}}\right)$

We encounter the following cases, with the above methodology

i. By the comparison of two q-RONS$_f$N$_s$, we obtain $\text{Ⱥ}^-_{\text{Ϸ}_{ij}} < q - \text{RONS}_f\text{WA}\left(\text{Ⱥ}_{\ddot{e}_{11}}, \text{Ⱥ}_{\ddot{e}_{12}}, \ldots, \text{Ⱥ}_{\ddot{e}_{nm}}\right) < \text{Ⱥ}^+_{\text{Ϸ}_{ij}}$ if we have $S(\delta) \leq S\left(\text{Ⱥ}^+_{\text{Ϸ}_{ij}}\right)$ and $S(\delta) \geq S\left(\text{Ⱥ}^-_{\text{Ϸ}_{ij}}\right)$.

ii. If $S(\delta) = S\left(\text{Ⱥ}^+_{\text{Ϸ}_{ij}}\right)$, then

$$\text{Ʈ}^q_\delta - \text{ɨ}^q_\delta - \text{Ƒ}^q_\delta + \left(\frac{e^{\text{Ʈ}^q_\delta - \text{ɨ}^q_\delta - \text{Ƒ}^q_\delta}}{e^{\text{Ʈ}^q_\delta - \text{ɨ}^q_\delta - \text{Ƒ}^q_\delta}+1} - \frac{1}{2}\right)\pi^q_\delta$$

$$= \left(\max_j \max_i \{\text{Ʈ}_{ij}\}\right)^q - \left(\min_j \min_i \{\text{ɨ}_{ij}\}\right)^q - \left(\min_j \min_i \{\text{Ƒ}_{ij}\}\right)^q + \left(\frac{e^{\left(\max_j \max_i \{\text{Ʈ}_{ij}\}\right)^q - \left(\min_j \min_i \{\text{ɨ}_{ij}\}\right)^q - \left(\min_j \min_i \{\text{Ƒ}_{ij}\}\right)^q}}{e^{\left(\max_j \max_i \{\text{Ʈ}_{ij}\}\right)^q - \left(\min_j \min_i \{\text{ɨ}_{ij}\}\right)^q - \left(\min_j \min_i \{\text{Ƒ}_{ij}\}\right)^q}+1} - \frac{1}{2}\right)\pi^q_{\text{Ⱥ}^+_{\text{Ϸ}_{ij}}}$$

Using the above inequalities, we get

$$\Rightarrow \mathrm{T}_\delta = \max_j \max_i \{\mathrm{T}_{ij}\},\ \mathrm{I}_\delta = \min_j \min_i (\mathrm{I}_{ij}), \mathrm{F}_\delta = \min_j \min_i (\mathrm{F}_{ij})$$

$$\Rightarrow \pi_\delta^q = \pi^q_{\mathrm{A}^+_{\mathrm{b}ij}}$$

$$\Rightarrow \text{q-RONS}_f\text{WA}\left(\mathrm{A}_{\mathrm{b}_{11}}, \mathrm{A}_{\mathrm{b}_{12}}, \ldots, \mathrm{A}_{\mathrm{b}_{nm}}\right) = \mathrm{A}^+_{\mathrm{b}ij}$$

iii. sIf $S(\delta) = S\left(\mathrm{A}^-_{\mathrm{b}ij}\right)$, then

$$\mathrm{T}_\delta^q - \mathrm{I}_\delta^q - \mathrm{F}_\delta^q + \left(\frac{e^{\mathrm{T}_\delta^q - \mathrm{I}_\delta^q - \mathrm{F}_\delta^q}}{e^{\mathrm{T}_\delta^q - \mathrm{I}_\delta^q - \mathrm{F}_\delta^q}+1} - \frac{1}{2}\right)\pi_\delta^q$$

$$= \left(\min_j \min_i \{\mathrm{T}_{ij}\}\right)^q - \left(\max_j \max_i \{\mathrm{I}_{ij}\}\right)^q - \left(\max_j \max_i \{\mathrm{F}_{ij}\}\right)^q +$$
$$\left(\frac{e^{\left(\min_j \min_i \{\mathrm{T}_{ij}\}\right)^q - \left(\max_j \max_i \{\mathrm{I}_{ij}\}\right)^q - \left(\max_j \max_i \{\mathrm{F}_{ij}\}\right)^q}}{e^{\left(\min_j \min_i \{\mathrm{T}_{ij}\}\right)^q - \left(\max_j \max_i \{\mathrm{I}_{ij}\}\right)^q - \left(\max_j \max_i \{\mathrm{F}_{ij}\}\right)^q}+1} - \frac{1}{2}\right)\pi^q_{\mathrm{A}^-_{\mathrm{b}ij}}$$

then by the above inequalities, we get

$$\Rightarrow \mathrm{T}_\delta = \min_j \min_i (\mathrm{T}_{ij}),,\ \mathrm{I}_\delta = \max_j \max_i (\mathrm{I}_{ij}), \mathrm{F}_\delta = \max_j \max_i (\mathrm{F}_{ij})$$

$$\Rightarrow \pi_\delta^q = \pi^q_{\mathrm{A}^-_{\mathrm{b}ij}}$$

$$\Rightarrow \text{q-RONS}_f\text{WA}\left(\mathrm{A}_{\mathrm{b}_{11}}, \mathrm{A}_{\mathrm{b}_{12}}, \ldots, \mathrm{A}_{\mathrm{b}_{nm}}\right) = \mathrm{A}^-_{\mathrm{b}ij}$$

Hence

$$\mathrm{A}^-_{\mathrm{b}ij} \le \text{q} - \text{RONS}_f\text{WA}\left(\mathrm{A}_{\mathrm{b}_{11}}, \mathrm{A}_{\mathrm{b}_{12}}, \ldots, \mathrm{A}_{\mathrm{b}_{nm}}\right) \le \mathrm{A}^+_{\mathrm{b}ij}.$$

Proof (b),(c),(d): Straightforward as above.

Property 3 (Monotonicity):

a. If $\mathrm{N}_{\mathrm{b}ij} = \left(\mathrm{W}_{ij}, \mathrm{G}_{ij}, \mathrm{P}_{ij}\right)$ be a q-RONS$_f$N$_s$ such that $\mathrm{T}_{ij} \le \mathrm{W}_{ij}$, $\mathrm{I}_{ij} \ge \mathrm{G}_{ij}$, $\mathrm{F}_{ij} \ge \mathrm{P}_{ij}$ then q-RONS$_f$WA $\left(\mathrm{A}_{\mathrm{b}_{11}}, \mathrm{A}_{\mathrm{b}_{12}}, \ldots, \mathrm{A}_{\mathrm{b}_{nm}}\right) \le$ q-RONFS$_f$WA $\left(\mathrm{N}_{\mathrm{b}_{11}}, \mathrm{N}_{\mathrm{b}_{12}}, \ldots, \mathrm{N}_{\mathrm{b}_{nm}}\right)$.

b. If $\mathrm{N}_{\mathrm{b}ij} = \left(\mathrm{W}_{ij}, \mathrm{G}_{ij}, \mathrm{P}_{ij}\right)$ be a q-RONS$_f$N$_s$ such that $\mathrm{T}_{ij} \le \mathrm{W}_{ij}$, $\mathrm{I}_{ij} \ge \mathrm{G}_{ij}$, $\mathrm{F}_{ij} \ge \mathrm{P}_{ij}$ then q-RONS$_f$OWA $\left(\mathrm{A}_{\mathrm{b}_{11}}, \mathrm{A}_{\mathrm{b}_{12}}, \ldots, \mathrm{A}_{\mathrm{b}_{nm}}\right) \le$ q-RONFS$_f$OWA $\left(\mathrm{N}_{\mathrm{b}_{11}}, \mathrm{N}_{\mathrm{b}_{12}}, \ldots, \mathrm{N}_{\mathrm{b}_{nm}}\right)$.

c. If $\mathrm{N}_{\mathrm{b}ij} = \left(\mathrm{W}_{ij}, \mathrm{G}_{ij}, \mathrm{P}_{ij}\right)$ be a q-RONS$_f$N$_s$ such that $\mathrm{T}_{ij} \le \mathrm{W}_{ij}$, $\mathrm{I}_{ij} \ge \mathrm{G}_{ij}$, $\mathrm{F}_{ij} \ge \mathrm{P}_{ij}$ then q-RONS$_f$WG $\left(\mathrm{A}_{\mathrm{b}_{11}}, \mathrm{A}_{\mathrm{b}_{12}}, \ldots, \mathrm{A}_{\mathrm{b}_{nm}}\right) \le$ q-RONFS$_f$WG $\left(\mathrm{N}_{\mathrm{b}_{11}}, \mathrm{N}_{\mathrm{b}_{12}}, \ldots, \mathrm{N}_{\mathrm{b}_{nm}}\right)$.

d. If $\mathrm{N}_{\mathrm{b}ij} = \left(\mathrm{W}_{ij}, \mathrm{G}_{ij}, \mathrm{P}_{ij}\right)$ be a q-RONS$_f$N$_s$ such that $\mathrm{T}_{ij} \le \mathrm{W}_{ij}$, $\mathrm{I}_{ij} \ge \mathrm{G}_{ij}$, $\mathrm{F}_{ij} \ge \mathrm{P}_{ij}$ then q-RONS$_f$OWG $\left(\mathrm{A}_{\mathrm{b}_{11}}, \mathrm{A}_{\mathrm{b}_{12}}, \ldots, \mathrm{A}_{\mathrm{b}_{nm}}\right) \le$ q-RONFS$_f$OWG $\left(\mathrm{N}_{\mathrm{b}_{11}}, \mathrm{N}_{\mathrm{b}_{12}}, \ldots, \mathrm{N}_{\mathrm{b}_{nm}}\right)$

Proof (a): Since $\mathrm{T}_{ij} \le \mathrm{W}_{ij}, \mathrm{I}_{ij} \ge \mathrm{G}_{ij}$ and $\mathrm{F}_{ij} \ge \mathrm{P}_{ij}$, then

$\Rightarrow \text{Ƭ}_{ij} \leq \text{₩}_{ij} \Rightarrow 1\text{–}\text{₩}_{ij} \leq 1\text{–}\text{Ƭ}_{ij} \Rightarrow 1\text{–}\text{₩}_{ij}^{q} \leq 1\text{–}\text{Ƭ}_{ij}^{q}$

$\Rightarrow \prod_{j=1}^{m}\left(\prod_{i=1}^{n}\left(1-\text{₩}_{ij}^{q}\right)^{\omega_i}\right)^{\upsilon_j} \leq \prod_{j=1}^{m}\left(\prod_{i=1}^{n}\left(1-\text{Ƭ}_{ij}^{q}\right)^{\omega_i}\right)^{\upsilon_j}$

$\Rightarrow 1-\prod_{j=1}^{m}\left(\prod_{i=1}^{n}\left(1-\text{Ƭ}_{ij}^{q}\right)^{\omega_i}\right)^{\upsilon_j} \leq 1-\prod_{j=1}^{m}\left(\prod_{i=1}^{n}\left(1-\text{₩}_{ij}^{q}\right)^{\omega_i}\right)^{\upsilon_j}$

$\Rightarrow \sqrt[q]{1-\prod_{j=1}^{m}\left(\prod_{i=1}^{n}\left(1-\text{Ƭ}_{ij}^{q}\right)^{\omega_i}\right)^{\upsilon_j}} \leq \sqrt[q]{1-\prod_{j=1}^{m}\left(\prod_{i=1}^{n}\left(1-\text{₩}_{ij}^{q}\right)^{\omega_i}\right)^{\upsilon_j}}$

next $\text{Ɨ}_{ij} \geq \text{₵}_{ij}$

$\Rightarrow \prod_{i=1}^{n}\left(\text{Ɨ}_{ij}\right)^{\omega_i} \geq \prod_{i=1}^{n}\left(\text{₵}_{ij}\right)^{\omega_i}$

$\Rightarrow \prod_{j=1}^{m}\left(\prod_{i=1}^{n}\left(\text{Ɨ}_{ij}\right)^{\omega_i}\right)^{\upsilon_j} \geq \prod_{j=1}^{m}\left(\prod_{i=1}^{n}\left(\text{₵}_{ij}\right)^{\omega_i}\right)^{\upsilon_j}$

and $\text{Ƒ}_{ij} \geq \text{₱}_{ij}$

$\Rightarrow \prod_{i=1}^{n}\left(\text{Ƒ}_{ij}\right)^{\omega_i} \geq \prod_{i=1}^{n}\left(\text{₱}_{ij}\right)^{\omega_i}$

$\Rightarrow \prod_{j=1}^{m}\left(\prod_{i=1}^{n}\left(\text{Ƒ}_{ij}\right)^{\omega_i}\right)^{\upsilon_j} \geq \prod_{j=1}^{m}\left(\prod_{i=1}^{n}\left(\text{₱}_{ij}\right)^{\omega_i}\right)^{\upsilon_j}$

Suppose that $\delta_{\text{₳}}$=q-RONS$_f$WA $\left(\text{₳}_{\mathfrak{b}_{11}}, \text{₳}_{\mathfrak{b}_{12}}, \ldots, \text{₳}_{\mathfrak{b}_{nm}}\right) = \left(\text{Ƭ}_{\delta_{\text{₳}}}, \text{Ɨ}_{\delta_{\text{₳}}}, \text{Ƒ}_{\delta_{\text{₳}}}\right)$ and

$\delta_{\text{₦}}$ = q-RONS$_f$WA $\left(\text{₦}_{\mathfrak{b}_{11}}, \text{₦}_{\mathfrak{b}_{12}}, \ldots, \text{₦}_{\mathfrak{b}_{nm}}\right) = \left(\text{₩}_{\delta_{\text{₦}}}, \text{₵}_{\delta_{\text{₦}}}, \text{₱}_{\delta_{\text{₦}}}\right)$

Now, from the above equation, we have

$\text{Ƭ}_{ij} \leq \text{₩}_{ij}, \text{Ɨ}_{ij} \geq \text{₵}_{ij}$ and $\text{Ƒ}_{ij} \geq \text{₱}_{ij}$

$S(\delta_{\text{₳}}) \leq S(\delta_{\text{₦}})$, by using the score value

The following cases are encounter:

I. By the comparison of two q-ROP soft numbers, if $S(\delta_{\text{₳}}) < S(\delta_{\text{₦}})$, then q-RONS$_f$WA $\left(\text{₳}_{\mathfrak{b}_{11}}, \text{₳}_{\mathfrak{b}_{12}}, \ldots, \text{₳}_{\mathfrak{b}_{nm}}\right) <$ q – RONS$_f$WA $\left(\text{₦}_{\mathfrak{b}_{11}}, \text{₦}_{\mathfrak{b}_{12}}, \ldots, \text{₦}_{\mathfrak{b}_{nm}}\right)$.

II. If $S(\delta_{\text{₳}}) = S(\delta_{\mathfrak{R}})$, where

$$S(\delta_{\text{₳}}) = \text{Ƭ}_{\delta_{\text{₳}}}^{q} - \text{Ɨ}_{\delta_{\text{₳}}}^{q} - \text{Ƒ}_{\delta_{\text{₳}}}^{q} + \left(\frac{e^{\text{Ƭ}_{\delta_{\text{₳}}}^{q} - \text{Ɨ}_{\delta_{\text{₳}}}^{q} - \text{Ƒ}_{\delta_{\text{₳}}}^{q}}}{e^{\text{Ƭ}_{\delta_{\text{₳}}}^{q} - \text{Ɨ}_{\delta_{\text{₳}}}^{q} - \text{Ƒ}_{\delta_{\text{₳}}+1}^{q}}} - \frac{1}{2}\right)\pi_{\delta_{\text{₳}}}^{q}$$

$$S(\delta_{\text{₦}}) = \text{Ƭ}_{\delta_{\text{₦}}}^{q} - \text{Ɨ}_{\delta_{\text{₦}}}^{q} - \text{Ƒ}_{\delta_{\text{₦}}}^{q} + \left(\frac{e^{\text{Ƭ}_{\delta_{\text{₦}}}^{q} - \text{Ɨ}_{\delta_{\text{₦}}}^{q} - \text{Ƒ}_{\delta_{\text{₦}}}^{q}}}{e^{\text{Ƭ}_{\delta_{\text{₦}}}^{q} - \text{Ɨ}_{\delta_{\text{₦}}}^{q} - \text{Ƒ}_{\delta_{\text{₦}}+1}^{q}}} - \frac{1}{2}\right)\pi_{\delta_{\text{₦}}}^{q}$$

We have, $\text{Ƭ}_{\delta_{\text{₳}}} = \text{₩}_{\delta_{\text{₦}}}, \text{Ɨ}_{\delta_{\text{₳}}} = \text{₵}_{\delta_{\text{₦}}}$ and $\text{Ƒ}_{\delta_{\text{₳}}} = \text{₱}_{\delta_{\text{₦}}}$. Hence

$\Rightarrow \pi_{\delta_{\text{₳}}}^{q} = \pi_{\delta_{\text{₦}}}^{q}$

$\Rightarrow \left(\text{Ƭ}_{\delta_{\text{₳}}}, \text{Ɨ}_{\delta_{\text{₳}}}, \text{Ƒ}_{\delta_{\text{₳}}}\right) = \left(\text{₩}_{\delta_{\text{₦}}}, \text{₵}_{\delta_{\text{₦}}}, \text{₱}_{\delta_{\text{₦}}}\right)$

Proved that q-RONS$_f$WA $\left(\text{₳}_{\mathfrak{b}_{11}}, \text{₳}_{\mathfrak{b}_{12}}, \ldots, \text{₳}_{\mathfrak{b}_{nm}}\right) <$ q – RONS$_f$WA $\left(\text{₦}_{\mathfrak{b}_{11}}, \text{₦}_{\mathfrak{b}_{12}}, \ldots, \text{₦}_{\mathfrak{b}_{nm}}\right)$.

Proof (b),(c),(d): Straightforward as above.

Property 4 (Shift Invariance):

a. If $₦_{ë} = (₩_{ij}, ₵_{ij}, ₱_{ij})$ is q-RONS$_f$N$_s$, then q-RONS$_f$WA $(₳_{ϸ_{11}} \oplus ₦_ϸ, ₳_{ϸ_{12}} \oplus ₦_ϸ, \ldots, ₳_{ϸ_{nm}} \oplus ₦_ϸ)$ = q-RONS$_f$WA $(₳_{ϸ_{11}}, ₳_{ϸ_{12}}, \ldots, ₳_{ϸ_{nm}}) \oplus ₦_ϸ$.

b. If $₦_{ë} = (₩_{ij}, ₵_{ij}, ₱_{ij})$ is q-RONS$_f$N$_s$, then q-RONS$_f$OWA $(₳_{ϸ_{11}} \oplus ₦_ϸ, ₳_{ϸ_{12}} \oplus ₦_ϸ, \ldots, ₳_{ϸ_{nm}} \oplus ₦_ϸ)$ = q-RONS$_f$OWA $(₳_{ϸ_{11}}, ₳_{ϸ_{12}}, \ldots, ₳_{ϸ_{nm}}) \oplus ₦_ϸ$.

c. If $₦_{ë} = (₩_{ij}, ₵_{ij}, ₱_{ij})$ is q-RONS$_f$N$_s$, then q-RONS$_f$WG $(₳_{ϸ_{11}} \oplus ₦_ϸ, ₳_{ϸ_{12}} \oplus ₦_ϸ, \ldots, ₳_{ϸ_{nm}} \oplus ₦_ϸ)$ = q-RONS$_f$WG $(₳_{ϸ_{11}}, ₳_{ϸ_{12}}, \ldots, ₳_{ϸ_{nm}}) \oplus ₦_ϸ$.

d. If $₦_{ë} = (₩_{ij}, ₵_{ij}, ₱_{ij})$ is q-RONS$_f$N$_s$, then q-RONS$_f$OWG $(₳_{ϸ_{11}} \oplus ₦_ϸ, ₳_{ϸ_{12}} \oplus ₦_ϸ, \ldots, ₳_{ϸ_{nm}} \oplus ₦_ϸ)$ = q-RONS$_f$OWG $(₳_{ϸ_{11}}, ₳_{ϸ_{12}}, \ldots, ₳_{ϸ_{nm}}) \oplus ₦_ϸ$

Proof (a): Since $₦_ϸ = (₩, ₵, ₱)$ and $₳_{ϸ_{ij}} = (Ʈ_{ϸ_{ij}}, ƚ_{ϸ_{ij}}, Ƒ_{ϸ_{ij}})$ are the q-ROP soft numbers, so

$₳_{ϸ_{ij}} \oplus ₦_ϸ = \left(\sqrt[q]{\left(1 - Ʈ_{ij}^q\right)(1 - ₩^q)}, ƚ_{ij}^q ₵, Ƒ_{ij}^q ₱\right)$. Therefore,

q-RONS$_f$WA $(₳_{ϸ_{11}} \oplus ₦_ϸ, ₳_{ϸ_{12}} \oplus ₦_ϸ, \ldots, ₳_{ϸ_{nm}} \oplus ₦_ϸ) = \oplus_{j=1}^m \upsilon_j \left(\oplus_{i=1}^n \omega_i \left(₳_{ϸ_{ij}} \oplus ₦_ϸ\right)\right)$

$= \left(\sqrt[q]{1 - \Pi_{j=1}^m \left(\Pi_{i=1}^n \left(1 - Ʈ_{ij}^q\right)^{\omega_i} (1 - ₩^q)^{\omega_i}\right)^{\upsilon_j}}, \Pi_{j=1}^m \left(\Pi_{i=1}^n ƚ_{ij}^{\omega_i} ₵^{\omega_i}\right)^{\upsilon_j}, \Pi_{j=1}^m \left(\Pi_{i=1}^n Ƒ_{ij}^{\omega_i} ₱^{\omega_i}\right)^{\upsilon_j}\right)$

$= \left(\sqrt[q]{1 - (1 - ₩^q)\Pi_{j=1}^m \left(\Pi_{i=1}^n \left(1 - Ʈ_{ij}^q\right)^{\omega_i}\right)^{\upsilon_j}}, ₵\Pi_{j=1}^m \left(\Pi_{i=1}^n ƚ_{ij}^{\omega_i}\right)^{\upsilon_j}, ₱\Pi_{j=1}^m \left(\Pi_{i=1}^n Ƒ_{ij}^{\omega_i}\right)^{\upsilon_j}\right)$

$= \left(\sqrt[q]{1 - \Pi_{j=1}^m \left(\Pi_{i=1}^n \left(1 - Ʈ_{ij}^q\right)^{\omega_i}\right)^{\upsilon_j}}, \Pi_{j=1}^m \left(\Pi_{i=1}^n ƚ_{ij}^{\omega_i}\right)^{\upsilon_j}, \Pi_{j=1}^m \left(\Pi_{i=1}^n Ƒ_{ij}^{\omega_i}\right)^{\upsilon_j}\right) \oplus (₩, ₵, ₱)$

q-RONS$_f$WA $(₳_{ϸ_{11}}, ₳_{ϸ_{12}}, \ldots, ₳_{ϸ_{nm}}) \oplus ₦_ϸ$.

Proof (b),(c),(d): Straightforward as above.

Property 5 (Homogeneity):

a. If λ ≥ 0, then q-RONS$_f$WA $(λ₳_{ϸ_{11}}, λ₳_{ϸ_{12}}, \ldots, λ₳_{ϸ_{nm}})$ = λ q-RONS$_f$WA $(₳_{ϸ_{11}}, ₳_{ϸ_{12}}, \ldots, ₳_{ϸ_{nm}})$.
b. If λ ≥ 0, then q-RONS$_f$OWA $(λ₳_{ϸ_{11}}, λ₳_{ϸ_{12}}, \ldots, λ₳_{ϸ_{nm}})$ = λ q-RONS$_f$OWA $(₳_{ϸ_{11}}, ₳_{ϸ_{12}}, \ldots, ₳_{ϸ_{nm}})$.
c. If λ ≥ 0, then q-RONS$_f$WG $(λ₳_{ϸ_{11}}, λ₳_{ϸ_{12}}, \ldots, λ₳_{ϸ_{nm}})$ = λ q-RONS$_f$WG $(₳_{ϸ_{11}}, ₳_{ϸ_{12}}, \ldots, ₳_{ϸ_{nm}})$.
d. If λ ≥ 0, then q-RONS$_f$OWG $(λ₳_{ϸ_{11}}, λ₳_{ϸ_{12}}, \ldots, λ₳_{ϸ_{nm}})$ = λ q-RONS$_f$OWG $(₳_{ϸ_{11}}, ₳_{ϸ_{12}}, \ldots, ₳_{ϸ_{nm}})$.

Proof (a): Assume that λ ≥ 0 and let $₳_{ϸ_{ij}} = (Ʈ_{ϸ_{ij}}, ƚ_{ϸ_{ij}}, Ƒ_{ϸ_{ij}})$ be a q-RONS$_f$N$_s$, then

$λ₳ = \left(\sqrt[q]{1 - \left(1 - Ʈ_{ij}^q\right)^{λ}}, ƚ_{ij}^q, Ƒ_{ij}^q\right)$

$$q - RONS_f WA\left(λ₳_{ϸ_{11}}, λ₳_{ϸ_{12}}, \ldots, λ₳_{ϸ_{nm}}\right) = \begin{pmatrix} \sqrt[q]{1 - \left(\prod_{j=1}^{m}\left(\prod_{i=1}^{n}\left(1 - Ʈ_{ij}^q\right)^{\omega_i}\right)^{\upsilon_j}\right)^{λ}}, \\ \left(\Pi_{j=1}^m \left(\Pi_{i=1}^n ƚ_{ij}^{\omega_i}\right)^{\upsilon_j}\right)^{λ}, \\ \left(\Pi_{j=1}^m \left(\Pi_{i=1}^n Ƒ_{ij}^{\omega_i}\right)^{\upsilon_j}\right)^{λ} \end{pmatrix}$$

$= λ\, q - RONS_f WA\left(₳_{ϸ_{11}}, ₳_{ϸ_{12}}, \ldots, ₳_{ϸ_{nm}}\right)$.

Proof (b),(c),(d): Straightforward as above.

4. Mathematical model based on q-RON soft information for MCGDM:

In this section, we consider a mathematical model based on the proposed structure for solving the MCDM problem. DM is a pre-planned procedure for identifying and selecting the optimal option from a variety of alternatives.

For this let the set of alternatives represented by A= {a_1, a_2, ……a_l} and set of parameters $\hat{C}$= {$\hat{c}_1$, $\hat{c}_2$……, $\hat{c}_m$}. After evaluating each alternative to their corresponding parameters, expert give their assessment in the form of from $A_{\ddot{e}_{ij}}$=(T_{ij}, I_{ij}, F_{ij}) with WV ω= $(\omega_1, \omega 2, \ldots, \omega n)^T$ for expert x_i having $\sum_{i=1}^{n} \omega_i = 1$ and for parameters $\sum_{i=1}^{n} \upsilon_i = 1$.

Where M=$\left[A_{b_{ij}}\right]_{m\times n}$ expressed the collected information from professional experts and then applied a developed model on the decision matrix to get an aggregated $q-RONS_fN_s$ for each alternative against their parameters. Finally, we compute the score value of aggregated data and rank the alternatives for optimum results. The overall framework and sequential procedure for the proposed methodology are outlined as follows: Figure 3 represent the flow chat of proposed model.

Algorithm: Using $\mathbf{q-RONS_fWA}$ and $\mathbf{q-RONS_fWG}$, to deal MADM problem.

Input:

Step 1. Established a decision matrix M=$\left[A_{b_{ij}}\right]_{\mathbf{m\times n}}$.

Step 2. Normalize decision matrix M=$\left[A_{b_{ij}}\right]_{\mathbf{m\times n}}$

$$\mathbf{P}_{ij} = \begin{cases} A_{b_{ij}}^{C}: \textbf{for cost type parameter} \\ A_{b_{ij}} \textbf{for benefit type parameter} \end{cases}$$

where $A_{b_{ij}}^{C}$ represent the complement of $A_{b_{ij}}$.

Step 3. To evaluate the decision matrix M=$\left[A_{b_{ij}}\right]_{\mathbf{m\times n}}$ using q-RON$\mathbf{S_f}$WA and q-RON$\mathbf{S_f}$WG operators.

Step 4. To aggregate data compute score values.

Step 5. For optimum results rank the alternatives using score values.

Output:

The choice will be determined by the alternative that achieves the highest score.

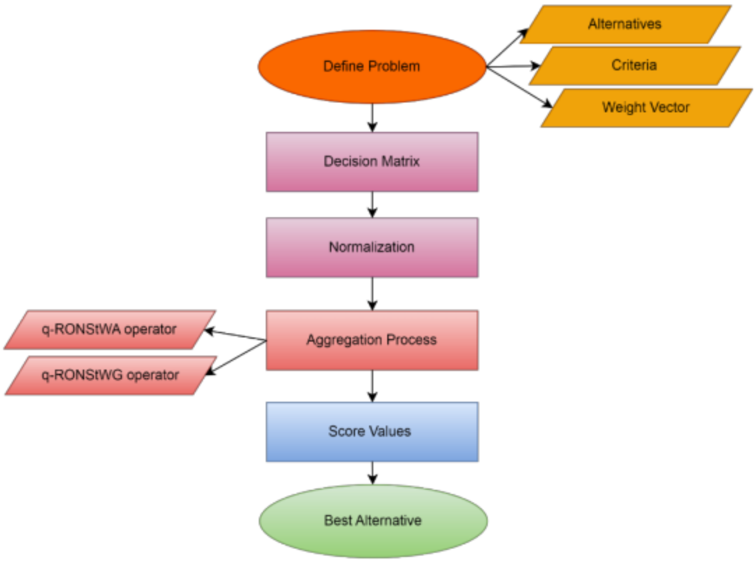

Figure 3. Flow chart of the proposed model problem through aggregation operators.

4.1. Decision making problem for the selection of employees of a particular position in a real estate company:

In real-life real estate industry play an important role for the development of country by encompasses a wide-range of activities related to buying, selling, leasing and development of properties such as agriculture, residential, commercial and industrial land. Real estate industry affected by various thing such as technology, laws and economy of a country. Also, it plays an important role in urban development, job creation, wealth generation and economic growth. Here we consider aa example of a typical decision-making challenge in this field. That company not only value the technical skill of the candidates (employee) for a particular position in a real estate company but also their soft skills like commination, adaptability and leadership, which are crucial for managing team and dealing with clients effectively. For this scenario, we utilized our proposed model of q-RONSS which characterized the problem by tripled memberships with attributes and qualification required for the job including both aspect of qualitative and quantitative. To assess their suitability for the particular position in a company, each candidate must be evaluated their profile using the proposed model.

- Representation of candidate's profile: The qualification, experience related to the field, communication skills and general aptitude of each candidate are representing by utilizing the proposed model ($q - RONS_f S$).
- Assessment of suitability: Utilizing q-rung orthopair neutrosophic soft operators, the attributes of each candidate can be combining and compare by the company with the requirement of particular position.
- Decision-making: Based on $q - RONS_f$ evaluation, the company can make informed decision related to candidate selection.

Adaptability of changing requirement: To ensuring the continued effectiveness of the selection process of employ, the company need to updated and improve the proposed model according to feedback and evolving business requirement.

4.2 ***Application of proposed model through numerical example***

In this section, we consider a numerical example related to the personal selection problem of employees for a particular position in a real estate company namely "Makaan Solution". But this selection not only value the technical skill of the employee but also their soft skills like commination, adaptability and leadership, which are crucial for managing team and dealing with clients effectively. For this scenario, we utilized our proposed model of q-RONSS which characterized the problem by tripled memberships with attributes and qualification required for the job including both aspect of qualitative and quantitative. After the preliminary elimination process, four candidates $\{\times_1, \times_2, \times_3, \times_4\}$ which express the alternatives, are entering the final round of interviews. Which are evaluated by a team of four expert decision-makers:

Group of experts:

DM_1 = Rizwan chema,

DM_2 = Sana ch.,

DM_3 = Nimra ch.,

DM_4 = Salman chema

having weight vector ω = $(0.20,0.25,0.27,0.28)^T$. To asses their suitability for the particular position in a company, each candidate must be evaluated their profile using the proposed model on the bases on criteria

Criteria:

$\mathfrak{y}_1$ = Qualification: The expert evaluates the education background of candidate, that he possesses the necessary academic qualification required for the position in the real estate company.

$\mathfrak{y}_2$ = Experience related to the field: Candidate must have a strong track record of success and demonstrated expertise in the real estate sector.

$\mathfrak{y}_3$ = Communication skills: Strong communication skills are the main factor of real estate sector. So the candidate must have the ability to convey information clearly, listen actively, negotiate effectively, and built rapport with other.

$\mathfrak{y}_4$ = General aptitude: Candidate with a well-rounded skill, decision-making capabilities, adaptability of new situations and positive attitude are often considered suitable for various role in a real estate company.

having weight vector υ = $(0.5,0.16,0.14,0.20)^T$. For the selection of the best employee for the company, a step-wise algorithm is constructed. Figure 4 express a decision tree for the personal selection problem of employees.

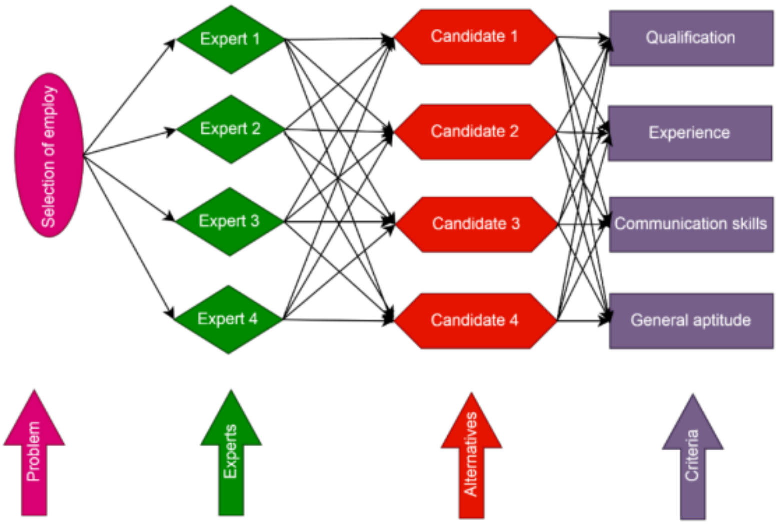

Figure 4. Decision tree for site selection of best employ for the company.

By using q-RONSWAO:

Step 1: Established M = $\left[A_{b_{ij}}\right]_{m\times n}$, shown in Tables 4 to 7, respectively.

Table 4. Rating values for alternative $\times_1$ provided by a decision maker.

Experts	$\mathfrak{y}_1$	$\mathfrak{y}_2$	$\mathfrak{y}_3$	$\mathfrak{y}_4$
$\mathbf{DM_1}$	(.8, .3, .1)	(.9, .1, .2)	(.8, .6, .3)	(.7, .4, .2)
$\mathbf{DM_2}$	(0.7,0.4,0.2)	(0.6,0.3,0.3)	(0.7,0.4,0.3)	(0.8,0.2,0.4)
$\mathbf{DM_3}$	(0.5,0.4,0.2)	(0.2,0.1,0.3)	(0.3,0.4,0.3)	(0.5,0.3,0.7)
$\mathbf{DM_4}$	(0.4,0.1,0.3)	(0.3,0.6,0.2)	(0.4,0.1,0.2)	(0.1,0.1,0.1)

Table 5. Rating values for alternative $\times_2$ provided by a decision maker

Experts	$\mathfrak{y}_1$	$\mathfrak{y}_2$	$\mathfrak{y}_3$	$\mathfrak{y}_4$
$\mathbf{DM_1}$	(.8, .4, .2)	(.9, .1, .5)	(.8, .4, .3)	(.1, .3, .2)
$\mathbf{DM_2}$	(0.2,0.1,0.2)	(0.5,0.3,0.3)	(0.5,0.4,0.3)	(0.8,0.2,0.2)
$\mathbf{DM_3}$	(0.3,0.2,0.3)	(0.2,0.5,0.1)	(0.3,0.3,0.3)	(0.2,0.1,0.1)
$\mathbf{DM_4}$	(0.4,0.6,0.1)	(0.3,0.1,0.2)	(0.4,0.1,0.2)	(0.7,0.3,0.4)

Table 6. Rating values for alternative $\times_3$ provided by a decision maker.

Experts	$\mathfrak{y}_1$	$\mathfrak{y}_2$	$\mathfrak{y}_3$	$\mathfrak{y}_4$
$\mathbf{DM_1}$	(.8, .3, .1)	(.9, .1, .5)	(.8, .6, .3)	(.7, .4, .2)
$\mathbf{DM_2}$	(0.4,0.1,0.3)	(0.7,0.3,0.1)	(0.2,0.1,0.3)	(0.4,0.1,0.4)
$\mathbf{DM_3}$	(0.7,0.4,0.2)	(0.6,0.1,0.3)	(0.7,0.5,0.1)	(0.6,0.3,0.1)
$\mathbf{DM_4}$	(0.3,0.2,0.3)	(0.8,0.6,0.2)	(0.4,0.3,01)	(0.8,0.2,0.4)

Table 7. Rating values for alternative $\times_4$ provided by a decision maker

Experts	$\mathfrak{y}_1$	$\mathfrak{y}_2$	$\mathfrak{y}_3$	$\mathfrak{y}_4$
$\mathbf{DM_1}$	(.4, .3, .1)	(.5, .1, .2)	(.1, .1, .3)	(.2, .1, .2)
$\mathbf{DM_2}$	(0.3,0.2,0.4)	(0.4,0.3,0.3)	(0.5,0.4,0.3)	(0.1,0.2,0.4)
$\mathbf{DM_3}$	(0.5,0.5,0.5)	(0.3,0.2,0.3)	(0.3,0.2,0.1)	(0.5,0.3,0.7)
$\mathbf{DM_4}$	(0.1,0.1,0.4)	(0.1,0.1,0.1)	(0.4,0.1,0.2)	(0.4,0.2,0.4)

Step 2: All the parameters are the same, so no need for normalization.

Step 3: Using the $q-RONS_fWA$ operator, to evaluate each value of alternative, so we get

$\vartheta_1 = (0.6394, 0.2447, 0.2268)$

$\vartheta_2 = (0.5845, 0.2376, 0.2031)$

$\vartheta_3 = (0.6681, 0.2284, 0.2135)$

$\vartheta_4 = (0.3790, 0.2004, 0.2928)$

Step 4. To compare each alternative, determine the score value.

$S(\vartheta_1) = 0.2768$

$S(\vartheta_2) = 0.2124$

$S(\vartheta_3) = 0.3233$

$S(\vartheta_4) = 0.0261$

Step 5. To get the best choice, rank the score values.

$0.3233 > 0.2768 > 0.2124 > 0.026$

$S(\vartheta_3) > S(\vartheta_1) > S(\vartheta_2) > S(\vartheta_4)$

The above ranking shows that, ϑ_3 is the best employ for a particular position in a real estate company, using $\mathrm{q} - \mathrm{RONS_fWAO}$.

By using q-RPNSWG operator:

Step 1: Similar as above .

Step 2: Similar as above.

Step 3: Using the $\mathrm{q} - \mathrm{RONS_fWG}$ operator, to evaluate each value of alternative, so we get

$\vartheta_1 = (0.4829, 0.3615, 0.3393)$

$\vartheta_2 = (0.3712, 0.3816, 0.2659)$

$\vartheta_3 = (0.5404, 0.3468, 0.2853)$

$\vartheta_4 = (0.2697, 0.3040, 0.4080)$

Step 4. To compare each alternative, determine the score value.

$S(\vartheta_1) = 0.0316$

$S(\vartheta_2) = -0.0283$

$S(\vartheta_3) = 0.1109$

$S(\vartheta_4) = -0.0951$

Step 5. To get the best choice, rank the score values.

$0.1109 > 0.0316 > -0.0283 > -0.0951$

$S(\vartheta_3) > S(\vartheta_1) > S(\vartheta_2) > S(\vartheta_4)$

The above ranking shows that, $\vartheta_3$is the best employ for a particular position in a real estate company, using $\mathrm{q} - \mathrm{RONS_fWG}$ operator.

Table 8. Show the Ranking Order using $\mathrm{q} - \mathrm{RONS_fWA}$ and $\mathrm{q} - \mathrm{RONS_fWG}$ operators.

Operators	Score values				Ranking
	ϑ_1	ϑ_2	ϑ_3	ϑ_4	
$\mathbf{q - RONS_fWA}$	0.2768	0.2124	0.3233	0.0261	$S(\vartheta_3) > S(\vartheta_1) > S(\vartheta_2) > S(\vartheta_4)$
$\mathbf{q - RONS_fWG}$	0.0316	-0.0283	0.1109	-0.0951	$S(\vartheta_3) > S(\vartheta_1) > S(\vartheta_2) > S(\vartheta_4)$

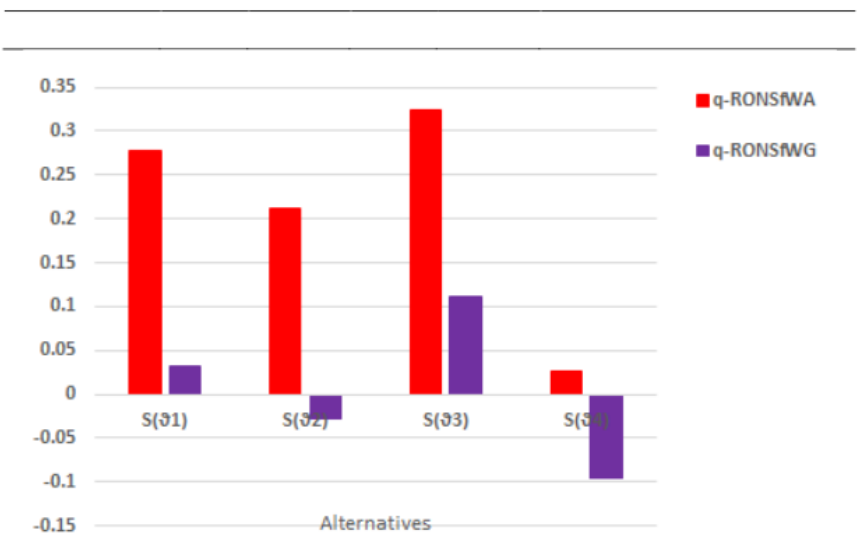

Figure 5. Graphical representation of score values $q-RONS_fWA$ and $q-RONS_fWG$.

5. Managerial implications:

The authenticity and superiority of proposed model two pivotal analyses are conducted, comparison analysis and characteristic analysis with existing approaches, express in this section.

5.1. Comparative Analysis:

To utilize a variety of proposed operators, we perform comparative analysis of proposed model with existing approaches see ([24], [25], [26], [27]). In this section of main goal to show the authenticity of our proposed model, for this we compare the ranking ordered of alternative of suggested model with existing. The ranking order and similarity of best alternative (ϑ_3), emphasizes the exceptional flexibility and comprehensiveness of our proposed technique. This flexibility is particularly given in Table 9 and the comparative analysis is express in Figure.

Table. 9: Show comparative analysis with existing operators.

Aggregation Operators	Score values				Ranking	Best Alternative
	ϑ_1	ϑ_2	ϑ_3	ϑ_4		
$q-ROFS_fYWA$ [24]	0.44	0.41	0.49	0.45	$\vartheta_3 > \vartheta_4 > \vartheta_1 > \vartheta_2$	ϑ_3
$q-ROFS_fYWG$ [24]	0.26	0.25	0.41	0.39	$\vartheta_3 > \vartheta_4 > \vartheta_1 > \vartheta_2$	ϑ_3
P_yFS_fWG [25]	−0.0911	−0.0132	0.0505	0.0159	$\vartheta_3 > \vartheta_4 > \vartheta_2 > \vartheta_1$	ϑ_3
$q-ROFS_fWA$ [26]	0.4149	0.4654	0.5224	0.4849	$\vartheta_3 > \vartheta_4 > \vartheta_1 > \vartheta_2$	ϑ_3
$q-ROFS_fWG$ [27]	0.3493	0.4048	0.4648	0.4337	$\vartheta_3 > \vartheta_4 > \vartheta_1 > \vartheta_2$	ϑ_3
$q-RONS_fWA$ (Proposed)	0.2768	0.2124	0.3233	0.0261	$\vartheta_3 > \vartheta_1 > \vartheta_2 > \vartheta_4$	ϑ_3
$q-RONS_fWG$ (Proposed)	0.0316	−0.0283	0.1109	−0.0951	$\vartheta_3 > \vartheta_1 > \vartheta_2 > \vartheta_4$	ϑ_3

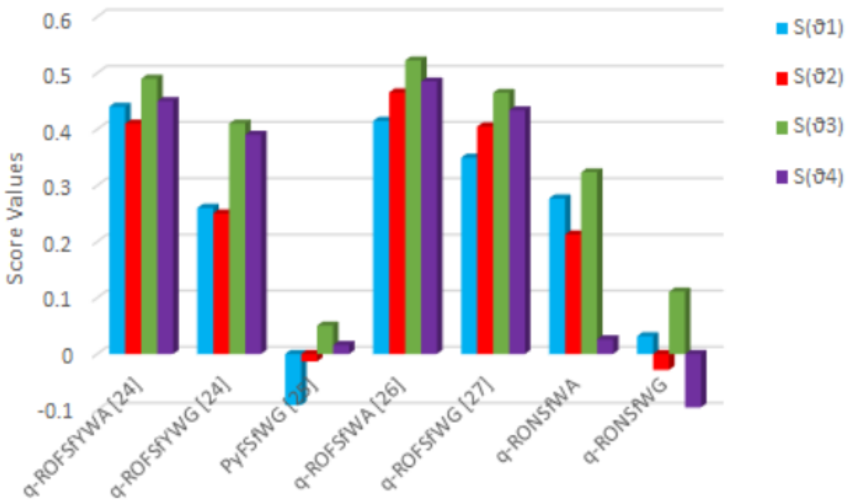

Figure 6. Graphical representation of comparison analysis.

5.2. Characteristic Analysis:

Through characteristic analysis with various existing theories see ([1], [20], [6], [14], [18], [19]) as summarized in Table 10, we explore the superiority of proposed structure. After analysis the existing theories, we conclude that existing approaches deal the uncertainties in MADM problem with MF and non-MF associated with attributes, while it does not address the degree of indeterminacy and also it is important to admit that the existing theories has its specific limitations, which restricts decision-maker to evaluating alternatives within the domain. So, to tackle these limitations we proposed more flexible and free structure by using NS in q-ROFSS and proposed novel structure of q-RONSS, characterized by three parameter, truth, indeterminacy and false membership with attributes provides the more flexible environment to the decision maker by relaxing the domain. This is the logic behind our proposed approach that neutrosophic set handles the situation $0 \leq \mu + \eta + \nu \leq 3$, but failed the condition $(0 \leq (\mu)^q + (\eta)^q + (\nu)^q \leq 3)$. Figure 7 Show the characteristic analysis of proposed model with existing theories.

Table10. Characteristic analysis of various theories.

Theories	Truth Membership	Indeterminacy membership	False membership	Parameter	Domain
FS [**1**]	✓	×	×	×	$0 \leq \mathrm{a} \leq 1$
IFS [**20**]	✓	×	✓	×	$0 \leq \mathrm{a} + \mathrm{b} \leq 1$
PyFS [**6**]	✓	×	✓	×	$0 \leq a^2 + b^2 \leq 1$
q-ROFS [**14**]	✓	×	✓	×	$0 \leq a^q + b^q \leq 1\, (q \geq 1)$
q-ROF$\mathbf{S_tS}$ [**18**]	✓	×	✓	✓	$0 \leq a^q + b^q \leq 1\, (q \geq 1)$
NS [**19**]	✓	✓	✓	×	$0 \leq \mathrm{a} + \mathrm{b} + \mathrm{c} \leq 3$
[This Paper]	✓	✓	✓	✓	$0 \leq a^q + b^q + c^q \leq 3\, (q \geq 1)\,)$

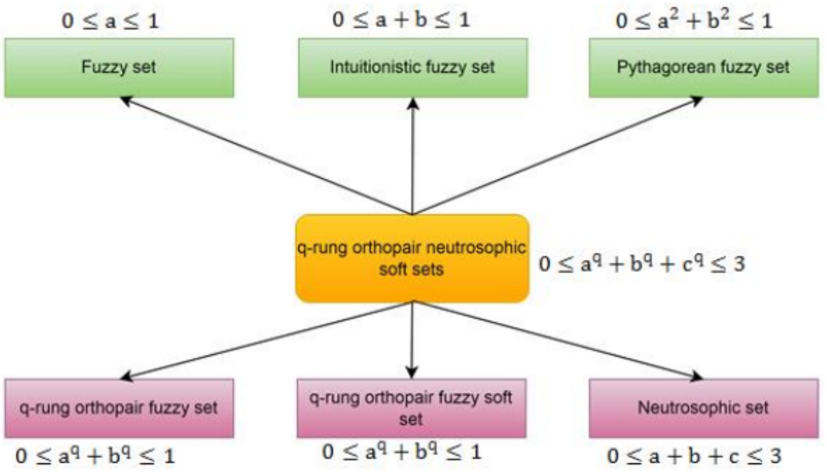

Figure 7. Show the characteristic analysis of proposed model with existing theories.

5.3. Sensitivity Analysis:

In this segment, we address the reliability and influence of various values of parameters "q" impact the alternatives and the ultimate ranking outcomes by using aggregation operators. Our main goal investigate the effect of "q" on the score of alternatives. To understand the relation between parameters variation and decision-outcome, we utilize $q-\mathrm{RONFS_tWA}$ operator on the various values of q = 3, 4 ,.....,10 as shown in Table 11. If we increase the value of parameter q and the ranking order of alternative remain consistent $\vartheta_3 > \vartheta_1 > \vartheta_2 > \vartheta^4$ and the score values of alternatives increase, which show a strong consistency in decision outcomes. The behaver of alternatives for different values of "q" by using $q-\mathrm{RONFS_tWA}$ operator is presented in Figure 8. If we utilize $q-\mathrm{RONFS_t}$ WG operator by increase the value of parameter q = 3, 4 ,...,10 , the ranking order of alternative $\vartheta_3 > \vartheta_1 > \vartheta_2 > \vartheta^4$ and best alternative ϑ_3 remain consistent and the score values of alternatives decrease, which show a strong consistency in decision outcomes. The behaver of alternatives for different values of "q" by using $q-\mathrm{RONFS_tWG}$ operator is presented in Figure 9.

Which show that the attitudes of decision maker can be reflected by the various values of parameter. When the value of parameter lower decision-maker get more optimistic approach and if higher the value, more doubtful the decision-maker. So, decision-maker have the flexibility to select a suitable value of parameter according to their preferences.

Table 11. Sorting alternatives according to their respective parameter q values using $q-\mathrm{RONFS_tWA}$ and $q-\mathrm{RONFS_tWG}$ operator.

Parameter $\boldsymbol{q}$	Ranking order	Best alternative
$\boldsymbol{q}=\mathbf{3}$	$\vartheta_3 > \vartheta_1 > \vartheta_2 > \vartheta_4$	ϑ_3
$\boldsymbol{q}=\mathbf{4}$	$\vartheta_3 > \vartheta_1 > \vartheta_2 > \vartheta_4$	ϑ_3
$\boldsymbol{q}=\mathbf{5}$	$\vartheta_3 > \vartheta_1 > \vartheta_2 > \vartheta_4$	ϑ_3
$\boldsymbol{q}=\mathbf{6}$	$\vartheta_3 > \vartheta_1 > \vartheta_2 > \vartheta_4$	ϑ_3
$\boldsymbol{q}=\mathbf{7}$	$\vartheta_3 > \vartheta_1 > \vartheta_2 > \vartheta_4$	ϑ_3
$\boldsymbol{q}=\mathbf{8}$	$\vartheta_3 > \vartheta_1 > \vartheta_2 > \vartheta_4$	ϑ_3
$\boldsymbol{q}=\mathbf{9}$	$\vartheta_3 > \vartheta_1 > \vartheta_2 > \vartheta_4$	ϑ_3
$\boldsymbol{q}=\mathbf{10}$	$\vartheta_3 > \vartheta_1 > \vartheta_2 > \vartheta_4$	ϑ_3

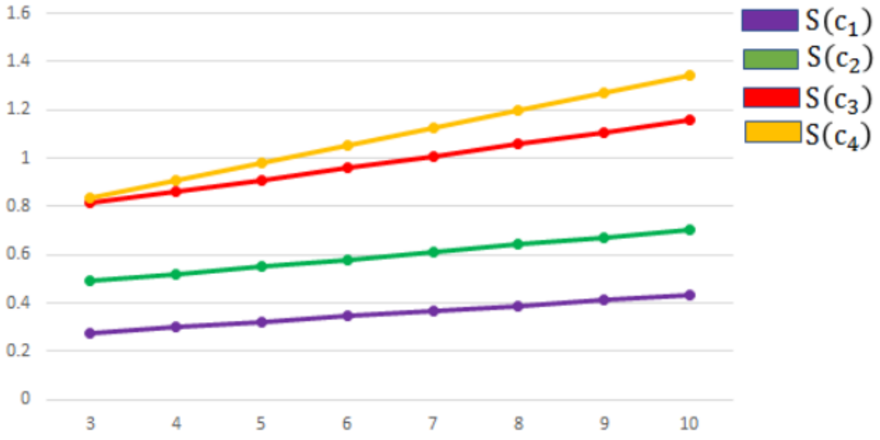

Figure 8. Ranking order of various values of parameter r and q by using $q-\mathrm{RONFS_tWA}$.

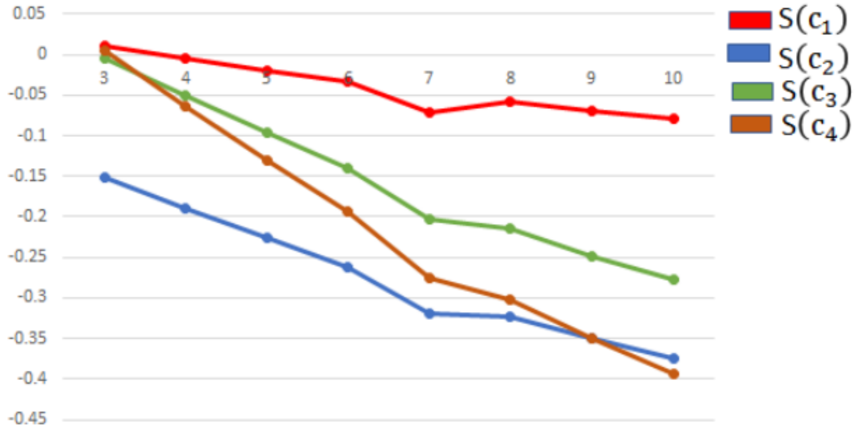

Figure 9. Ranking order of various values of parameter r and q by using $q-\mathrm{RONFS_tWG}$.

5.4. Advantages:

The proposed technique has different benefits:

1. Our proposed model effectively overcome the challenges posed by the existing approaches have also the ability to demonstrating high level of proficiency and provide robust framework to manage uncertainty across diverse scenarios.
2. Our proposed model having a unique characteristic to address the limitation of indeterminacy degree and provides the more flexible environment to the decision maker by relaxing the domain.
3. The suggested approach is proficient in addressing MCDM problems, particularly in the context of more realistic and complex scenarios.

6. Conclusion and future recommendation

The main goal of this article is to develop a flexible model, to cover the limitation of indeterminacy. Because in the existing approaches, we deal the uncertainties in MADM problem with MD and NMD associated with attributes, while it does not address the degree of indeterminacy and also it is important to admit that the existing theories has its specific limitations, which restricts decision-maker to evaluating alternatives within the domain. So, to tackle these limitations we proposed more flexible and free structure by merging NS with q-ROFSS and proposed more q-

RONSS, characterized by three parameter, truth, indeterminacy and false membership with attributes provides the more flexible environment to the decision maker by relaxing the domain. This manuscript's primary achievement is to originate q-rung orthopair neutrosophic soft set and their basic operational laws. We also investigate some aggregation operators like q-RONSWA, q-RONSOWA, q-RONSWG and q-RONSOWG operators as well as a demonstration of their fundamental properties. Moreover, on the base of the proposed structure, we build a step-wise algorithm and mathematical model for solving MADM problem. At the end, for application, we consider a numerical example related to the personal selection problem of employ for a particular position in a real estate company and finally, show the superiority and authenticity of the proposed model using various analysis test analysis with the existing model.

A proposed structure has certain limitation, related to the personal selection problem of employees for a particular position in a real estate company on the bases of criteria. In this particular scenario, the scope of validation may be limited. It's important to assess the wide range of real-world decision challenge, to validate the robustness of $q - RONS_fS_s$ framework.

In future, the proposed model could find application in various direction such as TOPSIS Method, AHP Method, CODAS Method, quasirung orthopair fuzzy set [43], 3,4-quasirung fuzzy sets [44] and different AOs such as Complex q-Rung Orthopair Fuzzy Aczel–Alsina power aggregation operators [62], complex q-rung picture fuzzy Aczel–Alsina prioritized ordered operators [63] , also in various fields, including business, engineering sectors, medical sector and different method for the selection of various projects.

Funding

This research did not receive any external funding.

Consent for publication

This manuscript has not been published and is not under consideration for publication elsewhere.

Data availability statement

The accompanying manuscript does not contain any associated data. The paper only presents the written text and does not have any additional data that supports the claims and conclusions presented in the manuscript.

CRediT authorship contribution statement

Sumbal Ali: Conceptualization. **Asad Ali**: Supervision**. Ahmad Bin Azim**: Software**. Ahmad Aloqaily**: Investigation. Nabil Mlaiki: Data curation.

Conflict of Interests

The authors confirm that they do not possess any discernible conflicting financial interests or personal relationships that could appear to impact the research detailed in this paper.

Acknowledgments:

The authors A. ALoqaily and N. Mlaiki would like to thank Prince Sultan University for paying the APC and for the support through the TAS research lab.

References

1. Zadeh, L.A., *Fuzzy sets.* Information and control, 1965. **8**(3): p. 338-353.
2. Zadeh, L.A., *The concept of a linguistic variable and its application to approximate reasoning—I.* Information sciences, 1975. **8**(3): p. 199-249.
3. H. Zhao, Z. Xu, M. Ni, S. Liu, Generalized aggregation operators for intuitionistic fuzzy sets, Int. J. Intell. Syst., **25** (2010), 1-30.
4. C. Tan, Generalized intuitionistic fuzzy geometric aggregation operator and its application to multi-criteria group decision-making, Soft Comput., **15** (2011), 867-876.
5. C. Tan, W. Yi, X. Chen, Generalized intuitionistic fuzzy geometric aggregation operators and their application to multi-criteria decision making, J. Oper. Res. Soc., 66 (2015), 1919-19.

6. R.R. Yager, Pythagorean fuzzy subsets, in 2013 Joint IFSA World Congress and NAFIPS Annual Meeting (IFSA/NAFIPS), IEEE, Edmonton, Canada, (2013),57-61.
7. Yager RR. Pythagorean membership grades in multi-criteria decision making, IEEE Trans Fuzzy Syst., **22** (2014), 958-965.
8. Yager RR, Abbasov AM. Pythagorean membership grades, complex numbers, and decision making, Int J Intell Syst., **2** (2014), 436-452.
9. P. Liu, P. Wang, Some q-rung orthopair fuzzy aggregation operators and their applications to multiple-attribute decision-making, Int. J. Intell. Syst., **33** (2018), 259-280.
10. P. Liu, J. Liu, some q-rung orthopair fuzzy Bonferroni mean operators and their application to multi-attribute group decision-making, Int. J. Intell. Syst., **33** (2018), 315-347.
11. P. Liu, S.M. Chen, P. Wang, Multiple-attribute group decision-making based on q-rung orthopair fuzzy power maclurin symmetric mean operators, IEEE Trans. Syst. Man Cybern. Syst., (2018), 1-16.
12. C. Jana, G. Muhiuddin, M. Pal, Some Dombi aggregation of q-rung orthopair fuzzy numbers in multiple-attribute decision-making, Int. J. Intell. Syst., **34** (2019), 3220-3240.
13. H. Garg, S.M. Chen, Multi-attribute group decision-making based on neutrality aggregation operators of q-rung orthopair fuzzy sets, Inf. Sci., **517** (2020), 427-447.
14. R.R. Yager, Generalized orthopair fuzzy sets, IEEE Trans. Fuzzy Syst., **25** (2016), 1222-1230.
15. Molodtsov D. Soft set theory-Örst results, Comput Math Appl., **37** (1999), 19-31
16. PK Maji, R Biswas, AR Roy, Fuzzy soft sets, J Fuzzy Math., **9** (2001), 589-602.
17. PK Maji, R Biswas, AR Roy, Intuitionistic fuzzy soft sets, J Fuzzy Math., **9** (2001), 677-692.
18. A. Hussain, M. I. Ali, T. Mahmood & M. Munir, q-Rung orthopair fuzzy soft average aggregation operators and their application in multicriteria decision-making, International Journal of Intelligent Systems, **35**(2020), 571-599.
19. F Smarandache, A unifying field in logics neutrosophy: neutrosophic probability, set and logic, American Research Press, Rehoboth, (1999).
20. Atanassov, K.T. and S. Stoeva, *Intuitionistic fuzzy sets.* Fuzzy sets and Systems, 1986. **20**(1): p. 87-96.
21. I. Deli, & N. Çağman, Intuitionistic fuzzy parameterized soft set theory and its decision making, Applied Soft Computing, **28** (2015), 109-113.
22. I. Deli, A TOPSIS method by using generalized trapezoidal hesitant fuzzy numbers and application to a robot selection problem, Journal of intelligent & fuzzy systems, **38** (2020), 779-793.
23. I. Deli, & S. Broumi, Neutrosophic soft matrices and NSM-decision making, Journal of Intelligent & Fuzzy Systems, **28** (2015), 2233-2241.
24. Akram, M., Shahzadi, G., Butt, M. A., & Karaaslan, F. (2021). A hybrid decision making method based on q-rung orthopair fuzzy soft information. Journal of Intelligent & Fuzzy Systems, 40(5), 9815-9830.
25. Zulqarnain, R. M., Xin, X. L., Garg, H., & Khan, W. A. (2021). Aggregation operators of pythagorean fuzzy soft sets with their application for green supplier chain management. Journal of Intelligent & Fuzzy Systems, *40*(3), 5545-5563.
26. Hussain, A., Ali, M. I., Mahmood, T., & Munir, M. (2020). q-Rung orthopair fuzzy soft average aggregation operators and their application in multicriteria decision-making. International Journal of Intelligent Systems, *35*(4), 571-599.
27. Chinram, R., Hussain, A., Ali, M. I., & Mahmood, T. (2021). Some geometric aggregation operators under q-rung orthopair fuzzy soft information with their applications in multi-criteria decision making. IEEE Access, *9*, 31975-31993.
28. Azmi, F.M. Exploring Fuzzy Triple Controlled Metric Spaces: Applications in Integral Equations. Symmetry 2023, 15, 1943. https://doi.org/10.3390/sym15101943.
29. Himanshu Dhumras, Prashant Kumar Shukla, Rakesh K Bajaj, Wadii Boulila, Varun Shukla, Piyush Kumar Shukla, Vinodh Kumar Minchula and Sajjad Hussain Chauhdary, Industry 5.0 Enablers in Consumer Electronics Market Assessment Under T-Spherical Fuzzy Integrated Decision-Making Approach," in IEEE Transactions on Consumer Electronics, doi: 10.1109/TCE.2023.3325433.
30. Awais Younus, Muhammad Asif, Usama Atta, Tehmina Bashir and Thabet Abdeljawad, Applications of fuzzy conformable Laplace transforms for solving fuzzy conformable differential equations. Soft Comput 27, 8583–8597 (2023). https://doi.org/10.1007/s00500-023-08181-1.
31. Zhang, W. R. (1994, December). Bipolar fuzzy sets and relations: a computational framework for cognitive modeling and multiagent decision analysis. In *NAFIPS/IFIS/NASA'94. Proceedings of the First International Joint Conference of The North American Fuzzy Information Processing Society Biannual Conference. The Industrial Fuzzy Control and Intellige* (pp. 305-309). IEEE.

32. Zhang, W. R. (1998, May). (Yin)(Yang) bipolar fuzzy sets. In *1998 IEEE international conference on fuzzy systems proceedings. IEEE world congress on computational intelligence (Cat. No. 98CH36228)* (Vol. 1, pp. 835-840). IEEE.
33. Paul, T. K., Pal, M., & Jana, C. (2022). Portfolio selection as a multicriteria group decision making in Pythagorean fuzzy environment with GRA and FAHP framework. *International Journal of Intelligent Systems*, *37*(1), 478-515.
34. Paul, T. K., Jana, C., Pal, M., & Simic, V. (2023). Sustainable carbon-dioxide storage assessment in geological media using modified Pythagorean fuzzy VIKOR and DEMATEL approach. *International Journal of Hydrogen Energy*, *48*(25), 9474-9497.
35. Jin, L., Mesiar, R., Senapati, T., Jana, C., Ma, C., García-Zamora, D., & Yager, R. R. (2024). Ordered weighted geometric averaging operators for basic uncertain information. *Information Sciences*, 120275.
36. Jana, C., Dobrodolac, M., Simic, V., Pal, M., Sarkar, B., & Stević, Ž. (2023). Evaluation of sustainable strategies for urban parcel delivery: Linguistic q-rung orthopair fuzzy Choquet integral approach. *Engineering Applications of Artificial Intelligence*, *126*, 106811.
37. Jana, C., Garg, H., Pal, M., Sarkar, B., & Wei, G. (2024). MABAC framework for logarithmic bipolar fuzzy multiple attribute group decision-making for supplier selection. *Complex & Intelligent Systems*, *10*(1), 273-288.
38. Riaz, M., Farid, H. M. A., Jana, C., Pal, M., & Sarkar, B. (2023). Efficient city supply chain management through spherical fuzzy dynamic multistage decision analysis. *Engineering Applications of Artificial Intelligence*, *126*, 106712.
39. Jana, C., Simic, V., Pal, M., Sarkar, B., & Pamucar, D. (2024). Hybrid multi-criteria decision-making method with a bipolar fuzzy approach and its applications to economic condition analysis. *Engineering Applications of Artificial Intelligence*, *132*, 107837.
40. Seikh, M. R., & Mandal, U. (2022). Q-rung orthopair fuzzy Frank aggregation operators and its application in multiple attribute decision-making with unknown attribute weights. *Granular Computing*, 1-22.
41. Seikh, M. R., & Mandal, U. (2023). q-Rung orthopair fuzzy Archimedean aggregation operators: application in the site selection for software operating units. *Symmetry*, *15*(9), 1680.
42. Mandal, U., & Seikh, M. R. (2023). A Novel Score Function-Based EDAS Method for the Selection of a Vacant Post of a Company with q-Rung Orthopair Fuzzy Data. *Mathematics and Computer Science Volume 1*, 231-250.
43. Seikh, M. R., & Mandal, U. (2022). Multiple attribute group decision making based on quasirung orthopair fuzzy sets: Application to electric vehicle charging station site selection problem. *Engineering Applications of Artificial Intelligence*, *115*, 105299.
44. Seikh, M. R., & Mandal, U. (2022). Multiple attribute decision-making based on 3, 4-quasirung fuzzy sets. *Granular Computing*, 1-14.
45. Zhang, J., Lin, M., Pan, Y., & Xu, Z. (2023). Crftl: cache reallocation-based page-level flash translation layer for smartphones. *IEEE Transactions on Consumer Electronics*.
46. Chen, H., Lin, M., Liu, J., Yang, H., Zhang, C., & Xu, Z. (2024). NT-DPTC: a non-negative temporal dimension preserved tensor completion model for missing traffic data imputation. *Information Sciences*, *653*, 119797.
47. Lin, M., Li, X., & Chen, L. (2020). Linguistic q-rung orthopair fuzzy sets and their interactional partitioned Heronian mean aggregation operators. *International Journal of Intelligent Systems*, *35*(2), 217-249.
48. Lin, M., Huang, C., Chen, R., Fujita, H., & Wang, X. (2021). Directional correlation coefficient measures for Pythagorean fuzzy sets: their applications to medical diagnosis and cluster analysis. *Complex & Intelligent Systems*, *7*, 1025-1043.
49. Lin, M., Li, X., Chen, R., Fujita, H., & Lin, J. (2022). Picture fuzzy interactional partitioned Heronian mean aggregation operators: an application to MADM process. *Artificial Intelligence Review*, 1-38.
50. Garg, H., & Nancy. (2018). Some hybrid weighted aggregation operators under neutrosophic set environment and their applications to multicriteria decision-making. *Applied Intelligence*, *48*, 4871-4888.
51. Torkayesh, A. E., Tavana, M., & Santos-Arteaga, F. J. (2022). A multi-distance interval-valued neutrosophic approach for social failure detection in sustainable municipal waste management. *Journal of Cleaner Production*, *336*, 130409.
52. Nagarajan, D., Gobinath, V. M., & Broumi, S. (2023). Multicriteria Decision Making on 3D printers for economic manufacturing using Neutrosophic environment. *Neutrosophic Sets and Systems*, *57*(1), 3.
53. Garg, H., & Nancy. (2018). Multi-criteria decision-making method based on prioritized Muirhead mean aggregation operator under neutrosophic set environment. *Symmetry*, *10*(7), 280.

54. Riaz, M., Farid, H. M. A., Ashraf, S., & Kamacı, H. (2023). Single-valued neutrosophic fairly aggregation operators with multi-criteria decision-making. *Computational and Applied Mathematics*, *42*(3), 104.

55. Garai, T., Dalapati, S., & Smarandache, F. (2023). Softmax function based neutrosophic aggregation operators and application in multi-attribute decision making problem. *Neutrosophic Sets and Systems*, *56*(1), 16.

56. Ali, S., Ali, A., Azim, A. B., ALoqaily, A., & Mlaiki, N. (2023). Averaging aggregation operators under the environment of q-rung orthopair picture fuzzy soft sets and their applications in MADM problems. *AIMS Mathematics*, *8*(4), 9027-9053.

57. Ali, S., Ali, A., Azim, A. B., Khan, A. S., Awwad, F. A., & Ismail, E. A. (2024). TOPSIS method based on q-rung orthopair picture fuzzy soft environment and its application in the context of green supply chain management. *AIMS Mathematics*, *9*(6), 15149-15171.

58. Azim, A. B., ALoqaily, A., Ali, A., Ali, S., Mlaiki, N., & Hussain, F. (2023). q-Spherical fuzzy rough sets and their usage in multi-attribute decision-making problems. *AIMS Mathematics*, *8*(4), 8210-8248.

59. Azim, A. B., Ali, A., Khan, A. S., Awwad, F. A., Ismail, E. A., & Ali, S. (2024). Assessing indoor positioning system: A q-spherical fuzzy rough TOPSIS analysis. *Heliyon*, *10*(10).

60. Azim, A. B., Ali, A., Khan, A. S., Awwad, F. A., Ismail, E. A., & Ali, S. (2024). Utilizing sine trigonometric q-spherical fuzzy rough aggregation operators for group decision-making and their role in digital transformation. *Heliyon*, *10*(10).

61. Azim, A. B., ALoqaily, A., Ali, A., Ali, S., Mlaiki, N., & Hussain, F. (2023). Industry 4.0 project prioritization by using q-spherical fuzzy rough analytic hierarchy process. *AIMS Mathematics*, *8*(8), 18809-18832.

62. Khan, M. R., Ullah, K., Khan, Q., & Awsar, A. (2023). Some Aczel–Alsina Power Aggregation Operators Based on Complex q-Rung Orthopair Fuzzy Set and Their Application in Multi-Attribute Group Decision-Making. *IEEE Access*.

63. Petchimuthu, S., Palpandi, B., & Senapati, T. (2024). Exploring pharmacological therapies through complex q-rung picture fuzzy Aczel–Alsina prioritized ordered operators in adverse drug reaction analysis. *Engineering Applications of Artificial Intelligence*, *133*, 107996.

Plg report

ORIGINALITY REPORT

10%	7%	9%	2%
SIMILARITY INDEX	INTERNET SOURCES	PUBLICATIONS	STUDENT PAPERS

PRIMARY SOURCES

1	res.mdpi.com<br>Internet Source	1%
2	doaj.org<br>Internet Source	<1%
3	www.aimspress.com<br>Internet Source	<1%
4	www.mdpi.com<br>Internet Source	<1%
5	Submitted to Higher Education Commission Pakistan<br>Student Paper	<1%
6	core.ac.uk<br>Internet Source	<1%
7	"q-Rung Orthopair Fuzzy Sets", Springer Science and Business Media LLC, 2022<br>Publication	<1%
8	Guangyou Zhou, Shangda Yuan, Sumei Luo. "Mesh Simplification Algorithm Based on the Quadratic Error Metric and Triangle Collapse", IEEE Access, 2020<br>Publication	<1%

9	archive.org<br>Internet Source	<1 %
10	ouci.dntb.gov.ua<br>Internet Source	<1 %
11	www.hindawi.com<br>Internet Source	<1 %
12	mdpi-res.com<br>Internet Source	<1 %
13	Chiranjibe Jana, Vladimir Simic, Madhumangal Pal, Biswajit Sarkar, Dragan Pamucar. "Hybrid multi-criteria decision-making method with a bipolar fuzzy approach and its applications to economic condition analysis", Engineering Applications of Artificial Intelligence, 2024<br>Publication	<1 %
14	Rana Muhammad Zulqarnain, Imran Siddique, Aiyared Iampan, Jan Awrejcewicz, Maksymilian Bednarek, Rifaqat Ali, Muhammad Asif. "Novel Multicriteria Decision Making Approach for Interactive Aggregation Operators of q-Rung Orthopair Fuzzy Soft Set", IEEE Access, 2022<br>Publication	<1 %
15	dokumen.pub<br>Internet Source	<1 %
16	Sumbal Ali, Muhammad Rahim, Sanaa A. Bajri, Sadique Ahmad, Rabab Alharbi,	<1 %

Hamiden Abd El-Wahed Khalifa. " Neutrosophic aggregation operators and their applications in the software site selection ", Heliyon, 2024
Publication

17	link.springer.com<br>Internet Source	<1 %
18	sourceforge.net<br>Internet Source	<1 %
19	"Neutrosophic Operational Research", Springer Science and Business Media LLC, 2021<br>Publication	<1 %
20	Yuqin Du, Xiangjun Du, Yuanyuan Li, Jian-xin Cui, Fujun Hou. "Complex q-rung orthopair fuzzy Frank aggregation operators and their application to multi-attribute decision making", Soft Computing, 2022<br>Publication	<1 %
21	matwbn.icm.edu.pl<br>Internet Source	<1 %
22	www.iiu.edu.pk<br>Internet Source	<1 %
23	Ahmad Bin Azim, Asad Ali, Abdul Samad Khan, Fuad A. Awwad, Emad A.A. Ismail, Sumbal Ali. "Utilizing Sine Trigonometric q-Spherical Fuzzy Rough Aggregation Operators for Group Decision-Making and	<1 %

Their Role in Digital Transformation", Heliyon, 2024
Publication

---

24 Azmat Hussain, Muhammad Irfan Ali, Tahir Mahmood, Muhammad Munir. "q-Rung orthopair fuzzy soft average aggregation operators and their application in multicriteria decision-making", International Journal of Intelligent Systems, 2020 <1 %
Publication

---

25 Muhammad Akram, Gulfam Shahzadi, Muhammad Arif Butt, Faruk Karaaslan. "A hybrid decision making method based onq-rung orthopair fuzzy soft information", Journal of Intelligent & Fuzzy Systems, 2021 <1 %
Publication

---

26 Muhammad Saeed, Abdul Wahab, Mubashir Ali, Jawad Ali, Ebenezer Bonyah. "An innovative approach to passport quality assessment based on the possibility q-rung ortho-pair fuzzy hypersoft set", Heliyon, 2023 <1 %
Publication

---

27 Chiranjibe Jana, Momčilo Dobrodolac, Vladimir Simic, Madhumangal Pal, Biswajit Sarkar, Željko Stević. "Evaluation of sustainable strategies for urban parcel delivery: Linguistic q-rung orthopair fuzzy Choquet integral approach", Engineering Applications of Artificial Intelligence, 2023 <1 %
Publication

28 Harish Garg, Nancy. "Some hybrid weighted aggregation operators under neutrosophic set environment and their applications to multicriteria decision-making", Applied Intelligence, 2018
Publication
<1%

29 Hong Chen, Mingwei Lin, Jiaqi Liu, Hengshuo Yang, Chao Zhang, Zeshui Xu. "NT-DPTC: A non-negative temporal dimension preserved tensor completion model for missing traffic data imputation", Information Sciences, 2024
Publication
<1%

30 Jun Wang, Runtong Zhang, Li Li, Xiaomin Zhu, Xiaopu Shang. "A novel approach to multi-attribute group decision making based on q-rung orthopair uncertain linguistic information", Journal of Intelligent & Fuzzy Systems, 2019
Publication
<1%

31 Tapas Kumar Paul, Chiranjibe Jana, Madhumangal Pal, Vladimir Simic. "Sustainable carbon-dioxide storage assessment in geological media using modified Pythagorean fuzzy VIKOR and DEMATEL approach", International Journal of Hydrogen Energy, 2022
Publication
<1%

32 Bahram Farhadinia. "Hesitant Fuzzy Set", Springer Science and Business Media LLC,
<1%

2021
Publication

33 Harish Garg, Gulfam Shahzadi, Muhammad Akram. "Decision-Making Analysis Based on Fermatean Fuzzy Yager Aggregation Operators with Application in COVID-19 Testing Facility", Mathematical Problems in Engineering, 2020 <1 %
Publication

34 Muhammad Rahim, Fazli Amin, ElSayed M. Tag Eldin, Hamiden Abd El-Wahed Khalifa, Sadique Ahmad. "p, q-Spherical fuzzy sets and their aggregation operators with application to third-party logistic provider selection", Journal of Intelligent & Fuzzy Systems, 2023 <1 %
Publication

35 Rana Muhammad Zulqarnain, Hong-Liang Dai, Wen-Xiu Ma, Imran Siddique, Sameh Askar, Hamza Naveed. "Supplier selection in green supply chain management using correlation-based TOPSIS in a q-rung orthopair fuzzy soft environment", Heliyon, 2024 <1 %
Publication

36 Harish Garg, Zeeshan Ali, Tahir Mahmood. "Generalized dice similarity measures for complex q-Rung Orthopair fuzzy sets and its application", Complex & Intelligent Systems, 2020 <1 %
Publication

37 Muhammad Rahim, Shougi Suliman Abosuliman, Roobaea Alroobaea, Kamal Shah, Thabet Abdeljawad. "Cosine Similarity and Distance Measures for p,q,– Quasirung Orthopair fuzzy Sets: Applications in Investment Decision-Making", Heliyon, 2024
Publication
<1 %

38 R. G. Zaripov. "Evolution of the difference information in the process of the Fermi and Bose gas self-organization for nonextensive systems", Russian Physics Journal, 2009
Publication
<1 %

39 digitalcommons.lasalle.edu
Internet Source
<1 %

40 www.researchgate.net
Internet Source
<1 %

41 Benting Wan, Juelin Huang, Xi Chen, Youyu Cheng, Jing Wang. "Interval-Valued q-Rung Orthopair Fuzzy Choquet Integral Operators and Their Application in Group Decision-Making", Mathematical Problems in Engineering, 2022
Publication
<1 %

42 Xindong Peng, Ganeshsree Selvachandran. "Pythagorean fuzzy set: state of the art and future directions", Artificial Intelligence Review, 2017
Publication
<1 %

export.arxiv.org

43 Internet Source <1%

44 Muhammad Akram, Anam Luqman. "Fuzzy Hypergraphs and Related Extensions", Springer Science and Business Media LLC, 2020
Publication <1%

45 Surya Annamalai, Vimala Jayakumar. "Medical diagnosis based on lattice ordered q-rung linear diophantine fuzzy hypersoft matrices", Journal of Intelligent & Fuzzy Systems, 2024
Publication <1%

46 Tapas Kumar Paul, Madhumangal Pal, Chiranjibe Jana. "Portfolio selection as a multicriteria group decision making in Pythagorean fuzzy environment with GRA and FAHP framework", International Journal of Intelligent Systems, 2021
Publication <1%

47 Touqeer Ahmad, Muhammad Rahim, Jie Yang, Rabab Alharbi, Hamiden Abd El-Wahed Khalifa. "Development of p,q -quasirung orthopair fuzzy hamacher aggregation operators and its application in decision-making problems", Heliyon, 2024
Publication <1%

48 discovery.researcher.life
Internet Source <1%

49 ebin.pub <1 %
Internet Source

50 Azmat Hussian, Tahir Mahmood, Muhammad Irfan Ali, Vassilis C. Gerogiannis, Dimitrios Tzimos, Dimitrios Giakovis. "q-Rung orthopair fuzzy soft Hamacher aggregation operators and their applications in multi-criteria decision making", Computational and Applied Mathematics, 2023 <1 %
Publication

51 Dimple Rani, Harish Garg. "Some modified results of the subtraction and division operations on interval neutrosophic sets", Journal of Experimental & Theoretical Artificial Intelligence, 2019 <1 %
Publication

52 Donghai Liu, Yuanyuan Liu, Lizhen Wang. "The reference ideal TOPSIS method for linguistic q-rung orthopair fuzzy decision making based on linguistic scale function", Journal of Intelligent & Fuzzy Systems, 2020 <1 %
Publication

53 Jian-Ping Fan, Rui Cheng, Mei-Qin Wu. "Extended EDAS Methods for Multi-Criteria Group Decision-Making Based on IV-CFSWAA and IV-CFSWGA Operators with Interval-Valued Complex Fuzzy Soft Information", IEEE Access, 2019 <1 %
Publication

54 Jie Wang, Guiwu Wei, Cun Wei, Yu Wei. "Dual Hesitant q-Rung Orthopair Fuzzy Muirhead Mean Operators in Multiple Attribute Decision Making", IEEE Access, 2019 <1 %
Publication

---

55 Rana Muhammad Zulqarnain, Rifaqat Ali, Jan Awrejcewicz, Imran Siddique, Fahd Jarad, Aiyared Iampan. "Some Einstein Geometric Aggregation Operators for q-Rung Orthopair Fuzzy Soft Set With Their Application in MCDM", IEEE Access, 2022 <1 %
Publication

---

56 Ronnason Chinram, Azmat Hussian, Muhammad Irfan Ali, Tahir Mahmood. "Some geometric aggregation operators under q-Rung orthopair fuzzy soft information with their applications in multi-criteria decision making", IEEE Access, 2021 <1 %
Publication

---

57 Xiaofei Zhao, Guiwu Wei. "Some intuitionistic fuzzy Einstein hybrid aggregation operators and their application to multiple attribute decision making", Knowledge-Based Systems, 2013 <1 %
Publication

---

58 biomed.bas.bg <1 %
Internet Source

---

59 downloads.hindawi.com <1 %
Internet Source

60	fs.unm.edu<br>Internet Source	<1 %
61	ia601503.us.archive.org<br>Internet Source	<1 %
62	ia601604.us.archive.org<br>Internet Source	<1 %
63	www.techscience.com<br>Internet Source	<1 %
64	Harish Garg. "Novel neutrality aggregation operator-based multiattribute group decision-making method for single-valued neutrosophic numbers", Soft Computing, 2019<br>Publication	<1 %
65	Sumbal Ali, Asad Ali, Ahmad Bin Azim, Ahmad ALoqaily, Nabil Mlaiki. "Averaging aggregation operators under the environment of *q*-rung orthopair picture fuzzy soft sets and their applications in MADM problems", AIMS Mathematics, 2023<br>Publication	<1 %
66	Yang Yang, Chao Zhang. "MULTIMOORA Method-Based Schweizer–Sklar Operations for CO2 Geological Storage Site Selection Under Pythagorean Fuzzy Environment", International Journal of Computational Intelligence Systems, 2023<br>Publication	<1 %

67	Abdul Wahab, Jawad Ali, Muhammad Bilal Riaz, Muhammad Imran Asjad, Taseer Muhammad. "A novel probabilistic q-rung orthopair linguistic neutrosophic information-based method for rating nanoparticles in various sectors", Scientific Reports, 2024<br>Publication	<1 %
68	Ahmad Bin Azim, Asad Ali, Abdul Samad Khan, Fuad A. Awwad, Emad A.A. Ismail, Sumbal Ali. "Assessing indoor positioning system: A q-spherical fuzzy rough TOPSIS analysis", Heliyon, 2024<br>Publication	<1 %
69	Awais Younus, Muhammad Asif, Usama Atta, Tehmina Bashir, Thabet Abdeljawad. "Applications of fuzzy conformable Laplace transforms for solving fuzzy conformable differential equations", Soft Computing, 2023<br>Publication	<1 %
70	Madhumangal Pal. "Recent Developments of Fuzzy Matrix Theory and Applications", Springer Science and Business Media LLC, 2024<br>Publication	<1 %
71	Tapas Kumar Paul, Chiranjibe Jana, Madhumangal Pal. "Multi-criteria group decision-making method in disposal of municipal solid waste based on cubic	<1 %

Pythagorean fuzzy EDAS approach with incomplete weight information", Applied Soft Computing, 2023
Publication

72	Xindong Peng, Zhigang Luo. "A review of q-rung orthopair fuzzy information: bibliometrics and future directions", Artificial Intelligence Review, 2021<br>Publication	<1%
73	Yuan Xu, Xiaopu Shang, Jun Wang, Wen Wu, Huiqun Huang. "Some q-Rung Dual Hesitant Fuzzy Heronian Mean Operators with Their Application to Multiple Attribute Group Decision-Making", Symmetry, 2018<br>Publication	<1%

Exclude quotes	On	Exclude matches	Off
Exclude bibliography	On		
